# *De novo* identification of lipid II binding lipopeptides with antibacterial activity against vancomycin-resistant bacteria†
†Electronic supplementary information (ESI) available: Synthetic procedures and analytical data for all new compounds. Supporting schemes, figures, and tables. Description of phage-selection and analysis, biochemical and biological assays. See DOI: 10.1039/c7sc03413j


**DOI:** 10.1039/c7sc03413j

**Published:** 2017-10-02

**Authors:** Peter 't Hart, Thomas M. Wood, Kamaleddin Haj Mohammad Ebrahim Tehrani, Roel M. van Harten, Małgorzata Śleszyńska, Inmaculada Rentero Rebollo, Antoni P. A. Hendrickx, Rob J. L. Willems, Eefjan Breukink, Nathaniel I. Martin

**Affiliations:** a Department of Chemical Biology & Drug Discovery , Utrecht Institute for Pharmaceutical Sciences , Utrecht University , Universiteitsweg 99 , 3584 CG Utrecht , The Netherlands . Email: n.i.martin@uu.nl; b Institute of Chemical Sciences and Engineering , Ecole Polytechnique Fédérale de Lausanne , Lausanne , Switzerland; c Department of Medical Microbiology , University Medical Center , Utrecht , The Netherlands; d Membrane Biochemistry and Biophysics Group , Department of Chemistry , Utrecht University , The Netherlands

## Abstract

Creative strategies for identifying new antibiotics are essential to addressing the looming threat of a post-antibiotic era. We here report the use of a targeted peptide phage display screen as a means of generating novel antimicrobial lipopeptides. Specifically, a library of phage displayed bicyclic peptides was screened against a biomolecular target based on the bacterial cell wall precursor lipid II. In doing so we identified unique lipid II binding peptides that upon lipidation were found to be active against a range of Gram-positive bacteria including clinically relevant strains of vancomycin resistant bacteria. Optimization of the peptide sequence led to variants with enhanced antibacterial activity and reduced hemolytic activity. Biochemical experiments further confirm a lipid II mediated mode of action for these new-to-nature antibacterial lipopeptides.

## Abstract 2

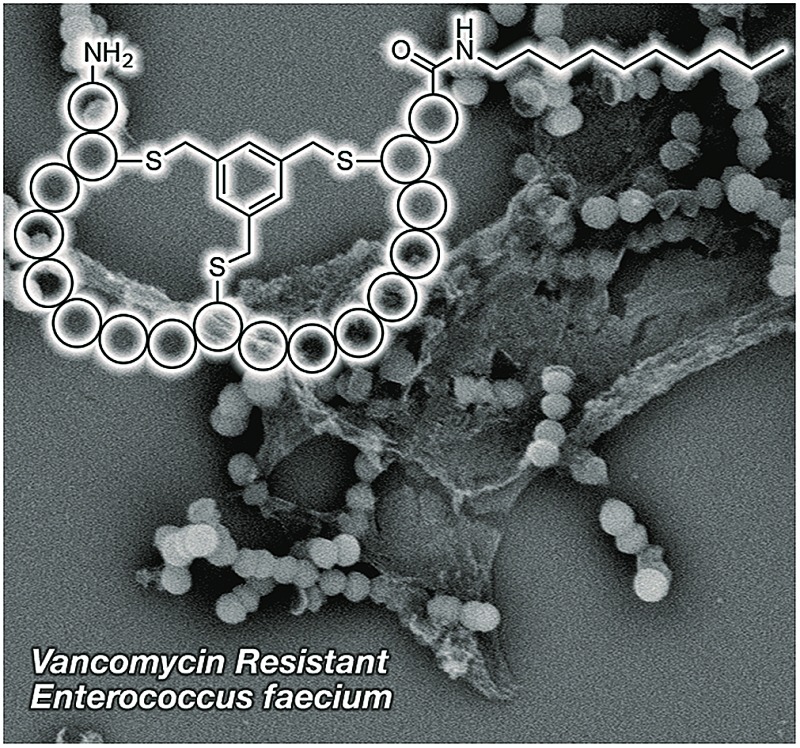
Lipid II binding lipopeptides discovered *via* bicyclic peptide phage display exhibit promising antibacterial activity.

## Introduction

The rapidly increasing incidence of drug resistant bacteria underscores the importance of employing new strategies for the discovery of novel antimicrobial compounds. The search for antibiotics has typically relied upon natural sources such as fungi and soil microbes.^1^ While such organisms provide a wide range of active compounds, competing strains have also coevolved resistance genes against most antimicrobial substances.^1^–^3^ In this way, the so-called “resistome” represents a natural pool of genes, that when incorporated into transferable genetic elements like plasmids, can confer resistance to clinically-relevant strains.^1^,^2^ Further complicating today's search for antimicrobials from natural sources is the re-isolation of known scaffolds. In light of these challenges, we were drawn to the use of peptide phage display as a means of exploring previously untapped chemical space while also avoiding the resistome issue. Peptide phage display is a selection technique that can generate high affinity peptide ligands for a (bio)molecular target.^4^ Historically, peptide phage-display techniques have not been widely applied in the search for new antibiotics. Some reports have described the use of phage-display in the identification of peptides that bind to intracellular bacterial targets such as the ribosome or enzymes involved in cell wall synthesis.^5^–^10^ However, despite binding to their intended targets, none of the peptides identified in these investigations displayed antimicrobial activity, an outcome likely due to the poor cellular penetration common to peptides. Alternatively, “whole cell” approaches have also been described wherein a peptide phage-display library is screened against the cell surface of a target organism.^11^–^14^ Such strategies typically yield positively charged peptides with antibacterial activity attributed to general bacterial membrane disruption rather than binding to a specific biomolecular target.

**Fig. 1:**
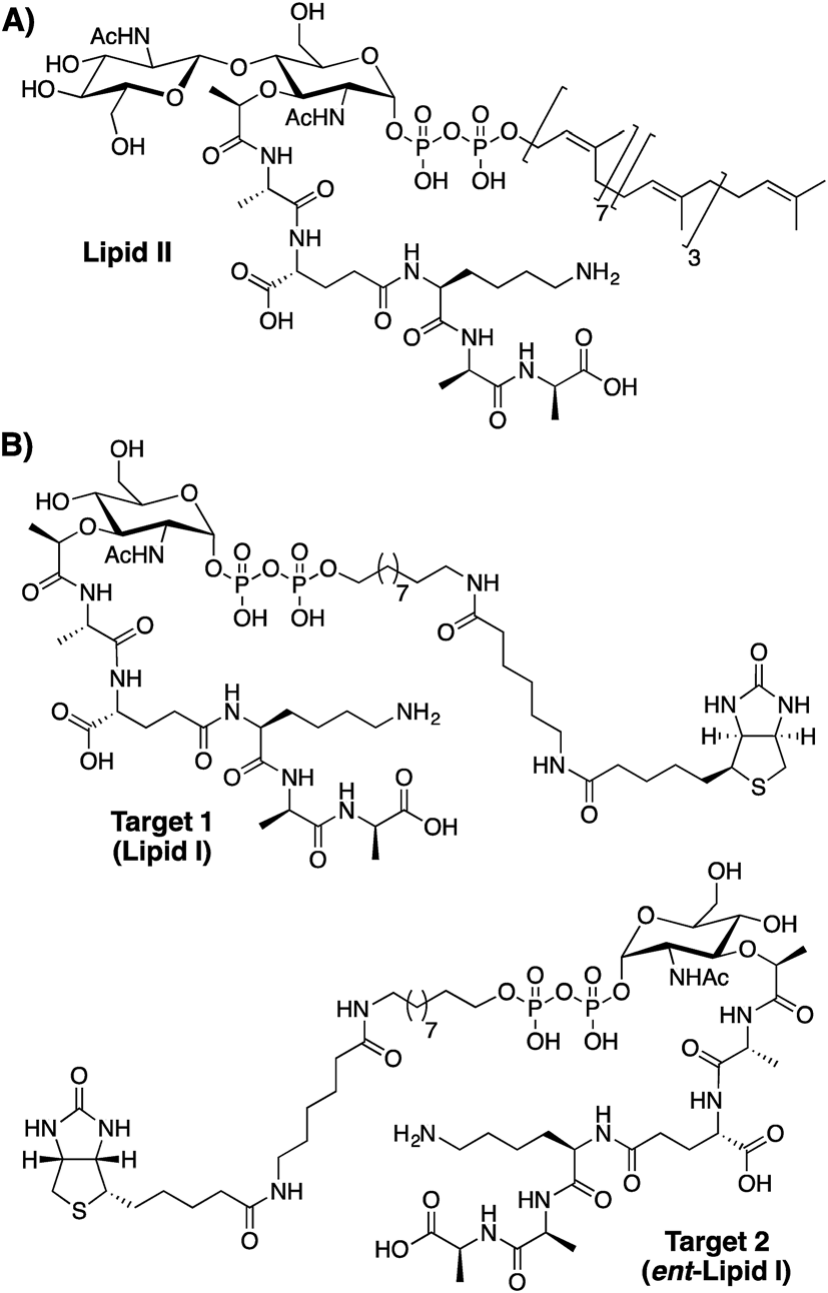
Structures of (A) lipid II, the bacterial cell wall precursor and (B) lipid I analogues used as targets for peptide phage display screening.

For the peptide phage display screens here described we opted for a targeted approach aimed at identifying peptides capable of binding to the bacterial cell wall precursor lipid II (Fig. 1). Unique to bacteria, lipid II is an established target for numerous antibiotics.^15^,^16^ Biosynthesized on the inner surface of the bacterial membrane, lipid II must be translocated to the extracellular surface before it can be incorporated into the cell wall. Thus, in Gram-positive bacteria, lipid II is accessible to peptide antibiotics, while the outer membrane of Gram-negative strains generally prevents access.^16^ Vancomycin is the best-studied example of a therapeutically used lipid II binding antibiotic and clearly illustrates the potential for targeting this key bacterial building block. For the purposes of our phage display screens we opted to employ a synthetically more tractable target molecule based upon lipid I, the biosynthetic precursor of lipid II. This choice was also based on the knowledge that many lipid II targeting antibiotics bind lipid I and II with similar affinities by exploiting key structural elements common to both.^15^–^19^ Most notable in this regard is the pentapeptide unit targeted by the glycopeptides antibiotics and the pyrophosphate moiety targeted by type A lantibiotics and the recently reported teixobactin.^18^

As illustrated in Fig. 1 our phage display screens made use of lipid I inspired targets in both natural and enantiomeric forms (target **1** and target **2** respectively). The rationale for screening against both natural and enantiomeric analogues was two-fold. First, by doing so the amount of chemical space explored in the screen is effectively doubled. Secondly, it was considered that l-peptides selected in the screen that possess high affinity for natural lipid I/II might in fact be toxic towards the *E. coli* used in the amplification step of the phage display experiment. Like all bacteria *E. coli* also uses lipid I/II in constructing its cell wall. For this reason, the use of an enantiomeric lipid I/II target (target 2) was viewed as a way of avoiding any such negative selection bias. Using this approach, l-peptides identified with affinity for the enantiomeric target would then be chemically synthesized as the corresponding d-peptides, which, by symmetry arguments would be expected bind with equal affinity to native lipid I/II. Such so-called “mirror-image” strategies have also previously been employed in identifying d-peptide ligands for various targets including HIV-1 gp41.^20^,^21^

## Results and discussion

### Target synthesis

**Scheme 1:**
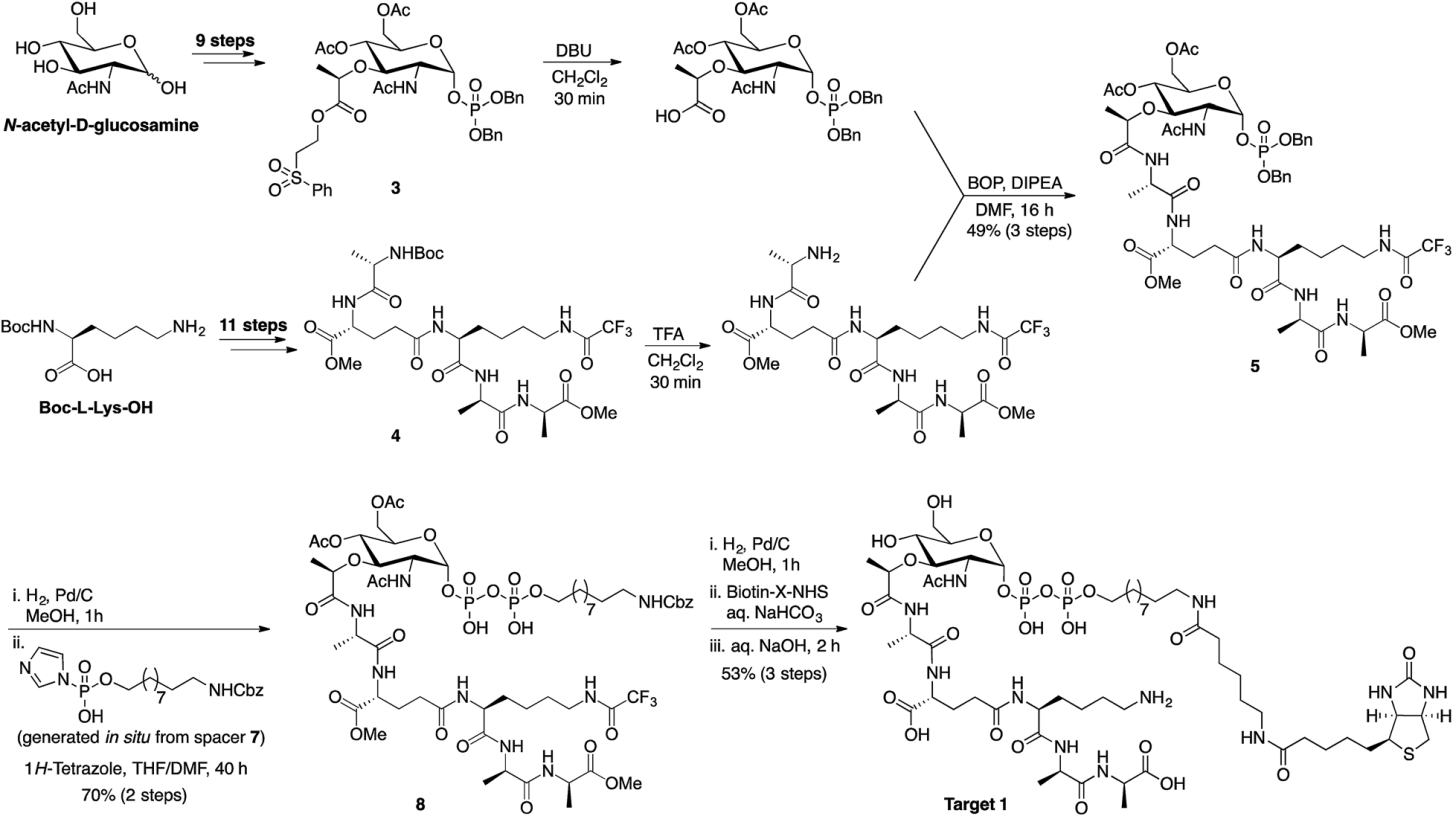
Synthetic route employed in preparing lipid I inspired targets for use in bicyclic peptide phage display screening. Advanced intermediates **3** and **4** were prepared as previously described.^19^,^20^ For the synthesis of spacer **7** see ESI Scheme S2.†

The synthetic route followed in preparing the lipid I inspired targets for use in the phage-display screens was adapted from the total syntheses of lipid I and II previously reported by VanNieuwenhze and coworkers (Scheme 1).^22^,^23^ The protected muramic acid species **3** was prepared in nine steps from *N*-acetyl-d-glucosamine while pentapeptide **4** was prepared in eleven steps starting from boc-l-lysine. Building blocks **3** and **4** were deprotected and coupled to provide intermediate **5** in good yield. Following debenzylation, spacer **7** was incorporated with concomitant installation of the pyrophosphate moiety to give compound 8. Hydrogenation, biotinylation and global deprotection followed by HPLC purification yielded final target 1. The inclusion of a biotin tag in the target was needed for subsequent use in the phage display experiment. The Walker group has previously described the preparation of biotinylated lipid II analogues wherein a biotin group was linked *via* the pentapeptide.^24^,^25^ However, for our purposes we wanted to ensure the pentapeptide was present in its native form so as to ensure binding possibilities in the phage display screens. We therefore employed a different design strategy wherein the undecaprenol lipid was replaced with a spacer terminating in the biotin tag. This choice was made given that in its biological context the undecaprenol lipid of lipid II is embedded in the bacterial membrane and is therefore not expected to influence the binding of peptide ligands.^15^ The mirror image target **2** was prepared using the same synthetic route shown in Scheme 1 but employing the corresponding enantiomeric building blocks. Given that *N*-acetyl-l-glucosamine is not commercially available it was prepared on gram scale following a four-step synthesis from l-arabinose (see ESI, Scheme S4†).

### Bicyclic peptide phage display

**Fig. 2:**
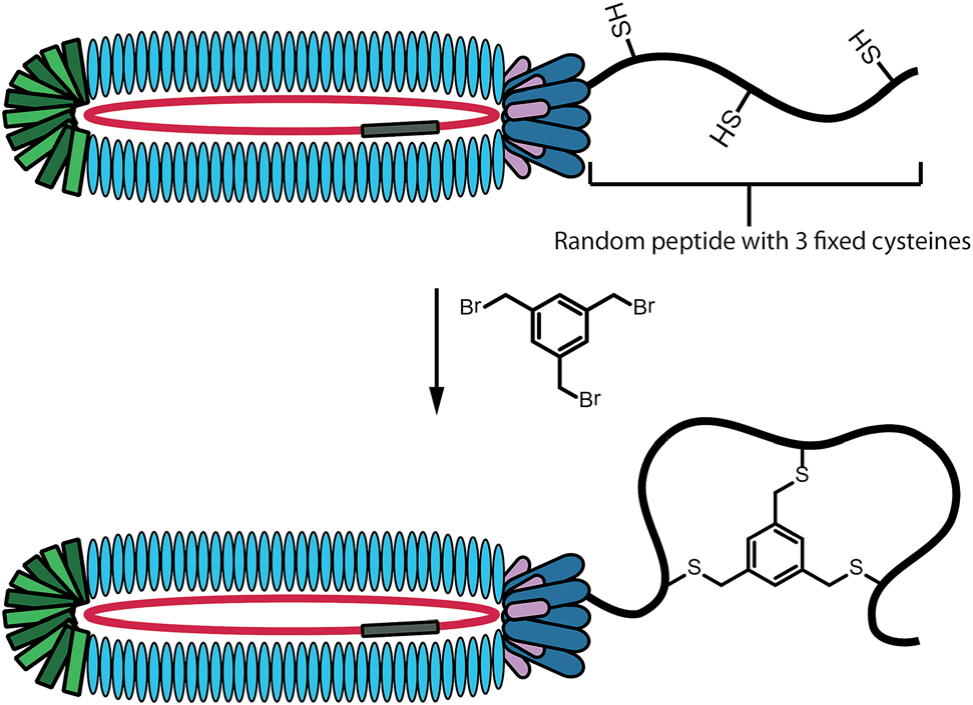
Schematic representation of peptide bicyclization on phage surface for use in bicyclic peptide phage display.

In performing the phage display screens against targets **1** and **2** we employed the bicyclic variant of the phage display technique developed by Heinis and Winter.^26^ To do so, the peptides displayed in the phage library (consisting of an estimated 4.4 × 10^9^ unique sequences) are specifically designed to contain three cysteine residues at fixed positions. By treating the pool of intact phage with 1,3,5-tris(bromomethyl)benzene (TBMB), bicyclic peptides are displayed on the phage surface (Fig. 2).^26^,^27^ Constraining the peptides as the bicyclic species can result in a lower entropic barrier to target binding relative to the linear peptide counterpart and can significantly enhance binding affinity.^26^,^27^ In addition, many natural targeted antimicrobial peptides contain cyclic structures (daptomycin, vancomycin, nisin, *etc.*) further suggesting that such an approach may be of value in the identification of novel antibacterials.

The bicyclic phage display technique was initially performed following the standard protocols for identifying peptide ligands for large protein targets.^28^ These initial screens resulted primarily in the identification of peptides bearing a HPQ motif, a known streptavidin binding sequence (data not shown).^29^ Such interfering sequences can arise when the target in the phage display experiment is much smaller than the streptavidin used for binding the biotin tag or when low affinity binding sequences are obtained. After several rounds of optimization we found that by incubating the target with the phage library in solution at low temperature (4 °C) and for a longer time (16 hours), followed by a quick capture with streptavidin beads allowed for the identification of unique binding sequences. After two rounds of biopanning using these conditions the phage DNA was analyzed both by isolation of single phage clones and by high-throughput sequencing of the entire phage pools.^30^ The sequencing results thus obtained are shown in Table 1. Selection against target **1** resulted in a variety of peptide sequences (P1–P7) with only moderate enrichment. However, in the screen performed with enantiomeric target **2** a very strong enrichment for a specific peptide sequence (P8) was found.

**Table 1:** Results of phage display screening against lipid I analogues target **1** and target **2** after two rounds of biopanning^*a*^

Peptide	Sequence	Sequencing results		MIC^*c*^
		HTS^*b*^	Manual	
**Target 1**				
P1	ACDWPDWQCYGWSSHCG	4.2%	n.o.	n.a.
P2	ACKHQAQECVAIREGCG	3.1%	n.o.	n.a.
P3	ACWAMPMWCDSWSQNCG	1.8%	1/10	n.a.
P4	ACRPQKFNCISANIRCG	1.5%	n.o.	64
P5	ACKAMIGACVAMQFACG	1.2%	n.o.	n.o.
P6	ACYPVDWYCLFQTVDCG	1.1%	n.o.	128
P7	ACRYVSGDCYYAQAHCG	1.0%	n.o.	n.a.

**Target 2**				
P8	ACLLQSLLCPYSTHRCG	**97.7%**	**7/7**	128

^*a*^n.o. = not observed, n.a. = no activity (highest tested concentration was 512 μg mL^–1^ for all peptides).

^*b*^Peptide abundance in entire phage pool after filtering of incorrect sequences (see ESI for unfiltered data).

^*c*^MIC (μg mL^–1^) against *M. luteus* in a broth microdilution assay.

### Evaluation of antibacterial activity and optimization studies

**Fig. 3:**
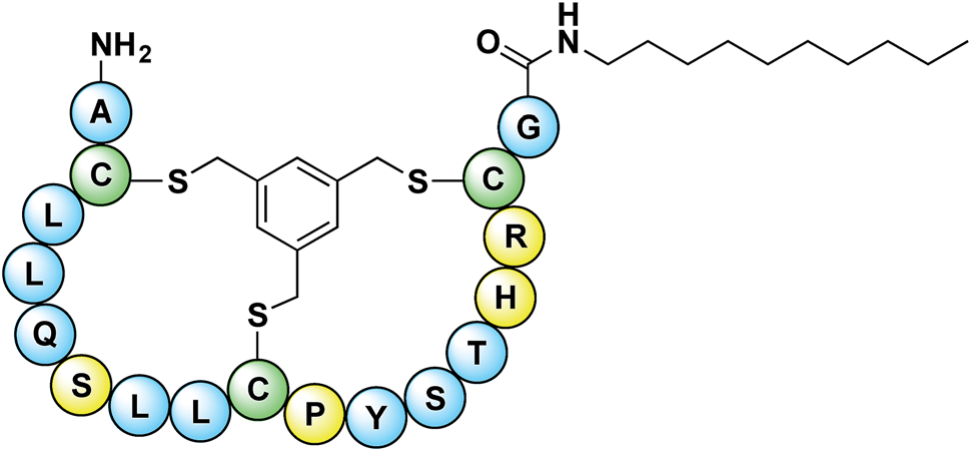
Structure of lead P8-d-C_10_ lipopeptide. Peptide sequence derived from hit identified using bicyclic peptide phage display screen with enantiomeric lipid I target 2. Conserved cysteine residues needed for cyclization highlighted in green. Amino acids in yellow are amenable to mutation as identified by alanine scan.

The peptide sequences identified from the phage-display screen (P1–P8, Table 1) were next prepared synthetically. Linear precursor peptides were generated *via* solid phase peptide synthesis as the C-terminal amides and then cyclized in solution by treatment with TBMB to introduce the bicyclic framework. Of note, peptide P8 was synthesized using d-amino acids given that it was identified using the enantiomeric target 2. All eight bicyclic peptides were tested for antimicrobial activity against *Micrococcus luteus*, an established indicator strain. This preliminary activity screen revealed moderate activity (MIC values of 64–128 μg mL^–1^) for peptides P4, P6, and P8. As a means of increasing the activity of these peptides we employed a strategy recently reported by our group, wherein lipidation was found to enhance the activity of the otherwise poorly active bicyclic A/B-ring system of nisin.^31^ The nisin A/B-ring system (residues 1–12) is known to bind lipid II, but displays little-to-no antimicrobial activity.^31^,^32^ The introduction of a C-terminal lipid restores antibacterial activity to near that of the full-length nisin peptide.^31^ To investigate whether such an approach would improve the activity of peptides P4, P6, and P8 they were each prepared with a 10-carbon saturated lipid at the N- or C-terminus. In all cases C-terminal lipidation significantly improved antibacterial activity resulting in MIC values ranging from 2–16 μg mL^–1^. By comparison, N-terminal lipidation provided less benefit in enhancing antibacterial activity (see ESI, Table S6†). Of particular note was the finding that for the P8 d-peptide, C-terminal lipidation led to potent activity (MIC 4 μg mL^–1^) against a vancomycin resistant *Enterococcus faecium* strain (VRE155). For this reason we chose to narrow our focus and more thoroughly investigate P8 as a lead compound. A broader panel of C-terminally lipidated P8 variants was prepared with lipids ranging from 6 to 14 carbon atoms. The MICs of these peptides were determined against a number of Gram-positive bacteria including a broader panel of vancomycin-resistant strains (Table 2). When tested against *E. coli* as a representative Gram-negative strain, the compounds displayed no antibacterial activity. This is likely due to the outer membrane present in Gram-negative bacteria which can prevent access to lipid II binding antibiotics. In addition, the hemolytic properties of the lipopeptides were evaluated (see Table 2 and ESI, Table S7†). From these studies the P8-d-C_10_ lipopeptide (Fig. 3) exhibited the most promising combination of antibacterial activity and low hemolytic activity relative to the other lipopeptide variants prepared and tested.

**Table 2:** Antibacterial activity of lipopeptides identified against various Gram-positive bacteria including a panel of vancomycin-resistant strains^*a*^

Peptide	*M. luteus*, (NCTC2665)	*B. subtilis*, (168)	*S. pyogenes*, (5448)	*S. epidermidis*, (1587)	*E. faecium*, (E980)	*E. faecium*, (VRE155)^*b*^	*E. faecium*, (VRE0321)^*b*^	*E. faecium*, (VRE2297)^*b*^	*E. faecium*, (VRE7391)^*b*^	Hemolysis^*c*^, (%)
P8-d-C_10_	4	8–16	8	64	32	4	4	8–16	8	6
P8-d-(R15A)-C_10_	4	>64	16	>64	64	16	8–16	>64	>64	0
P8-d-(H14R, R15A)-C_10_	4	16	16	>64	32	8–16	8–16	16	8–16	2
P8-d-(P10R, R15A)-C_10_	4	8	16	>64	32	8	16	32	16	0
P8-d-(S6R, R15A)-C_10_	4	8	8–16	64	32	8	8	16	16	0
P8-l-(S6R, R15A)-C_10_	4	8	8	32	32	8	8	16	16	0
Vancomycin	<1	<1	<1	2	<1	64	>64	>64	>64	ND
Nisin	<1	4	<1	4	32	<1	1	8	2	ND

^*a*^MIC values reported in units of μg mL^–1^ see ESI Table S9 for MIC values expressed in μM concentration.

^*b*^Vancomycin-resistant *Enterococcus faecium* isolates from hospitalized patients.

^*c*^Hemolysis measured after 1 hour incubation at 37 °C with lipopeptides applied at 32 μg mL^–1^.

To assess the contribution of the individual amino acids in the P8 peptide to its antimicrobial activity, an alanine scan was also performed on the loop residues (3–8 and 10–15). For the purpose of the alanine scan, non-lipidated P8 variants were prepared and evaluated so that any changes in activity could be attributed to differences in the presumed lipid II binding, apart from any membrane mediated effects conferred by the lipid. The alanine scan (summarized in ESI Table S8†) revealed that Arg_15_, His_14_, Pro_10_, and Ser_6_ could be substituted for alanine without negatively impacting antibacterial activity. The finding that Arg_15_ could be mutated to alanine was seen as potentially beneficial in light of the slight hemolytic activity observed with the P8-d-C_10_ lipopeptide. In this regard, we prepared the C-terminally lipidated P8-R15A-C_10_ species and assessed both its hemolytic and antibacterial activity. This peptide was found to be completely non-hemolytic and retained activity against *M. luteus*. However, it was also found to be significantly less active than the original P8-d-C_10_ peptide against other Gram-positive bacteria including vancomycin-resistant strains (Table 2). These findings suggested that the positive charge present in Arg_15_ might contribute to enhancing antibacterial activity. We therefore next prepared three different “double mutants” based on the results of the alanine scan wherein the arginine was reintroduced at the positions found amenable to mutation (His_14_, Pro_10_, and Ser_6_) and Arg_15_ was replaced by alanine. Gratifyingly, when prepared and tested as the C-terminally C_10_ lipidated species, the antimicrobial activity of these double mutant peptides was found to be very similar to that of the original P8-d-C_10_ lipopeptide (Table 2). Furthermore, the P8-d-(P10R, R15A)-C_10_ and P8-d-(S6R, R15A)-C_10_ peptides exhibited no detectable hemolysis at 32 μg mL^–1^.

### Lipid II mediated mechanism of action

To assess the role of lipid II in the mode of action of the lipopeptides a lipid II antagonization assay was employed.^18^,^33^ This assay was performed by premixing P8-d-C_10_ or the optimized P8-d-(S6R, R15A)-C_10_ with an excess of lipid II prior to addition of a live culture of *M. luteus*. In both cases, pre-incubation of the peptides with lipid II resulted in antagonization of antibacterial activity as also observed for a control (nisin) indicating the peptides bind lipid II (ESI Table S10†). To probe the stereochemical aspects of lipid II recognition by the optimized P8-d-(S6R, R15A)-C_10_ lipopeptide we also prepared and tested the corresponding enantiomeric l-peptide species P8-l-(S6R, R15A)-C_10_. We were initially surprised to find that the two enantiomers exhibit essentially the same spectrum of antibacterial activity (Table 2). Given that lipid II is a chiral biomolecule we had expected the enantiomeric lipopeptides to have different activities based on the assumption that they would interact differently with lipid II. To further investigate this phenomenon we employed the same lipid II antagonization assay described above. In doing so we observed that the activity of l-peptide P8-l-(S6R, R15A)-C_10_ was also effectively antagonized by the addition of lipid II indicating that both enantiomers are able to bind lipid II. To explain this observation we hypothesize that the two enantiomeric peptides interact primarily with the pyrophosphate moiety of lipid II and are therefore less influenced by the chiral elements present elsewhere in the lipid II molecule. The pyrophosphate group is known to be a binding site for many natural occurring lipid II binding peptides.^15^,^16^ Interestingly, support for this explanation is provided in a recent report describing the synthesis of enantiomeric teixobactin analogues that were found to be equally active.^34^ While teixobactin is known to bind the pyrophosphate unit,^18^ the activity of the enantiomeric analogues indicates the other structural features present in lipid II do not significantly contribute to binding. Furthermore, in another study our group recently found that nisin binds the achiral undecaprenyl pyrophosphate (C_55_-PP) precursor of lipid II with a *K*_d_ value of 133 nM, an affinity only 10-fold lower than measured for intact lipid II.^19^ To test the hypothesis that both the P8-d-(S6R, R15A)-C_10_ and P8-l-(S6R, R15A)-C_10_ lipopeptides interact with the pyrophosphate moiety we performed an additional antagonizaiton experiment using C_55_-PP. This investigation revealed that indeed, adding C_55_-PP to either the d- or l-lipopeptide resulted in complete loss of antibiotic activity (ESI Table S10†), supporting a working mechanism involving pyrophosphate binding.

### Impact on bacterial cell wall biosynthesis

**Fig. 4:**
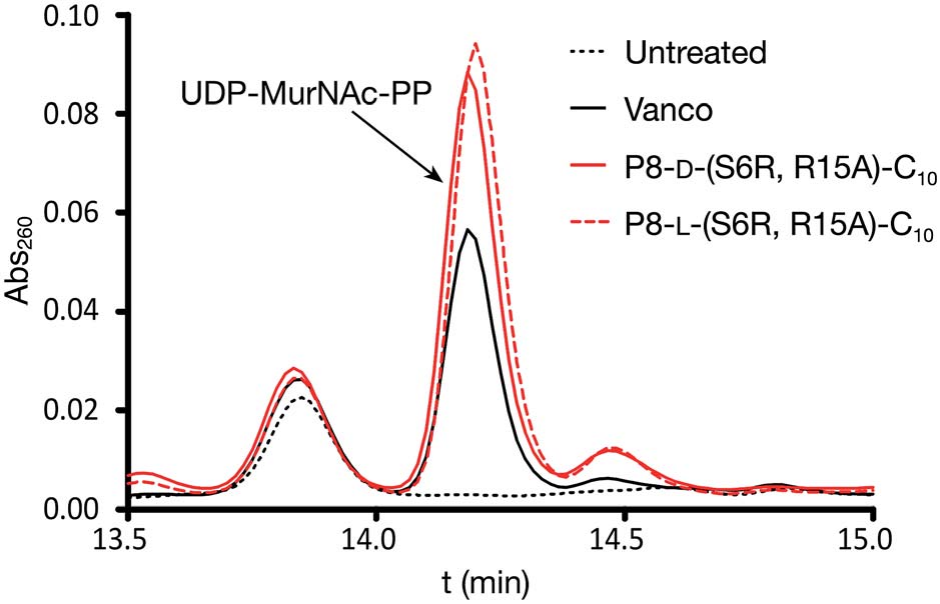
Accumulation of UDP-MurNAc-pentapeptide in *E. faecium* E980 treated with P8-d-(S6R, R15A)-C_10_ or P8-l-(S6R, R15A)-C_10_ lipopeptides. Vancomycin used as a positive control. Identity of the UDP-MurNAc-PP peak confirmed by LCMS and by comparison with pure reference material. Data based on triplicate measurements.

**Fig. 5:**
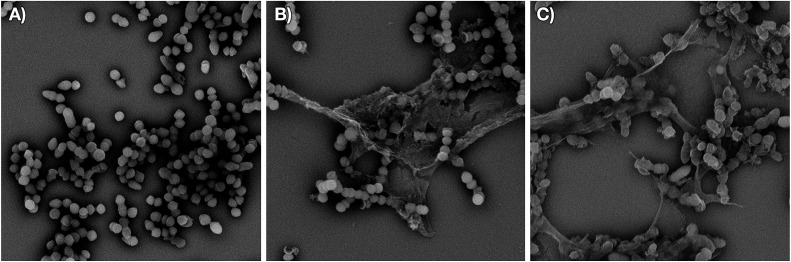
Structural changes in vancomycin-resistant *E. faecium* E155 upon treatment with P8-d-(S6R, R15A)-C_10_ or nisin. Scanning electron micrographs of: (A) untreated cells (scanned at 7500× magnification), (B) cells treated with P8-d-(S6R, R15A)-C_10_ at 2 × MIC (scanned at 8600× magnification), (C) cells treated with nisin at 2 × MIC (scanned at 7700× magnification).

We next examined the effect of the optimized P8-d-(S6R, R15A)-C_10_ lipopeptide on cell wall biosynthesis in live bacteria. Specifically, we employed an assay where the accumulation of the soluble intracellular lipid II precursor UDP-MurNAc-pentapeptide is measured in response to antibiotic administration. Antibiotics known to interfere with cell wall biosynthesis by sequestration of lipid II (*i.e.* vancomycin) can lead to UDP-MurNAc-pentapeptide accumulation in the cytosol which can then be extracted and assessed by chromatographic means.^18^,^33^ Incubation of *E. faecium* E980 with P8-d-(S6R, R15A)-C_10_ or the enantiomeric P8-l-(S6R, R15A)-C_10_ led to a significant accumulation of UDP-MurNAc-pentapeptide indicating a lipid II mediated mode of action for both peptides (Fig. 4). The effect of the lipopeptides on bacterial cell morphology was further investigated using scanning electron microscopy. To this end, VRE 155 cells were imaged after exposure to either P8-d-(S6R, R15A)-C_10_ or nisin at 2× the MIC. A dramatic loss of structural integrity is observed for cells exposed to either P8-d-(S6R, R15A)-C_10_ or nisin with an indication of cell death *via* lysis (Fig. 5).

## Conclusions

In summary, we here describe the application of bicyclic peptide phage display for the *de novo* identification of novel lipid II binding scaffolds. Using both conventional and “mirror image” approaches a significantly enriched peptide sequence was found. Lipidation studies and alanine scanning yielded lipopeptides with antibacterial activity against a range of Gram-positive bacteria and little-to-no hemolytic activity. Most notable is the ability of these lipopeptides to effectively kill vancomycin-resistant pathogens as demonstrated with a panel of VRE isolates. Interestingly, the lipopeptides showed similar activity in both enantiomeric forms and both were confirmed to operate *via* a lipid II mediated mode of action. Future studies will be aimed at optimizing these lipopeptides to further enhance their antibacterial activity. Ongoing work is also aimed at applying bicyclic peptide phage display screens against other uniquely bacterial targets in pursuit of additional new-to-nature antibacterial agents.

## Conflicts of interest

There are no conflicts to declare.

## Supplementary Material

Supplementary information
