# Supplementary material for: De novo identification of lipid II binding lipopeptides with antibacterial activity against vancomycin-resistant bacteria
Source: Chem Sci. 2017 Oct 2;8(12):7991–7. doi: 10.1039/c7sc03413j (PMC5853558; doi:10.1039/c7sc03413j)
Supplement: Supplementary file 1 [file SC-008-C7SC03413J-s001.pdf]

Electronic Supplementary Material (ESI) for Chemical Science.
This journal is © The Royal Society of Chemistry 2017

Supporting Information for:

De novo identification of lipid II binding lipopeptides with antibacterial activity against vancomycin-resistant bacteria

*Peter 't Hart,[a] Thomas M. Wood,[a] Kamaleddin Haj Mohammad Ebrahim Tehrani,[a] Roel van Harten,[a] Małgorzata Śleszyńska,[a] Inmaculada Rentero Rebollo,[b] Antoni. P. A. Hendrickx,[c] Rob J. L. Willems,[c] Eefjan Breukink,[d] Nathaniel I. Martin[a,]**

[a]Chemical Biology & Drug Discovery Group, Utrecht Institute for Pharmaceutical Sciences, Utrecht University, Universiteitsweg 99, 3584CG Utrecht, The Netherlands, E-mail: n.i.martin@uu.nl
[b]Institute of Chemical Sciences and Engineering, Ecole Polytechnique Fédérale de Lausanne, Lausanne, Switzerland
[c]Department of Medical Microbiology, University Medical Center Utrecht, Utrecht, The Netherlands
[d]Biochemistry of Membranes Group, Bijvoet Center for Biomolecular Research, Utrecht University, The Netherlands

Table of Contents

1. Target Synthesis
1.1 General Procedures — Page S2
1.2 Overview of synthesis for compounds **1-14** — Page S3
1.3 Synthetic schemes, procedures, and analytical data — Page S3-S10
1.4 1D and 2D NMR spectra for all new compounds — Page S11-S25
1.5 2D NMR analysis of intermediates **8** and **14** and final compounds **1** and **2** — Page S26-31
1.6 HPLC analysis of intermediates **8** and **14** and final compounds **1** and **2** — Page S32-33

2. Phage Display
2.1 Phage selection — Page S34
2.2 Phage analysis — Page S34

3. Peptide synthesis
3.1 Non-lipidated peptides — Page S35
3.2 Lipidated peptides — Page S35-S36
3.3 HPLC data for all peptides — Page S37-S47
3.4 MS data for all peptides — Page S48

4. Biological and biochemical experiments
4.1 Minimum inhibitory concentration assays — Page S49
4.2 Hemolysis assays — Page S49
4.3 UDP-MurNAc-pentapeptide accumulation assay — Page S49
4.4 Antagonization assays — Page S49
4.5 Tables summarizing results of biological assays — Page S50-S51
4.6 Scanning Electron Microscopy — Page S51

Section 1. Target Synthesis

1.1 General Procedures

Reagents, solvents and solutions. All reagents employed were of American Chemical Society (ACS) grade or finer and were used without further purification unless otherwise stated. All reactions and fractions from column chromatography were monitored by thin layer chromatography (TLC) using plates with a UV fluorescent indicator (normal SiO_2, Merck 60 F254). One or more of the following methods were used for visualization: UV absorption by fluorescence quenching; iodine staining; phosphomolybdic acid:ceric sulfate:sulfuric acid:H_2O (10 g:1.25 g:12 mL:238 mL) staining; and ninhydrin staining. Flash chromatography was performed using Merck type 60, 230-400 mesh silica gel.

Instrumentation for Compound Characterization. NMR spectra were recorded at 400 or 500 MHz with chemical shifts reported in parts per million (ppm) downfield relative to tetramethylsilane (TMS). ^{1}H NMR data are reported in the following order: multiplicity (s, singlet; d, doublet; t, triplet; q, quartet; qn, quintet and m, multiplet), number of protons and coupling constant (J) in Hertz (Hz). When appropriate, the multiplicity is preceded by br, indicating that the signal was broad. ^{13}C NMR spectra were recorded at 100 or 125 MHz with chemical shifts reported relative to $CDCl_3$ δ 77.0. High-resolution mass spectrometry (HRMS) analysis was performed using an ESI-TOF instrument. All literature compounds had NMR spectra and mass spectra consistent with the assigned structures.

1.2 Synthesis of compounds **1-14**

The synthesis of the lipid I analogues was based largely on the total synthesis of lipid I and lipid II as described by VanNieuwenhze and coworkers.[1] The literature route was followed until obtaining the MurNAc pentapeptide intermediate **5** (see supplemental scheme 1). Next the required spacer was synthesized starting from 10-amino-1-decanol (see supplemental scheme 2). First the amine was protected with a Cbz-group to form compound **6**. Next, the alcohol was phosphorylated to yield compound **7** and the stage was set for the formation of the pyrophosphate. To do so, activation of one of the participating phosphates was required. Although previously described synthetic strategies typically chose to activate the MurNAc phosphate,[1,2] we chose to activate the spacer phosphate **7** instead. The easy to obtain spacer phosphate was far less valuable compared to the other phosphate precursor **5** and multiple equivalents of **7** could easily be added to drive the reaction to completion. After formation of pyrophosphate intermediate **8** it was purified by preparative HPLC and ready for biotinylation and final deprotection (see supplemental scheme 3). First the Cbz-group was removed under standard hydrogenation conditions followed by coupling the biotin as activated NHS-ester. Glycine was added to scavenge the excess NHS-ester followed by addition of a small excess of NaOH to perform a global deprotection to yield **Target 1** (compound **1**). Preparative HPLC purification gave the final compound in 53% yield over the final three steps.

The same route was used for preparing the enantiomeric **Target 2** (compound **2**) from the corresponding enantiomeric building blocks. All enantiomeric building blocks were commercially available with the exception of *N*-acetyl-L-glucosamine (**10**) which was synthesized starting from L-arabinose (see supplemental scheme 4).

1.3 Synthetic schemes

Supplemental scheme 1: Synthesis of pyrophosphate intermediate **8**

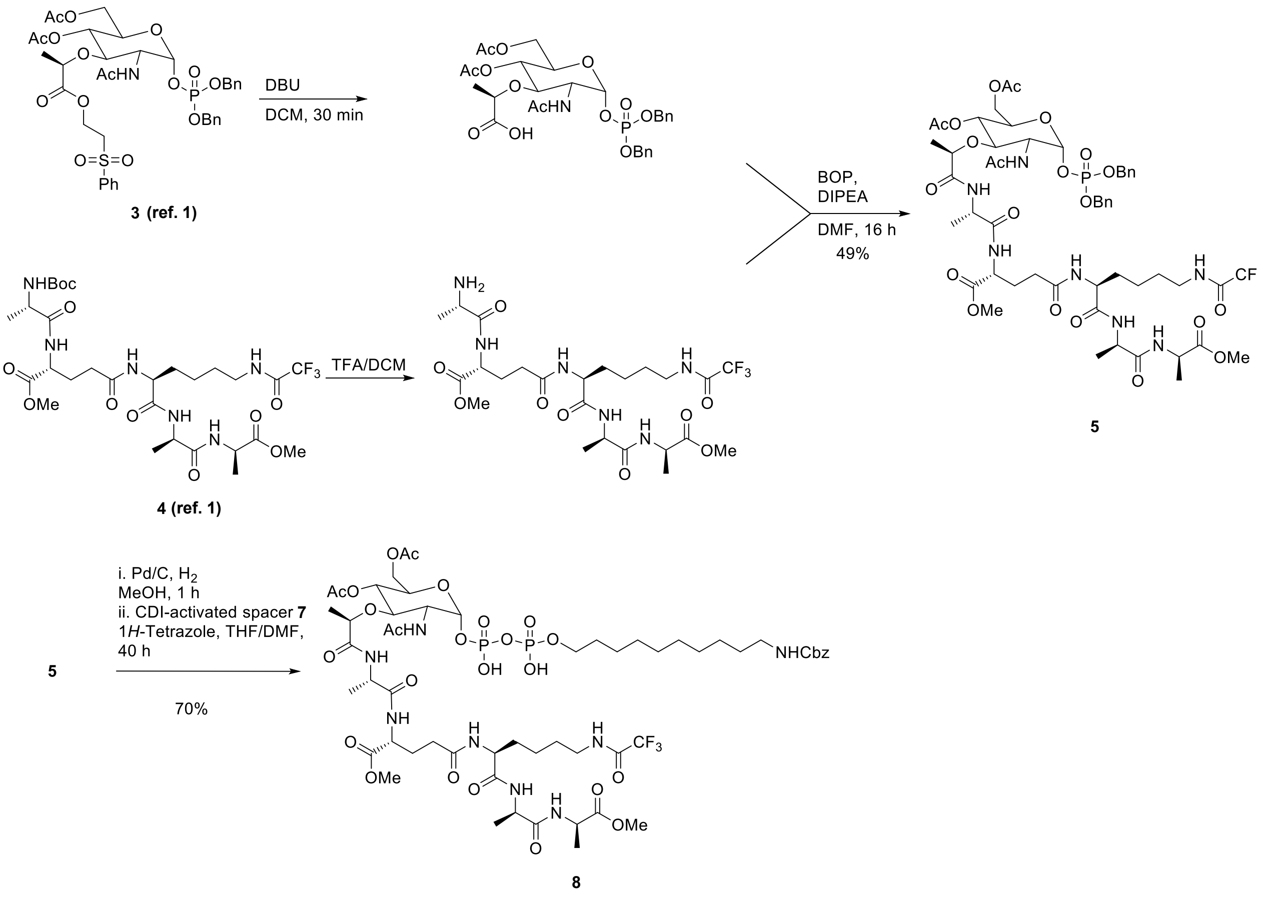

Supplemental scheme 2: synthesis of spacer **7**

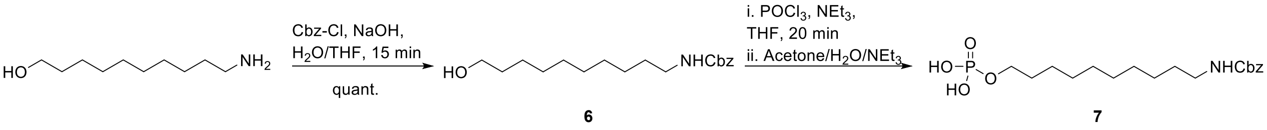

Supplemental scheme 3: Synthesis of Target 1 (compound **1**)

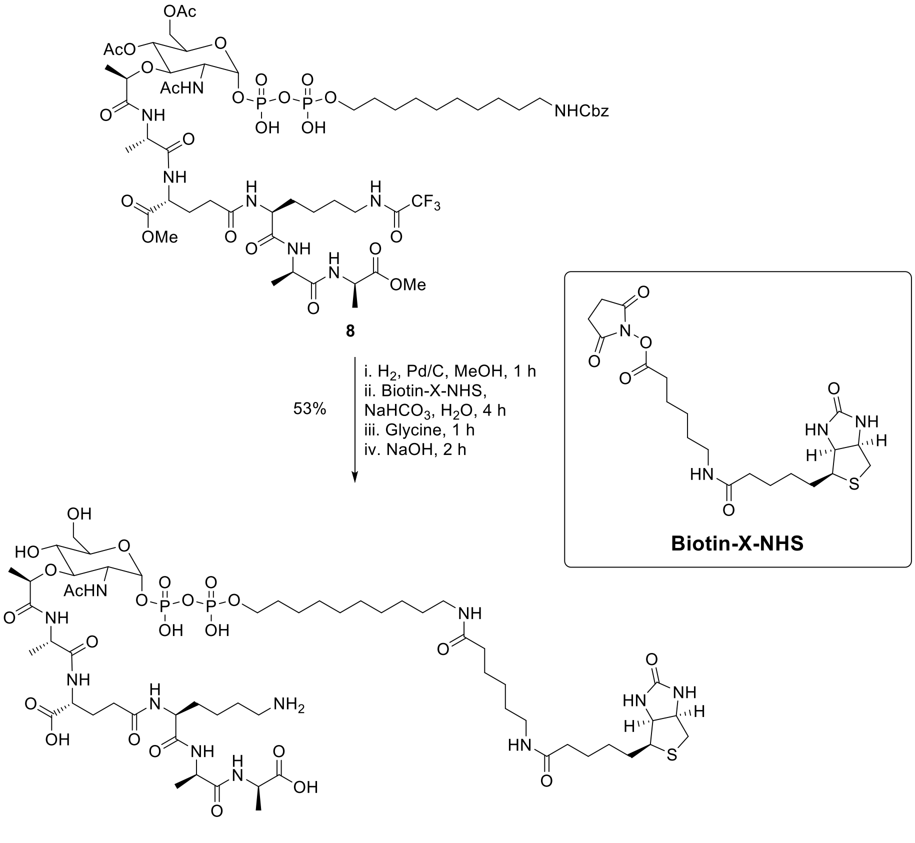

Target 1 (Compound **1**)

Supplemental scheme 4: Synthesis of *N*-acetyl-L-glucosamine (compound **10**)

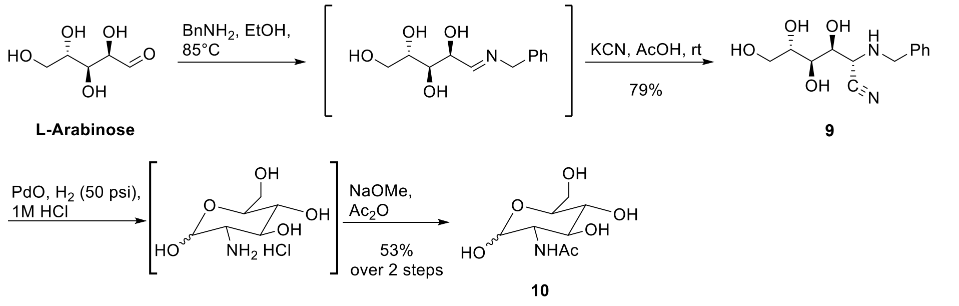

Supplemental scheme 5: synthesis of enantiomeric intermediates **11** and **12**

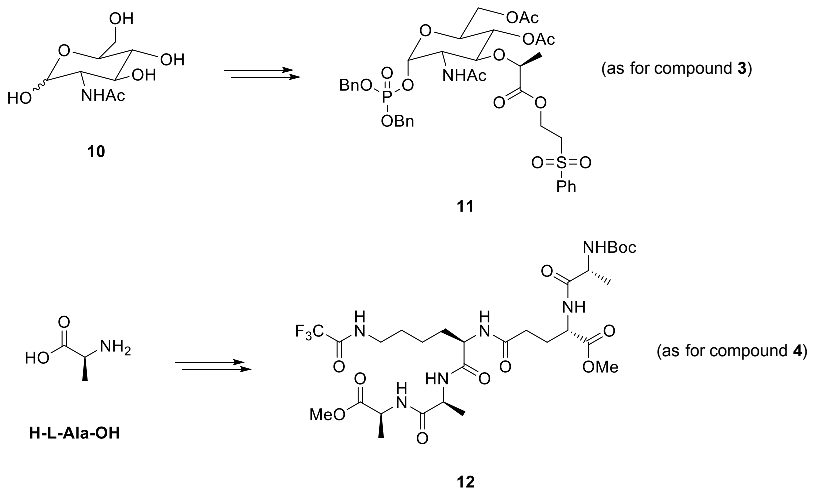

Supplemental scheme 6: Synthesis of enantiomeric pyrophosphate intermediate **14**

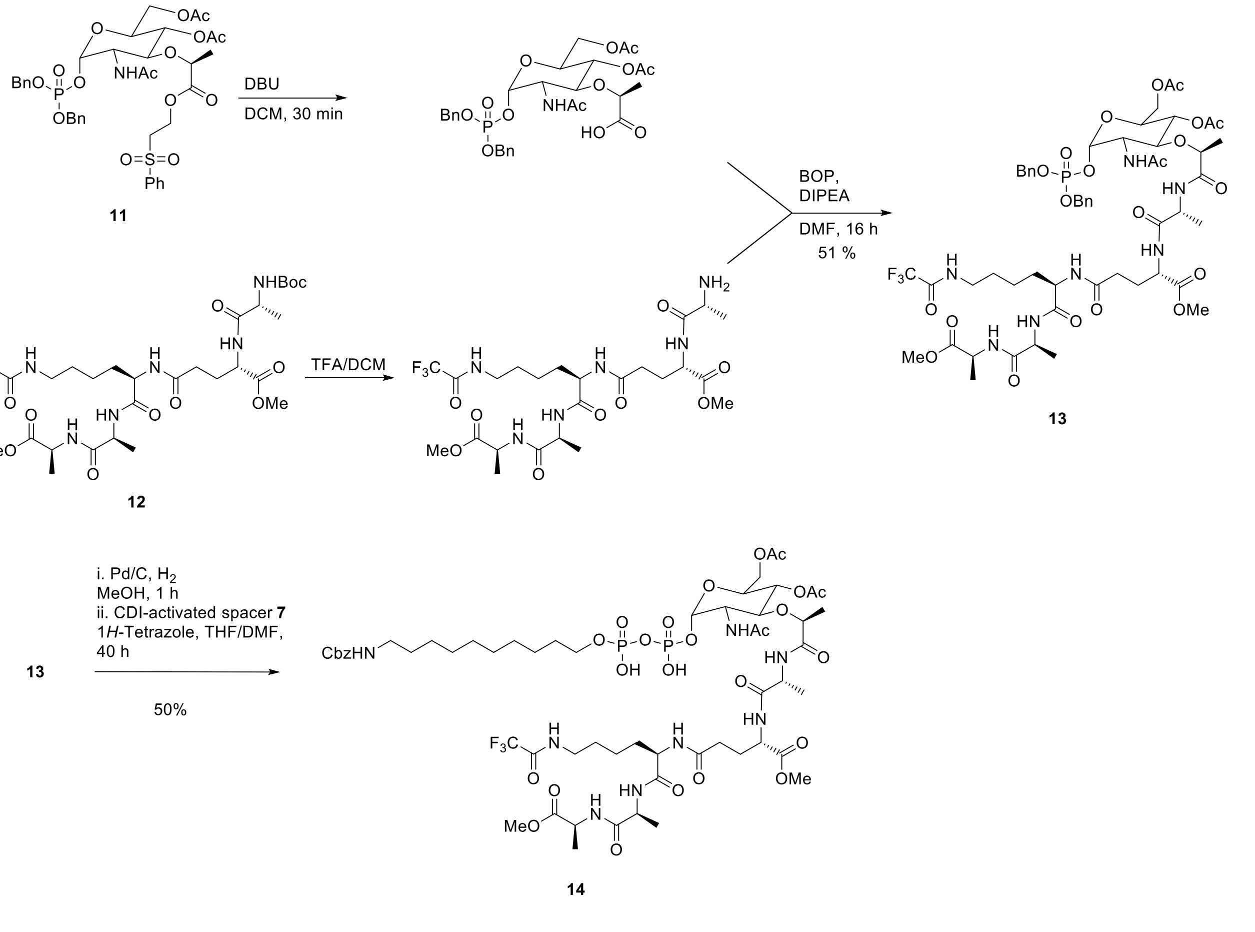

Supplemental scheme 7: Synthesis of Target 2 (compound **2**)

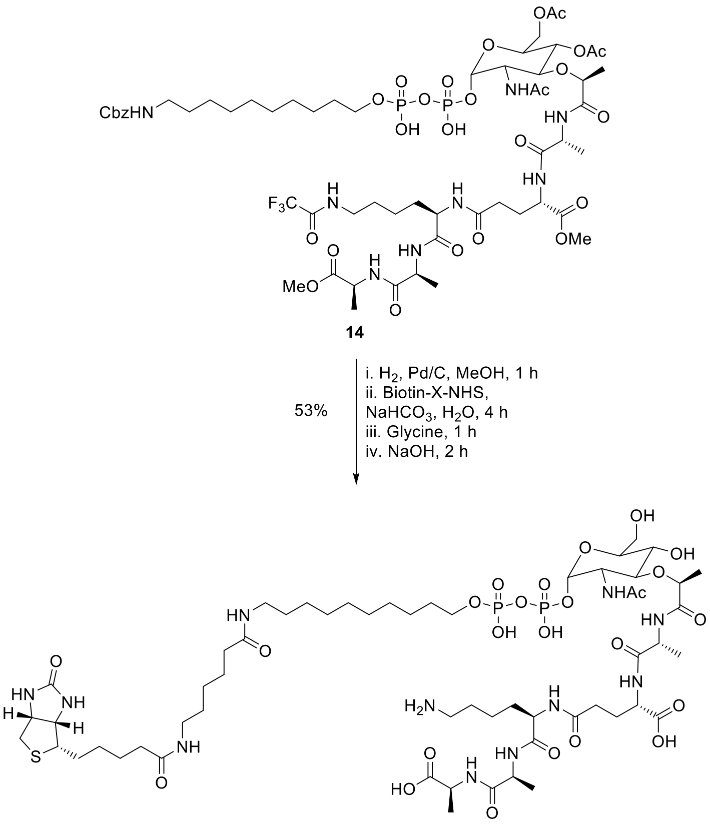

Target 2 (Compound **2**)

1.3.1 Synthetic procedures and analytical data for compounds **1-14**

2-(Phenylsulfonyl)ethyl (R)-2-(((2R,3R,4R,5S,6R)-3-acetamido-5-acetoxy-6-(acetoxymethyl)-2-((bis(benzyloxy)phosphoryl)oxy)tetrahydro-2H-pyran-4-yl)oxy)propanoate (3)

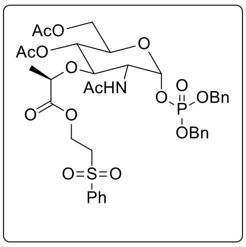

Synthesis as described by VanNieuwenhze et al.[1] $[\alpha]_D$ = +60.3 (c 0.4, MeOH). Analytical data corresponds with literature values.[1]

Methyl N2-((tert-butoxycarbonyl)-L-alanyl)-N5-((S)-1-(((R)-1-(((R)-1-methoxy-1-oxopropan-2-yl)amino)-1-oxopropan-2-yl)amino)-1-oxo-6-(2,2,2-trifluoroacetamido)hexan-2-yl)-D-glutaminate (4)

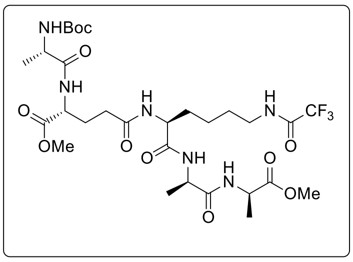

Synthesis as described by Eid et al.[4] Synthesis as for compound **4** $[\alpha]_D$ = +20.2 (c 0.2, MeOH). Analytical data corresponds with literature values.[4]

Protected MurNAc-pentapeptide-phosphate (5)

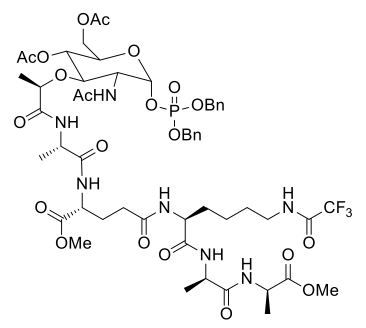

5

Synthesis as described by VanNieuwenhze et al.[1] Analytical data corresponds with literature values.[1]

Benzyl (10-hydroxydecyl)carbamate (6)

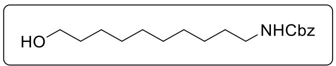

Synthesis adapted from a method described by Delcros et al.[3] NaOH (127.2 mg, 3.18 mmol) was dissolved in H_2O (10 ml) and 10-amino-1-decanol (500 mg, 2.89 mmol) was added. THF (10 ml) was added to completely dissolve everything before benzylchloroformate (454 µl, 3.18 mmol) was added dropwise. After 15 min the formed precipitate was filtered off and washed with water. The product was dried under high vacuum. Yield: 876 mg (2.85 mmol; 99%). R_f: 0.5 (EtOAc/Hex 1:1) ^{1}H NMR (300 MHz, $CDCl_3$) δ 7.39 – 7.28 (m, 5H), 5.09 (s, 2H), 4.71 (bs, 1H), 3.63 (q, *J* = 6.0 Hz, 2H), 3.18 (q, *J* = 6.0 Hz, 2H), 1.53 (m, 4H), 1.28 (s, 12H). ^{13}C NMR (101 MHz, $CDCl_3$) δ 156.5, 136.8, 128.6 (x2), 128.2, 128.2, 77.5, 77.2, 76.8, 66.7, 63.1, 41.2, 32.9, 30.1, 29.6, 29.5, 29.5, 29.3, 26.8, 25.8. HRMS ESI-TOF m/z calcd for $C_{18}H_{29}NO_3$ ($[M+H]^+$): 308.2226, Found 308.2252

Benzyl (10-(phosphonooxy)decyl)carbamate (7)

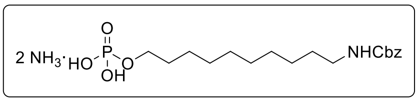

$POCl_3$ (466 µl, 5 mmol) was dissolved in THF (10ml) and NEt_3 was added and stirred for 5 min before compound **6** (307.4 mg, 1 mmol) was added as a solution in 5 ml THF. After stirring for 20 min the reaction mixture is poured into acetone/H_2O/NEt_3 (88:10:2, 50 ml) and stirred for 1h. Organic solvents were removed under reduced pressure and an aqueous suspension was obtained. The solid material was filtered, washed with H_2O and dissolved in 25% NH_4OH (50 ml). The aqueous solution was extracted with DCM (50 ml) before it was evaporated to dryness to obtain the product as a white powder. Yield: 300.6 mg (0.71 mmol; 71%) ^{1}H NMR (300 MHz, DMSO-d6) δ 7.39 – 7.11 (m, 5H), 6.55 (s, 3H), 5.08 (s, 2H), 3.97 (q, *J* = 6.0 Hz, 2H), 3.15 (q, *J* = 6.1 Hz, 2H), 1.69-1.55 (m, 2H), 1.54 – 1.41 (m, 2H), 1.26 (s, 12H). ^{13}C NMR (101 MHz, DMSO-d6) δ 156.1, 137.3, 128.3, 127.7, 127.7, 65.0, 64.5, 64.4, 45.3, 30.2, 30.1, 29.4, 29.0, 28.8, 26.3, 25.3, 8.4. HRMS ESI-TOF m/z calcd for $C_{18}H_{30}NO_6P$ ($[M-H]^-$): 386.1733, Found 386.1784

Cbz-protected pyrophosphate intermediate (8)

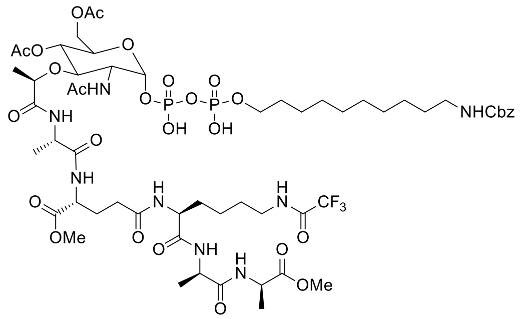

8

Compound **5** (25 mg, 20.3 µmol) was dissolved in 5 ml MeOH and Pd/C (50 mg) was added. The mixture was stirred under H_2 atmosphere and after 45 min TLC (10% MeOH in DCM) indicated that the starting material was consumed. The catalyst was removed by filtration over celite and the solvents were evaporated. The deprotected compound was evaporated from toluene 5 times. Compound **7** (15.7 mg, 40.6 µmol) was dried by coevaporation from toluene (5x) and suspended in 5 ml dry THF and CDI (32.9 mg, 203 µmol) was added. The reaction was stirred for 2h under Ar (g). To quench the excess CDI, dry MeOH (1.5 ml) was added and the mixture was stirred for another 15 min. After evaporation of solvents the activated spacer was coevaporated from toluene 5 times. Deprotected compound **5** was added

and the mixture was coevaporated from toluene 5 times. The dry solids were dissolved in 0.5 ml dry DMF and 1 ml dry THF, followed by addition of 1*H*-Tetrazole (3 mg, 42.8 µmol). The mixture was stirred for 16 h when HPLC analysis indicated compound **5** was still present. More compound **7** (23.6 mg, 61 µmol) was activated with CDI (49.4 mg, 304.8 µmol) and added to the reaction as a solution in 0.75 ml dry THF. After another 24 h all starting material was consumed and the product was purified by preparative HPLC (Dr.Maisch ReproSil-Pur 120 C18-AQ, 10 µm, 250 x 22 mm) using a gradient of 50-100% buffer B over 65 min at a flow rate of 8.4 ml/min. Buffer A: 50 mM NH_4HCO_3, Buffer B: MeOH. Fractions containing pure product were pooled and lyophilized. Yield: 20.7 mg (14.2 µmol; 70 %). $[\alpha]_D$ = +35.9 ± 1.0 (c 0.28, MeOH/H_2O 1:1) HRMS ESI-TOF m/z calcd for $C_{57}H_{89}F_3N_8O_{26}P_2$ ($[M-H]^-$): 1419.5237, Found 1419.5258. NMR analysis is discussed in section 1.5.

Target 1 (compound **1**)

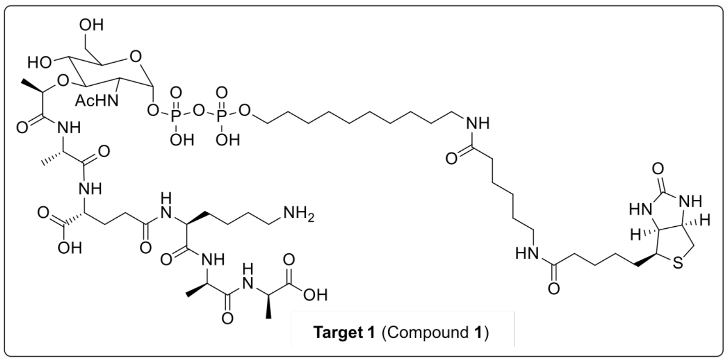

Target 1 (Compound **1**)

Compound **8** (26.5 mg, 18.6 µmol) was dissolved in MeOH (2.5 ml) and Pd/C was added as a suspension in MeOH (2.5 ml). The mixture was stirred under a hydrogen atmosphere for 2 h. The catalyst was removed by filtration over celite and the filtrate was evaporated to dryness. The deprotected product was dissolved in H_2O/dioxane (1:1, 2 ml) and $NaHCO_3$ (3.13 mg, 37.2 µmol) was added followed by biotin-X-NHS (10.8 mg, 22.4 µmol). The mixture was stirred until all starting material had been consumed (as analyzed by HPLC). Glycine (1.4 mg, 18.6 µmol) was added to quench the excess biotin-X-NHS and the mixture was stirred for 1h. After addition of 1M NaOH (186 µl) the mixture was stirred for another 2 h before the dioxane was removed by evaporation under vacuum. The product was immediately purified by preparative HPLC (Dr.Maisch ReproSil-Pur 120 C18-AQ, 10 µm, 250 x 22 mm) using a gradient of 30-100% buffer B over 105 min at a flow rate of 8.4 ml/min. Buffer A: 50 mM NH_4HCO_3, Buffer B: MeOH. Fractions containing product were pooled and lyophilized. Yield: 14.7 mg (9.9 µmol, 53%). HRMS ESI-TOF m/z calcd for $C_{57}H_{101}N_{11}O_{24}P_2S$ ($[M-H]^-$): 1416.6139, Found 1416.6169. NMR analysis is discussed in section 1.5.

(2R,3S,4R,5S)-2-(benzylamino)-3,4,5,6-tetrahydroxyhexanenitrile (9)

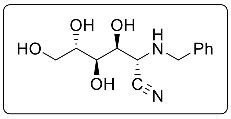

Method adapted from Kuhn et al.[5] L-Arabinose (10 g, 66.6 mmol) was suspended in dry EtOH (40 ml) and benzylamine (10 ml, 91.6 mmol) was added. The mixture was heated to 85°C until everything dissolved. After cooling to 30°C KCN (4.76 g, 73.1 mmol) was added and the flask was sealed with a septum. AcOH (5.78 ml, 10.1 mmol) was added while vigorously stirring the suspension until it became homogenous. The solution was stirred at r.t. for 30 min before a precipitate starts to form. The mixture is cooled on ice for 1h and the solids are collected by filtration and washed with cold EtOH. Yield: 13.92 g (52.3 mmol, 79%) R_f: 0.76 (MeOH/n-BuOH/H_2O/conc. Ammonia 80:20:10:3) ^1H NMR (400 MHz, DMSO-d6) δ 7.41 – 7.21 (m, 5H), 4.97 (d, *J* = 6.0 Hz, 1H), 4.69 (d, *J* = 5.9 Hz, 1H), 4.55 (d, *J* = 4.2 Hz, 1H), 4.36 (t, *J* = 5.4 Hz, 1H), 4.02 – 3.87 (m, 2H), 3.79 – 3.66 (m, 2H), 3.64 – 3.54 (m, 1H), 3.48 (d, *J* = 2.6 Hz, 2H), 3.41 (dd, *J* = 10.3, 4.9 Hz, 1H), 2.94 (q, *J* = 6.2 Hz, 1H). ^{13}C NMR (101 MHz, DMSO-d6) δ 139.2, 128.3, 128.1, 127.0, 119.5, 71.0, 70.8, 69.4, 63.3, 52.7, 50.8. HRMS ESI-TOF m/z calcd for $C_{13}H_{18}N_2O_4$ ($[M+Na]^+$): 289.1164, Found 289.1208

L-glucosamine hydrochloride

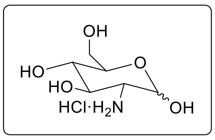

Method adapted from Kuhn et al.[5] Compound **8** (13.92 g, 52.3 mmol) was dissolved in 1M HCl (125 ml) and Pd(II)O hydrate (70-80% Pd) was added. The mixture was hydrogenated at 50 PSI for 24 h when TLC indicated full consumption of the starting material. The catalyst was removed by filtration over

celite and the aqueous solution was evaporated to dryness. A yellowish powder was obtained and dissolved in EtOH at 60°C. After cooling to 0°C the product was obtained by filtration as an off-white powder. The product was used without further purification. Yield: 7.42 g (34.4 mmol; 66%) R_f: 0.37 (MeOH/n-BuOH/H_2O/conc. Ammonia 80:20:10:3)

_N_-acetyl-L-glucosamine (10)

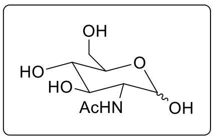

Method described by Berger et al.[6] Freshly cut sodium (0.95 g, 41.3 mmol) was dissolved in dry MeOH (75 ml) and added to a suspension of compound **9** (7.42 g, 34.4 mmol). The mixture was stirred for 30 min before Ac_2O (8.1 ml, 86 mmol) was added and left to stir overnight. The mixture was cooled to -20°C and allowed to precipitate for 24 h. The precipitate was collected by filtration and washed with Et_2O and dried under vacuum. Yield: 6.13 g, (27.7 mmol, 53% over 2 step (starting from **8**)) R_f: 0.6 (MeOH/n-BuOH/H_2O/conc. Ammonia 80:20:10:3). ^{1}H NMR (400 MHz, DMSO-d6) δ 7.70 (d, *J* = 7.1 Hz, 1H), 6.40 (d, *J* = 4.4 Hz, 1H), 4.99 (d, *J* = 5.0 Hz, 1H), 4.92 (dd, *J* = 4.2, 2.4 Hz, 1H), 4.76 (d, *J* = 3.7 Hz, 1H), 4.45 (t, *J* = 5.8 Hz, 1H), 3.64 – 3.42 (m, 5H), 3.19 – 3.03 (m, 2H), 1.82 (s, 3H). ^{13}C NMR (101 MHz, DMSO-d6) δ 169.4, 90.6, 72.1, 71.2, 70.5, 61.2, 54.3, 22.7. NMR corresponds with commercial *N*-acetyl-D-glucosamine. HRMS ESI-TOF m/z calcd for $C_8H_{15}NO_6$ ($[M+H]^+$): 222.0978, Found 222.1000

2-(phenylsulfonyl)ethyl (S)-2-(((2S,3S,4S,5R,6S)-3-acetamido-5-acetoxy-6-(acetoxymethyl)-2-((bis(benzyloxy)phosphoryl)-oxy)tetrahydro-2H-pyran-4-yl)oxy)propanoate (11)

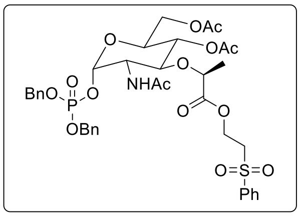

Synthesis as for compound **3** $[\alpha]_D$ = -62.6 (c 0.4, MeOH). Analytical data corresponds with literature values.[1]

Methyl N2-((tert-butoxycarbonyl)-D-alanyl)-N5-((R)-1-(((S)-1-(((S)-1-methoxy-1-oxopropan-2-yl)amino)-1-oxopropan-2-yl)amino)-1-oxo-6-(2,2,2-trifluoro-acetamido)hexan-2-yl)-L-glutaminate (12)

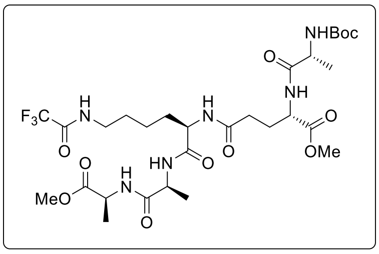

Synthesis as described by Eid et al.[4] Synthesis as for compound **4** $[\alpha]_D$ = -20.8 (c 0.2, MeOH). Analytical data corresponds with literature values.[4]

Phosphomuramic acid pentapeptide (13)

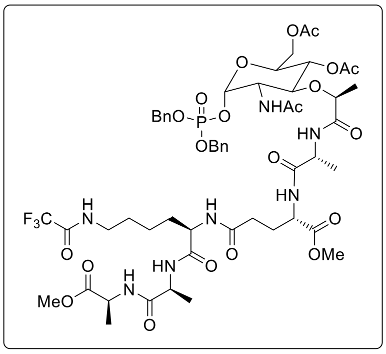

Synthesis as for compound **5**. Analytical data corresponds with literature values.[1]

Pyrophosphate intermediate (14)

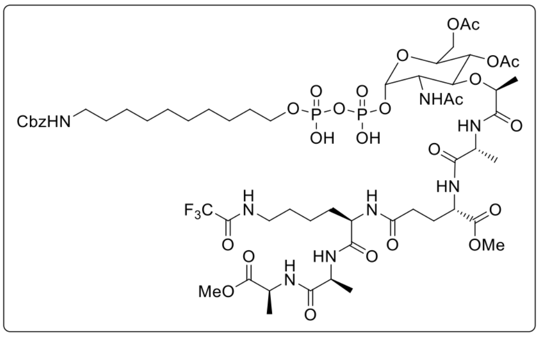

Synthesis as for compound **8**: Yield: 26.5 mg (18.2 µmol; 50%). $[\alpha]_D$ = -33.9 ± 1.6 (c 0.29, MeOH/H_2O 1:1) HRMS ESI-TOF m/z calcd for $C_{57}H_{89}F_3N_8O_{26}P_2$ ($[M-2H]^{2-}$): 709.2580, Found: 709.2575. For NMR analysis see section 1.5.

Target 2 (compound **2**)

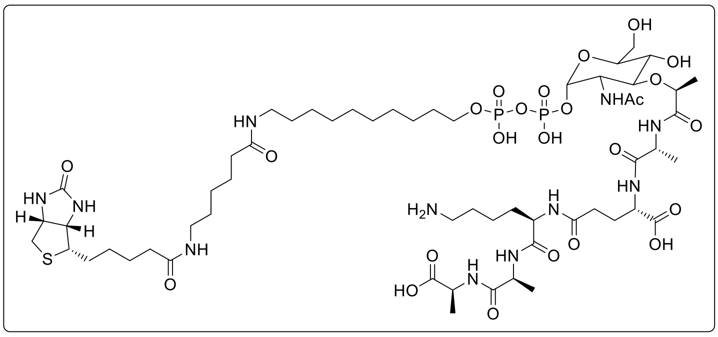

Synthesis as for compound **1**: Yield: 14.7 mg (9.87 µmol; 53%) HRMS ESI-TOF m/z calcd for $C_{57}H_{101}N_{11}O_{24}P_2S$ ($[M-H]^{-}$): 1416.6139, Found 1416.6187. For NMR analysis see section 1.5.

References

[1] M. S. VanNieuwenhze, S. C. Mauldin, M. Zia-Ebrahimi, J. A. Aikins, L. C. Blaszczak, *J. Am. Chem. Soc.* **2001**, *123*, 6983–6988.

[2] B. Schwartz, J. A. Markwalder, Y. Wang, *J. Am. Chem. Soc.* **2001**, *123*, 11638–11643.

[3] J. G. Delcros, S. Tomasi, S. Carrington, B. Martin, J. Renault, I. S. Blagbrough, P. Uriac, *J. Med. Chem.* **2002**, *45*, 5098–5111.

[4] C. N. Eid, M. J. Nesler, M. Zia-ebrahimi, C. E. Wu, R. Yao, K. Cox, J. Richardson, *J. Label. Compd. Radiopharm.* **1998**, *41*, 705–716.

[5] R. Kuhn, W. Kirschenlohr, *Angew. Chemie* **1955**, *67*, 786.

[6] I. Berger, A. a Nazarov, C. G. Hartinger, M. Groessl, S.-M. Valiahdi, M. a Jakupec, B. K. Keppler, *ChemMedChem* **2007**, *2*, 505–14.

[7] I. Rentero Rebollo, C. Heinis, *Methods* **2013**, *60*, 46–54.

[8] I. Rentero Rebollo, M. Sabisz, V. Baeriswyl, C. Heinis, *Nucleic Acids Res.* **2014**, *42*, e169–e169.

1.4 1D and 2D NMR spectra for new compounds

Compound **6**. ^{1}H NMR ($CDCl_3$)

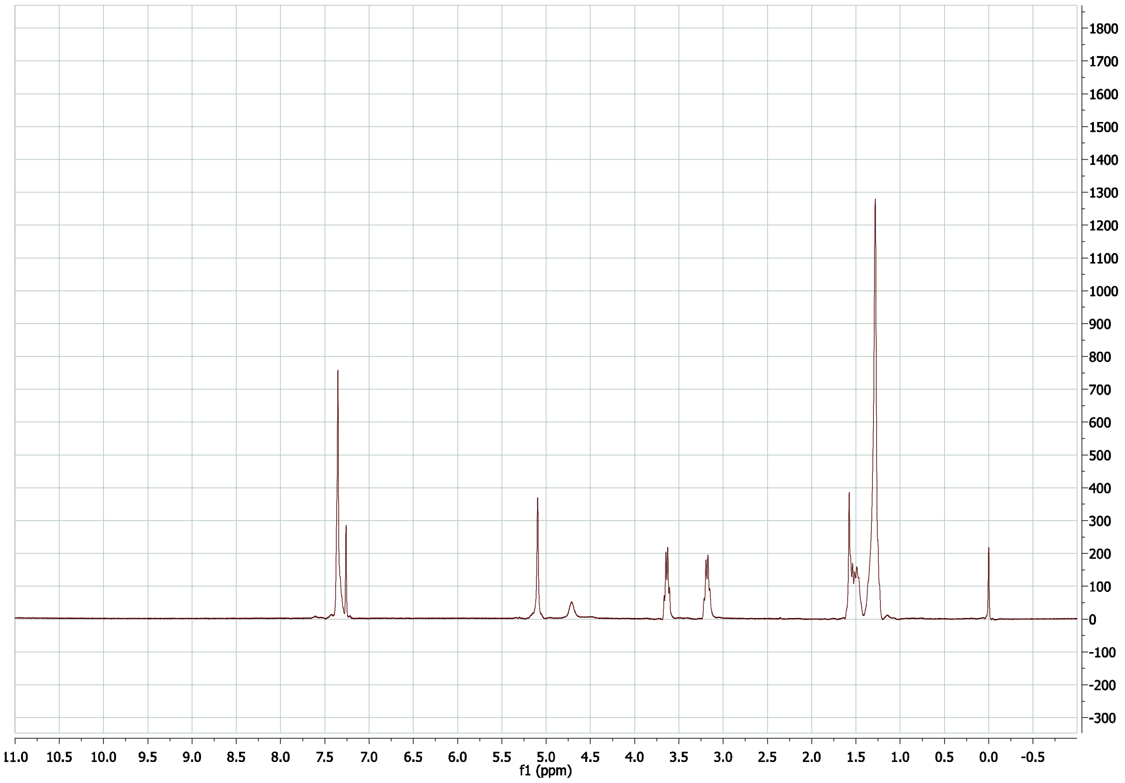

Compound **6**. ^{13}C APT ($CDCl_3$)

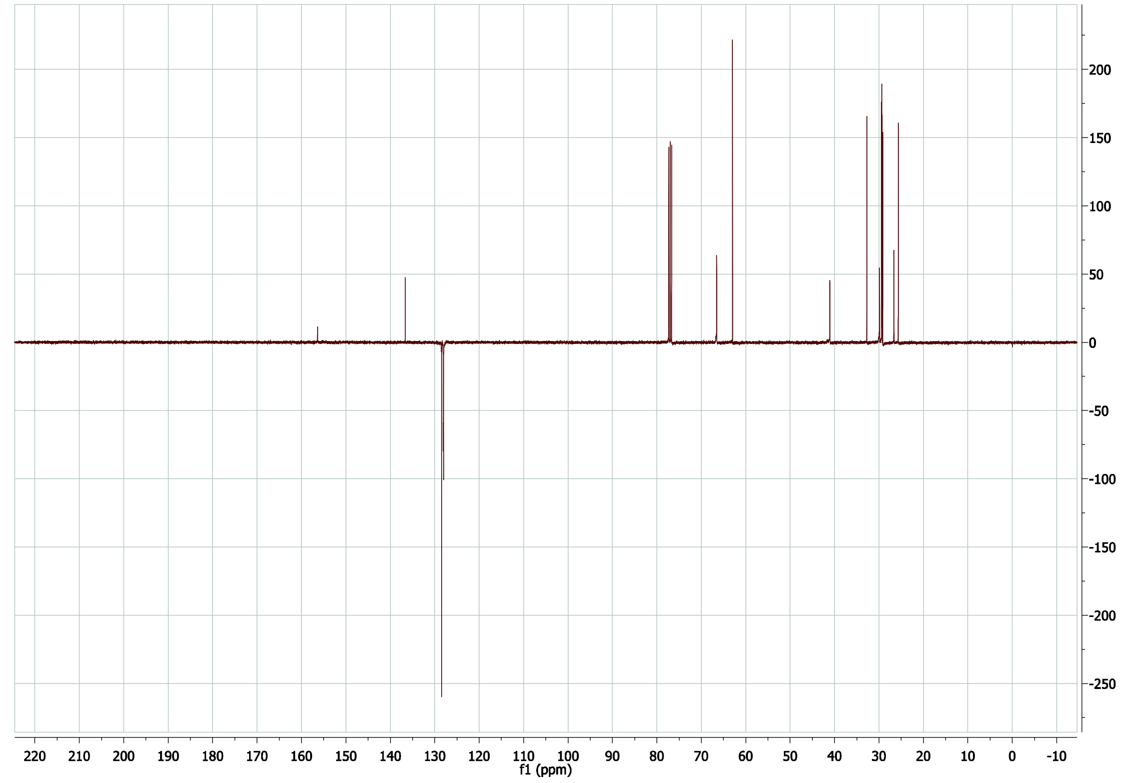

Compound **7**. ^{1}H NMR (DMSO-d6)

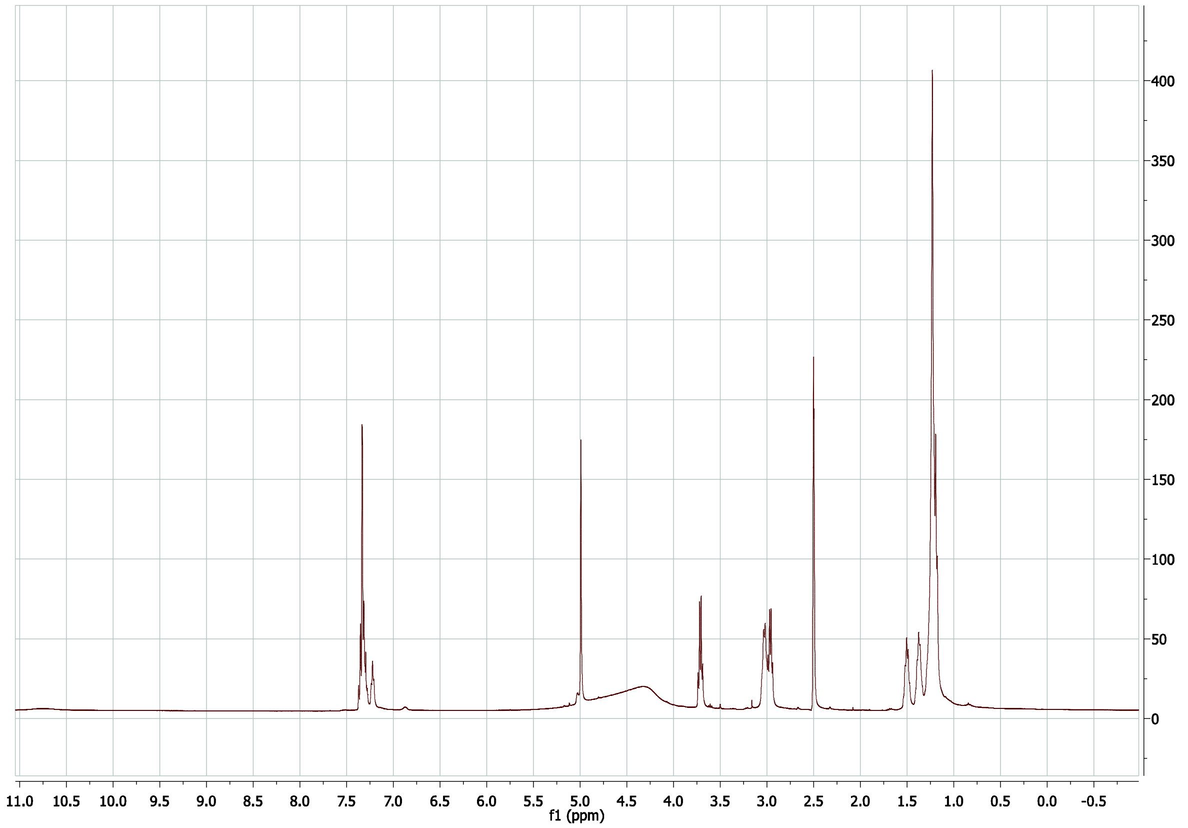

Compound **7**. ^{13}C APT (DMSO-d6)

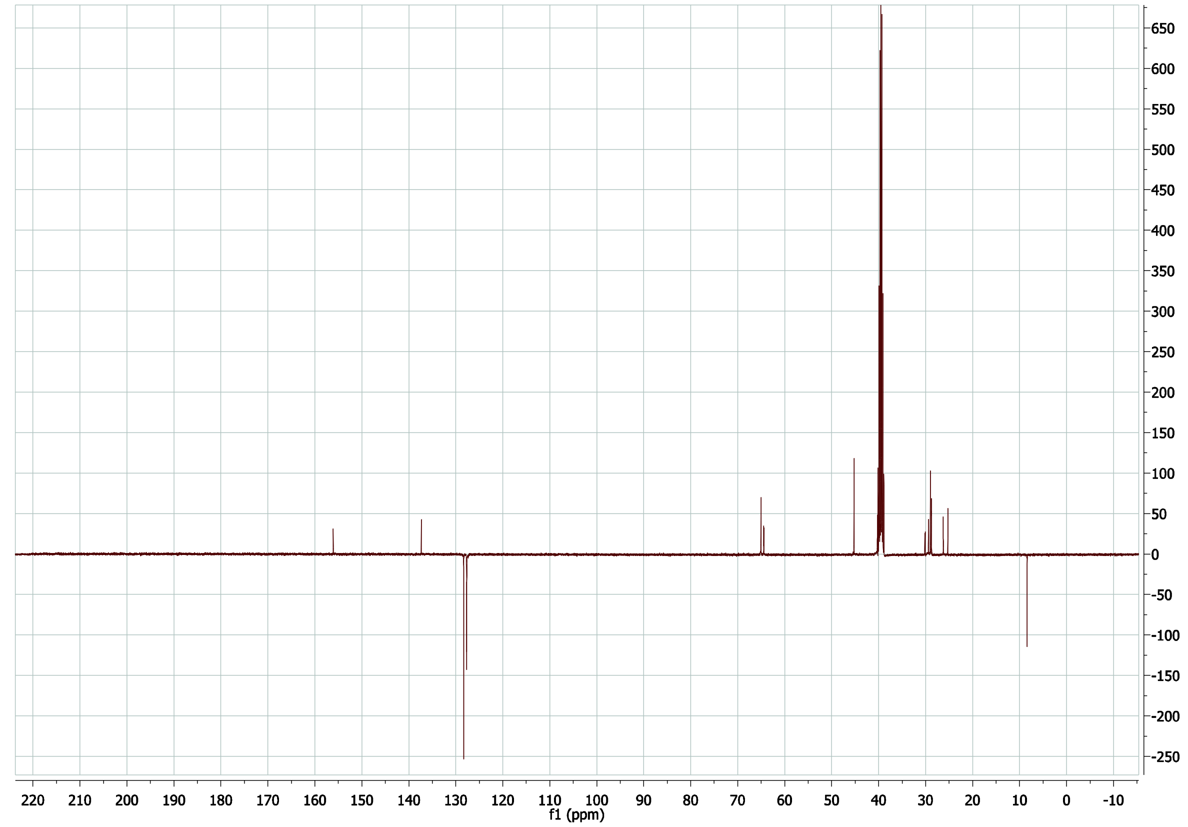

Compound **7**. ^{31}P NMR (DMSO-d6)

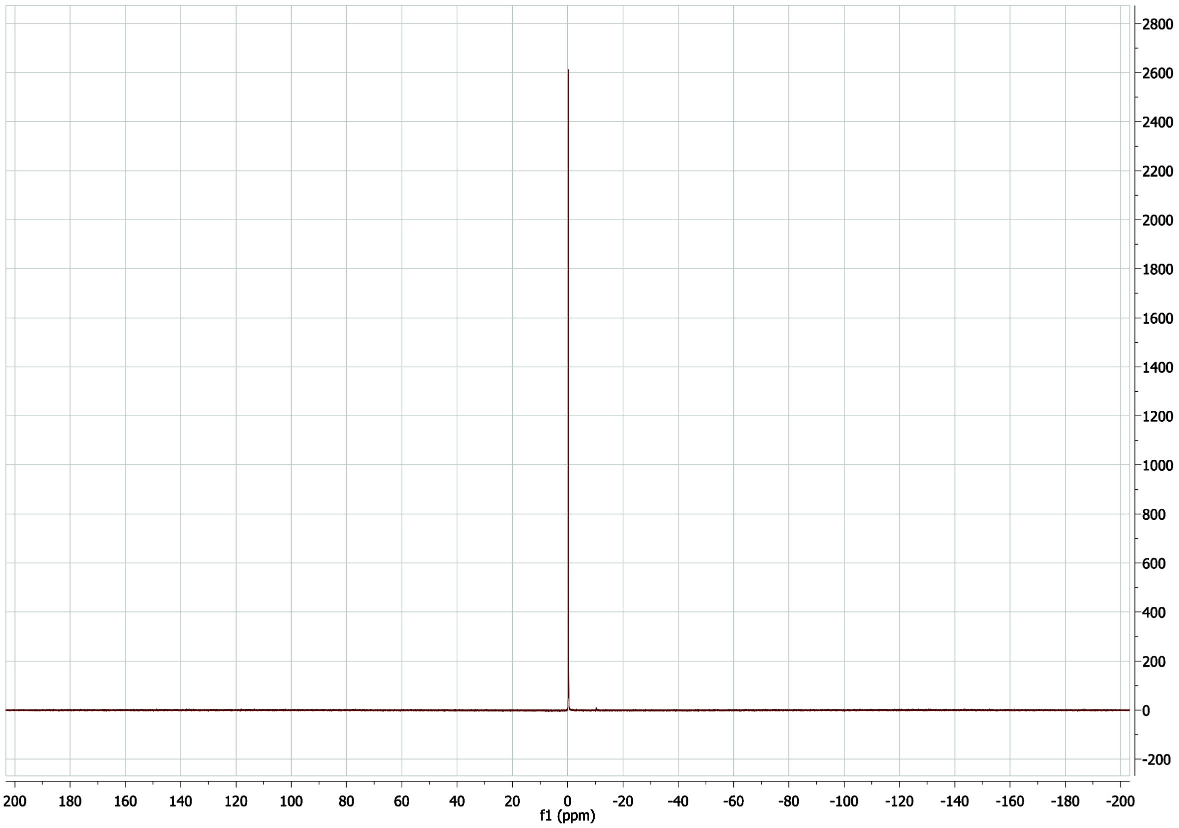

Compound **8** ^{13}C HSQC (CD_3OD)

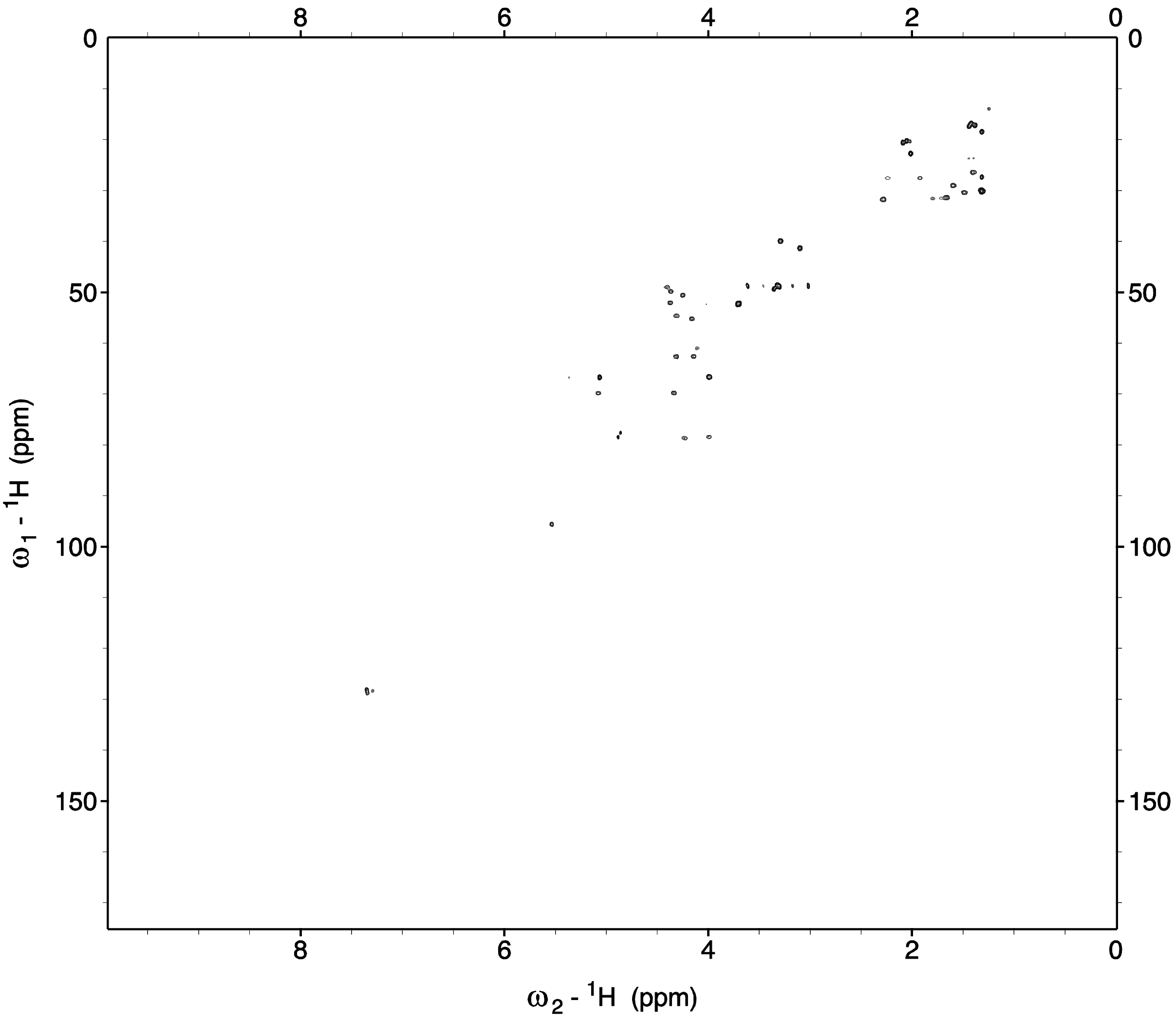

Compound **8** TOCSY (CD_3OD)

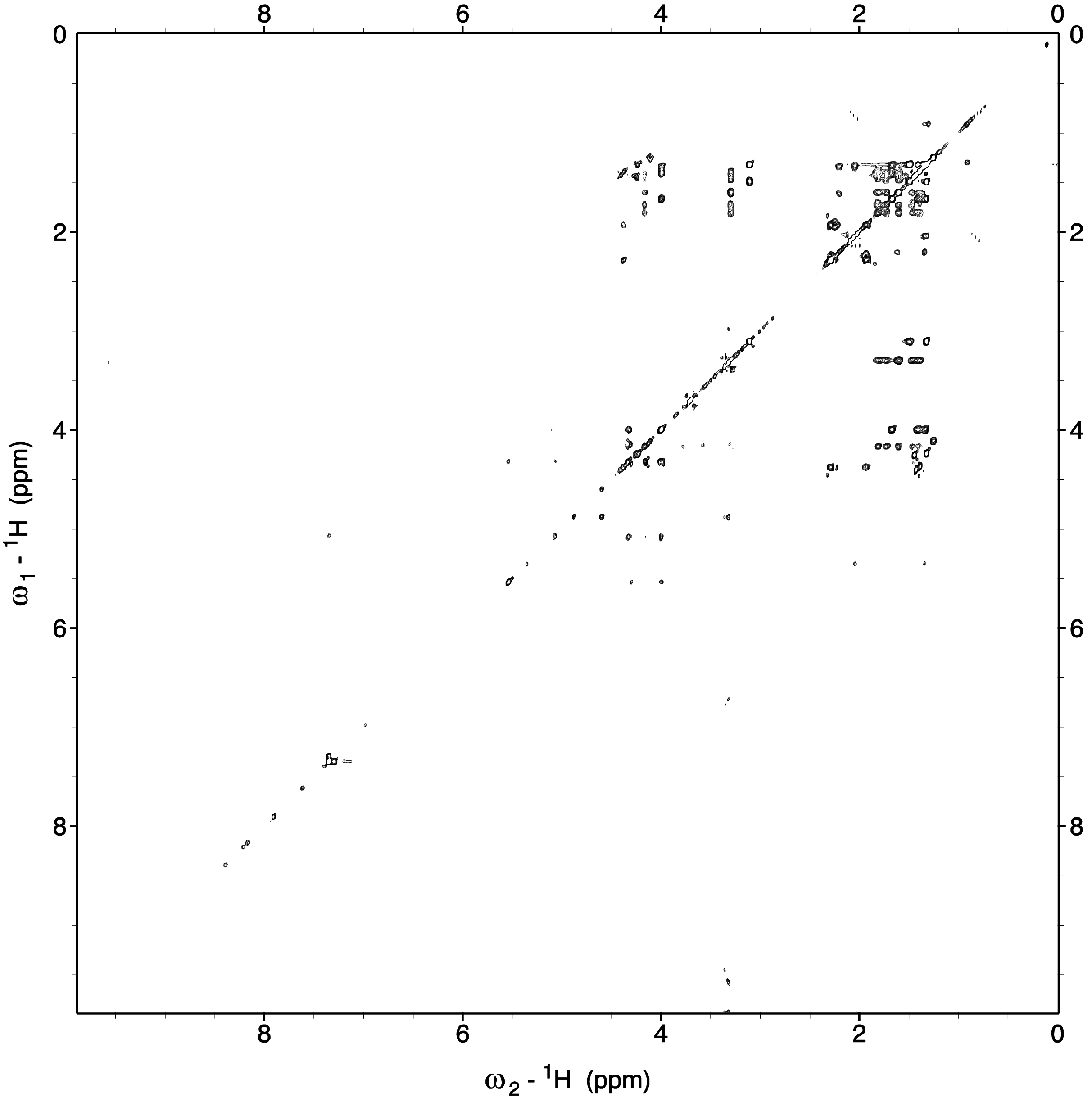

Compound **1** ^{13}C HSQC (CD_3OD)

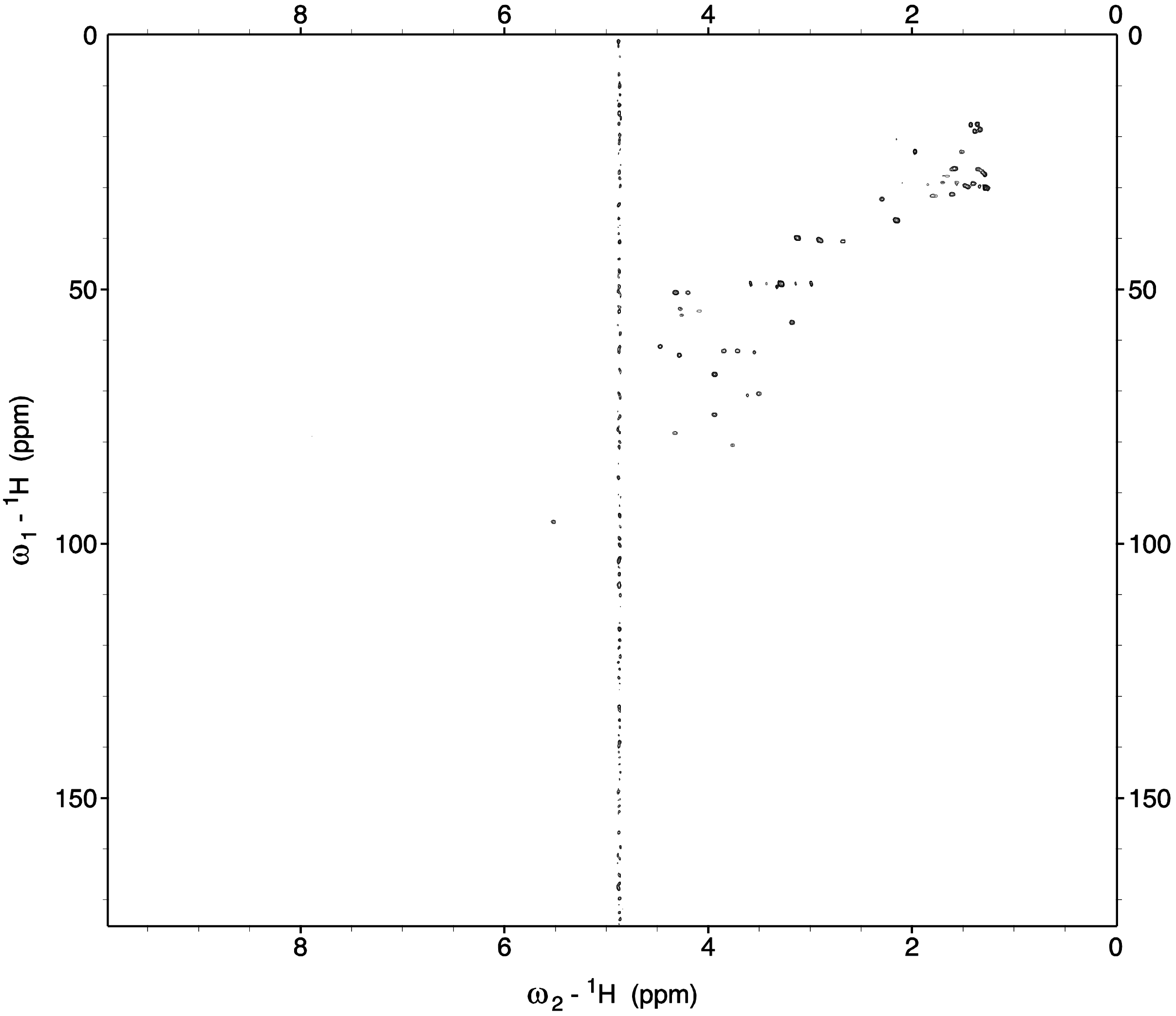

Compound **1** TOCSY (CD_3OD)

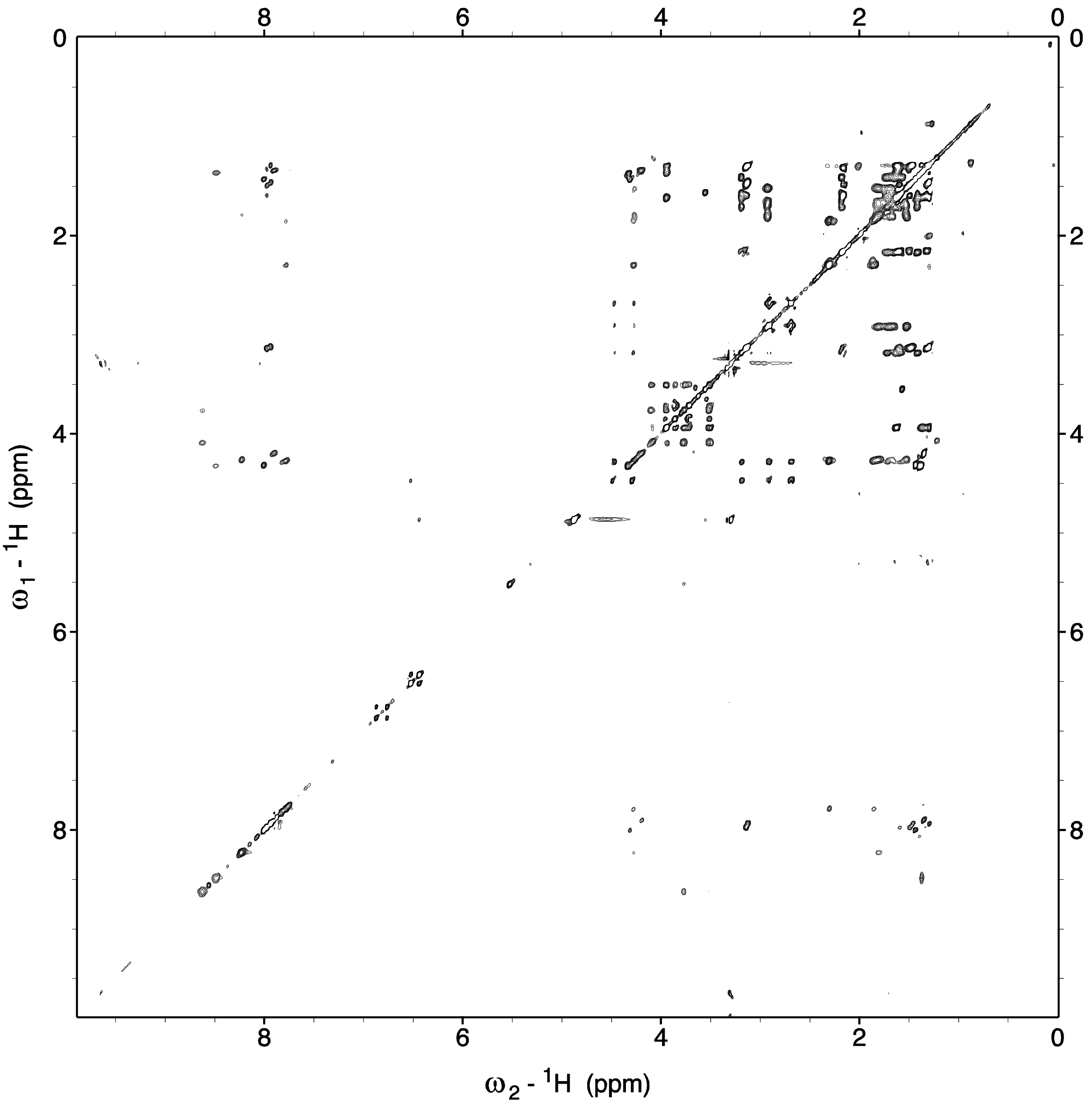

Compound **9**. ^{1}H NMR (DMSO-d6)

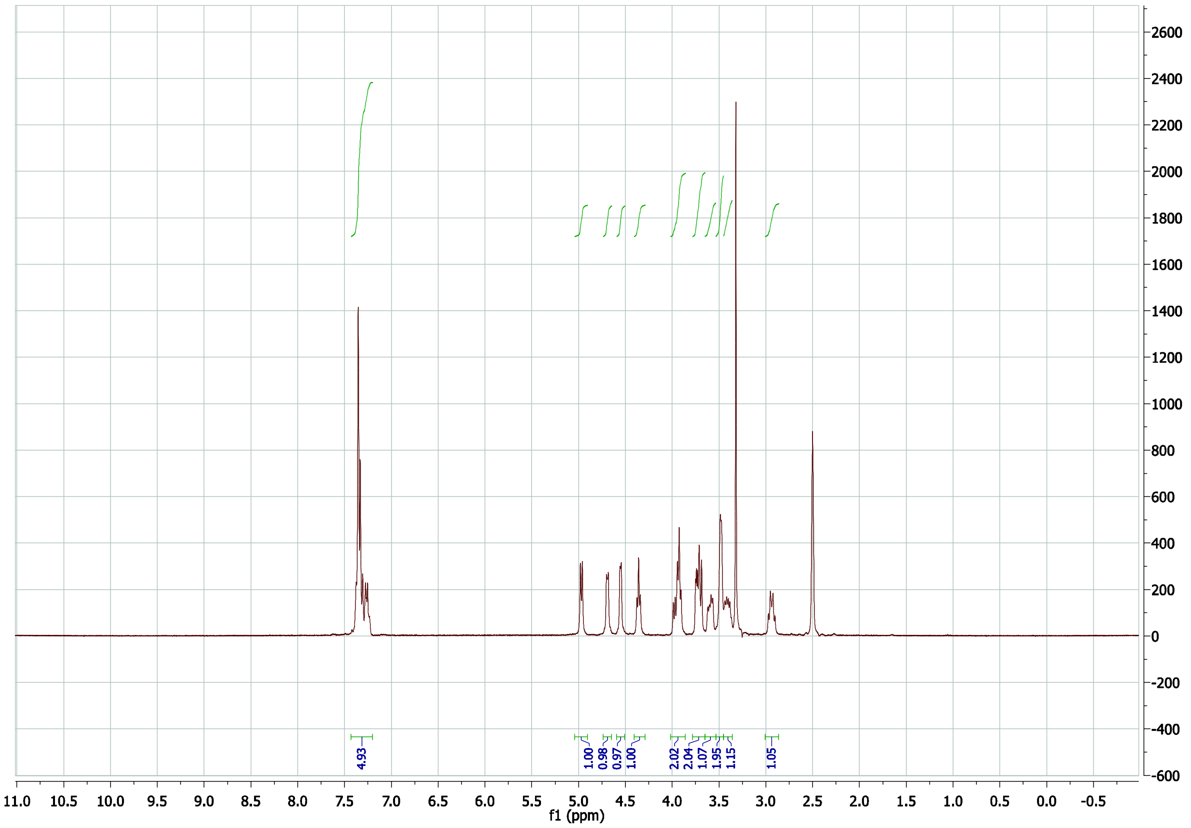

Compound **9**. ^{13}C APT (DMSO-d6)

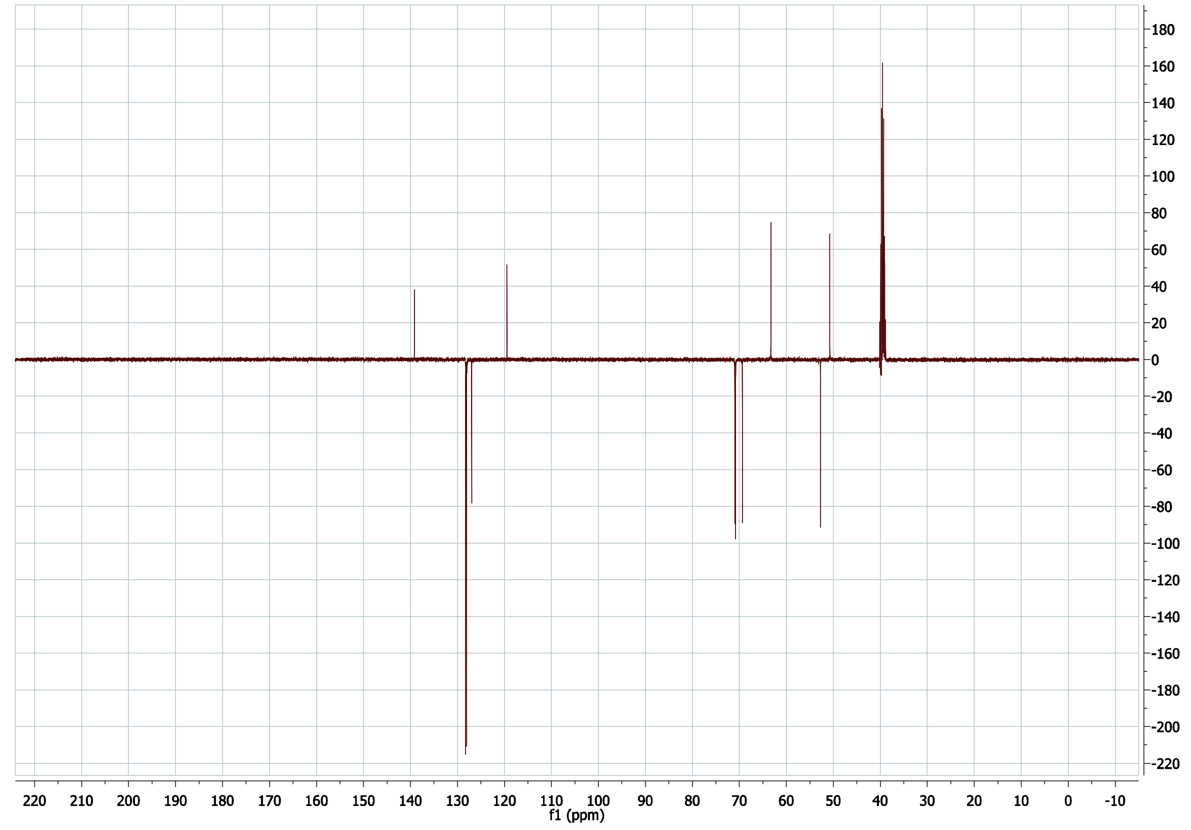

Compound **10**. ^{1}H NMR (DMSO-d6)

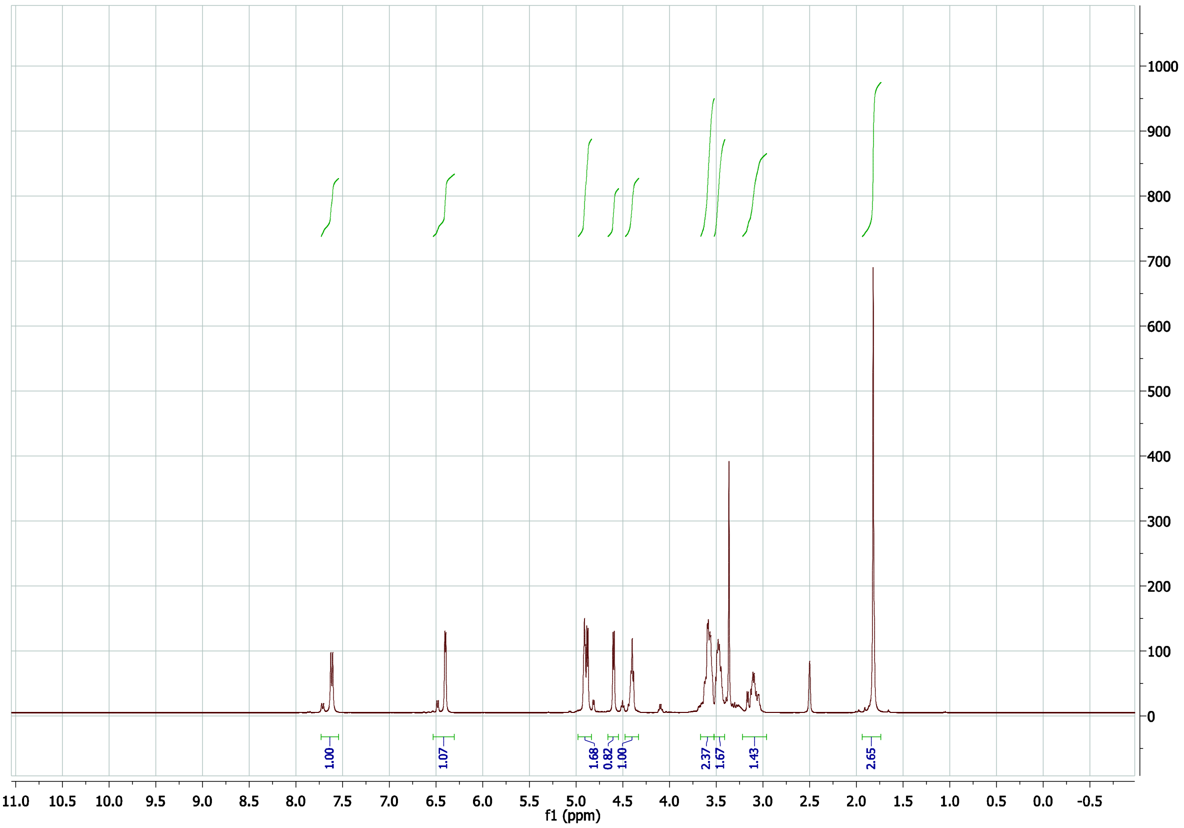

Compound **10**. ^{13}C APT (DMSO-d6)

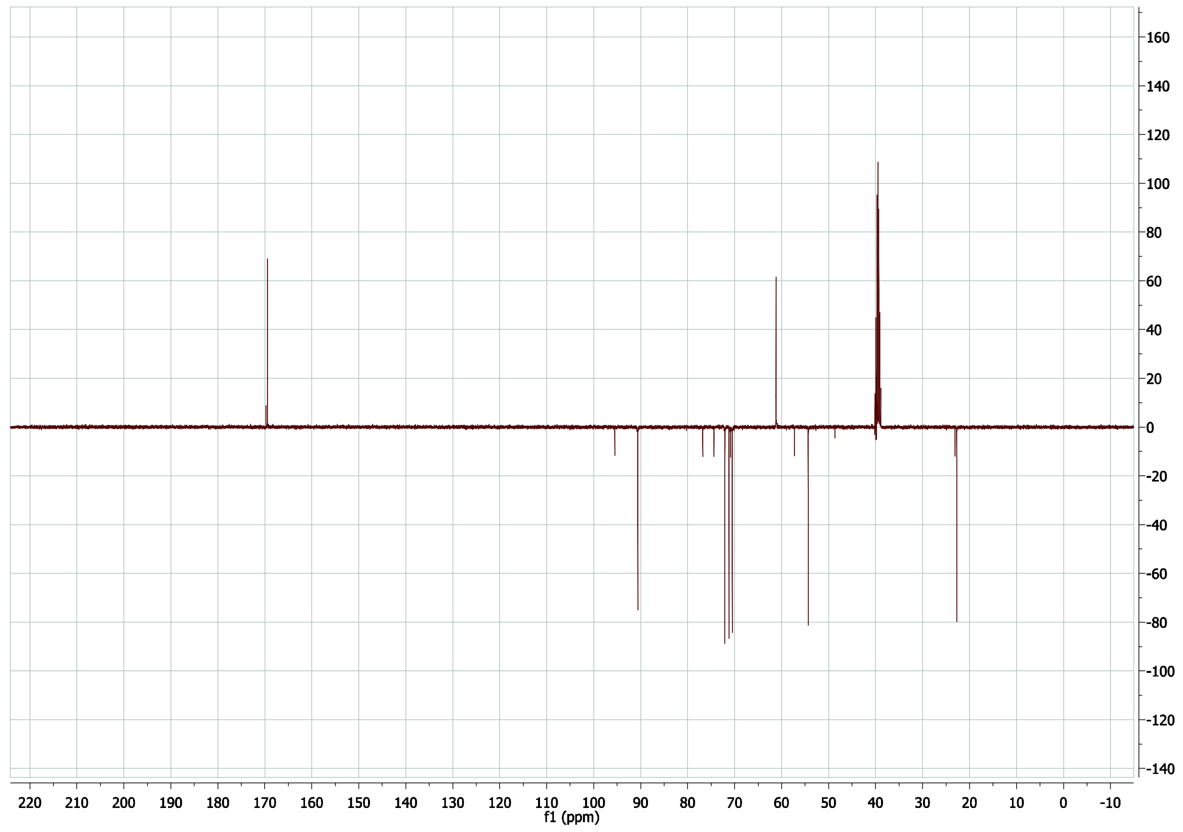

Compound **14** ^{13}C HSQC (D_2O/H_2O 9:1)

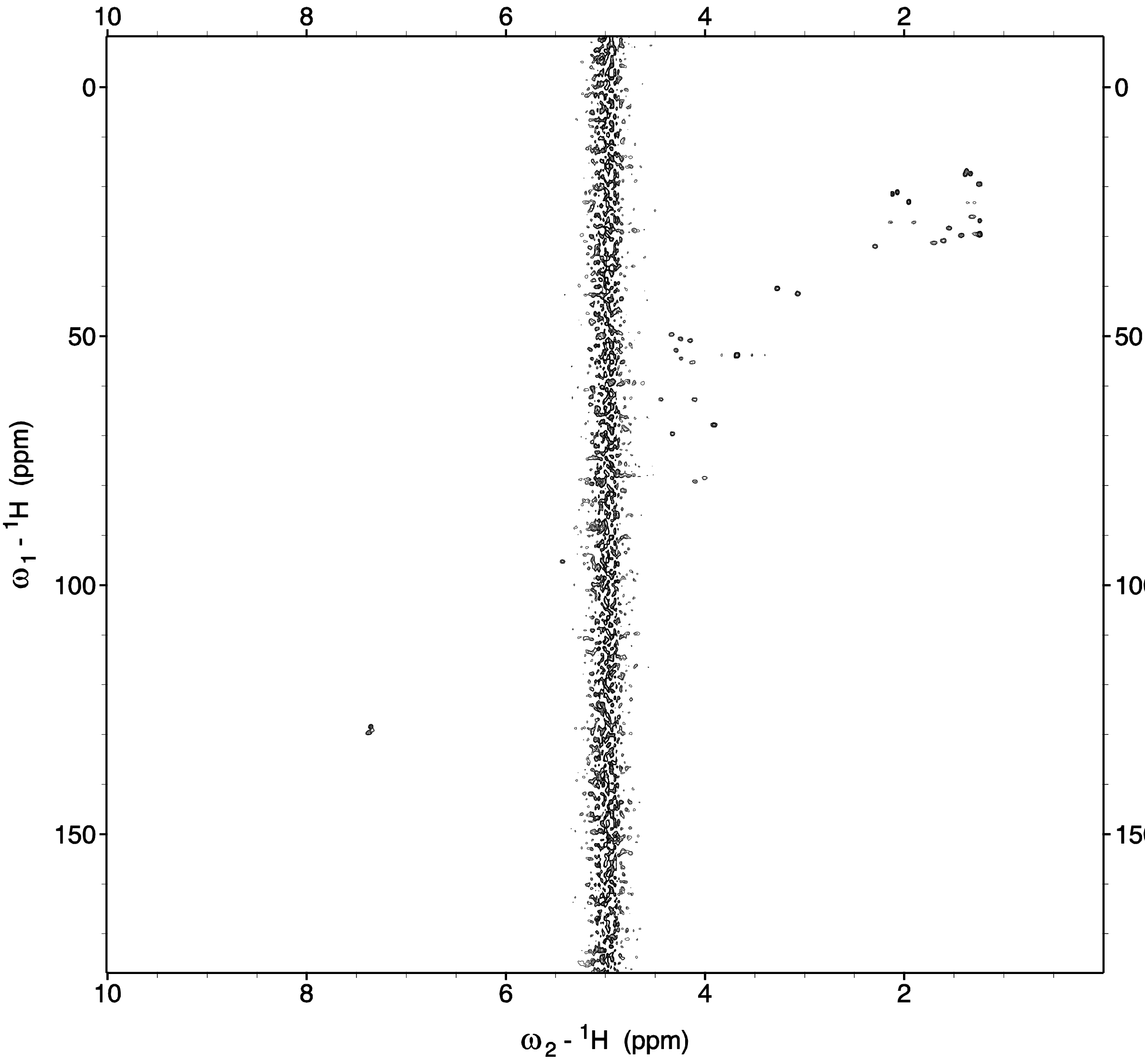

Compound **14** NOESY (D_2O/H_2O 9:1)

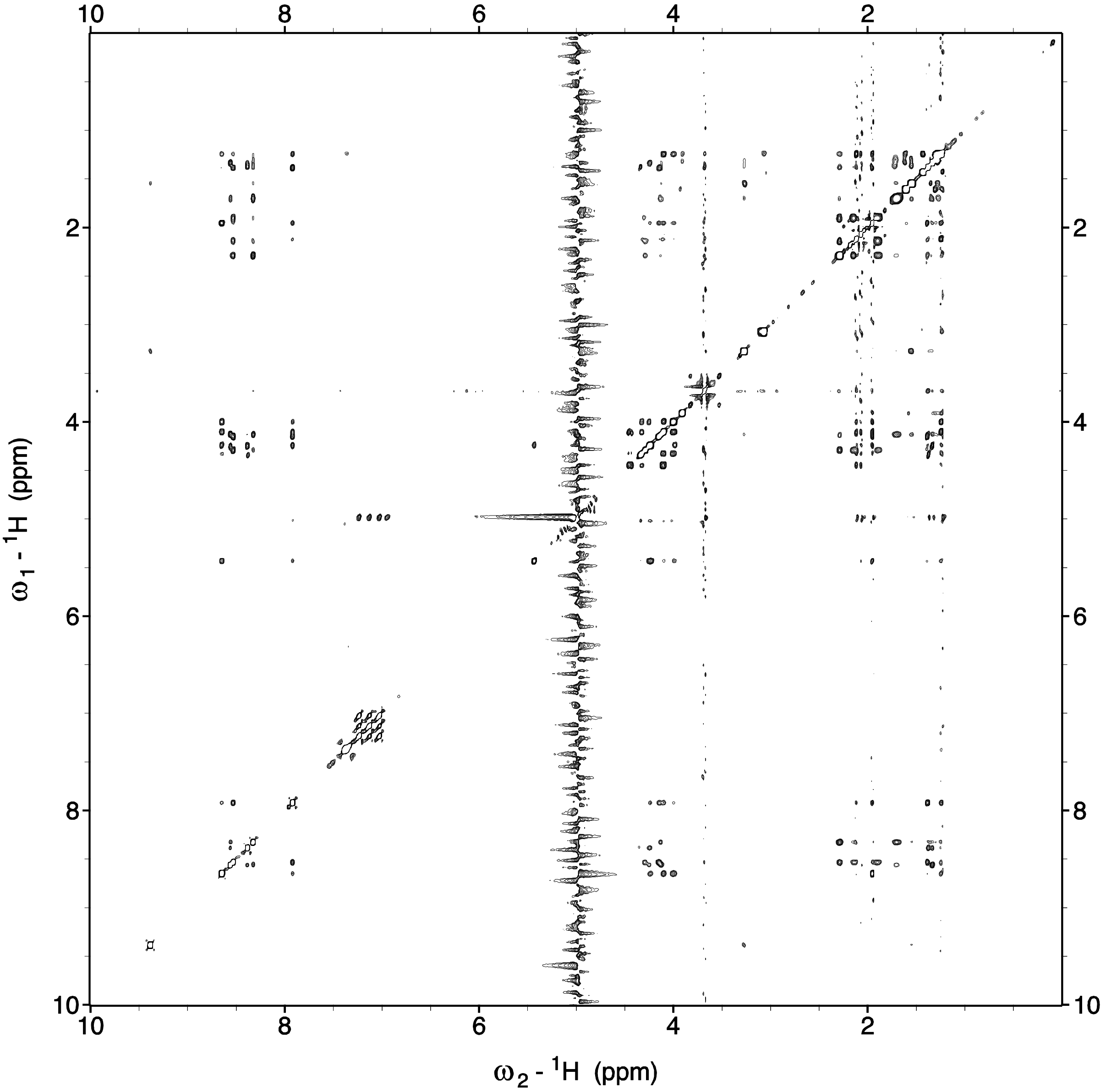

Compound **14** TOCSY (D_2O/H_2O 9:1)

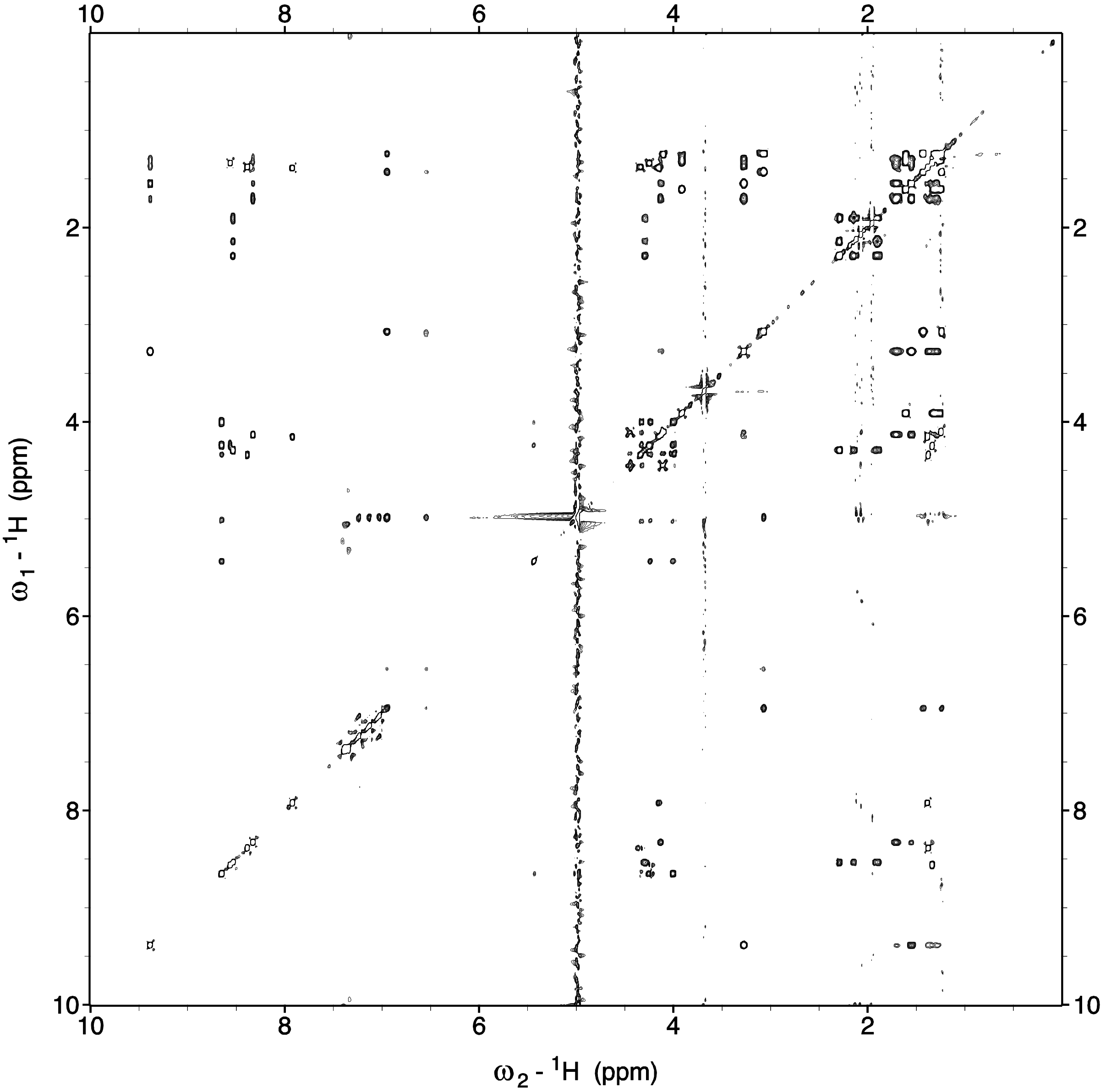

Compound **2** ^{13}C HSQC (D_2O/H_2O 9:1)

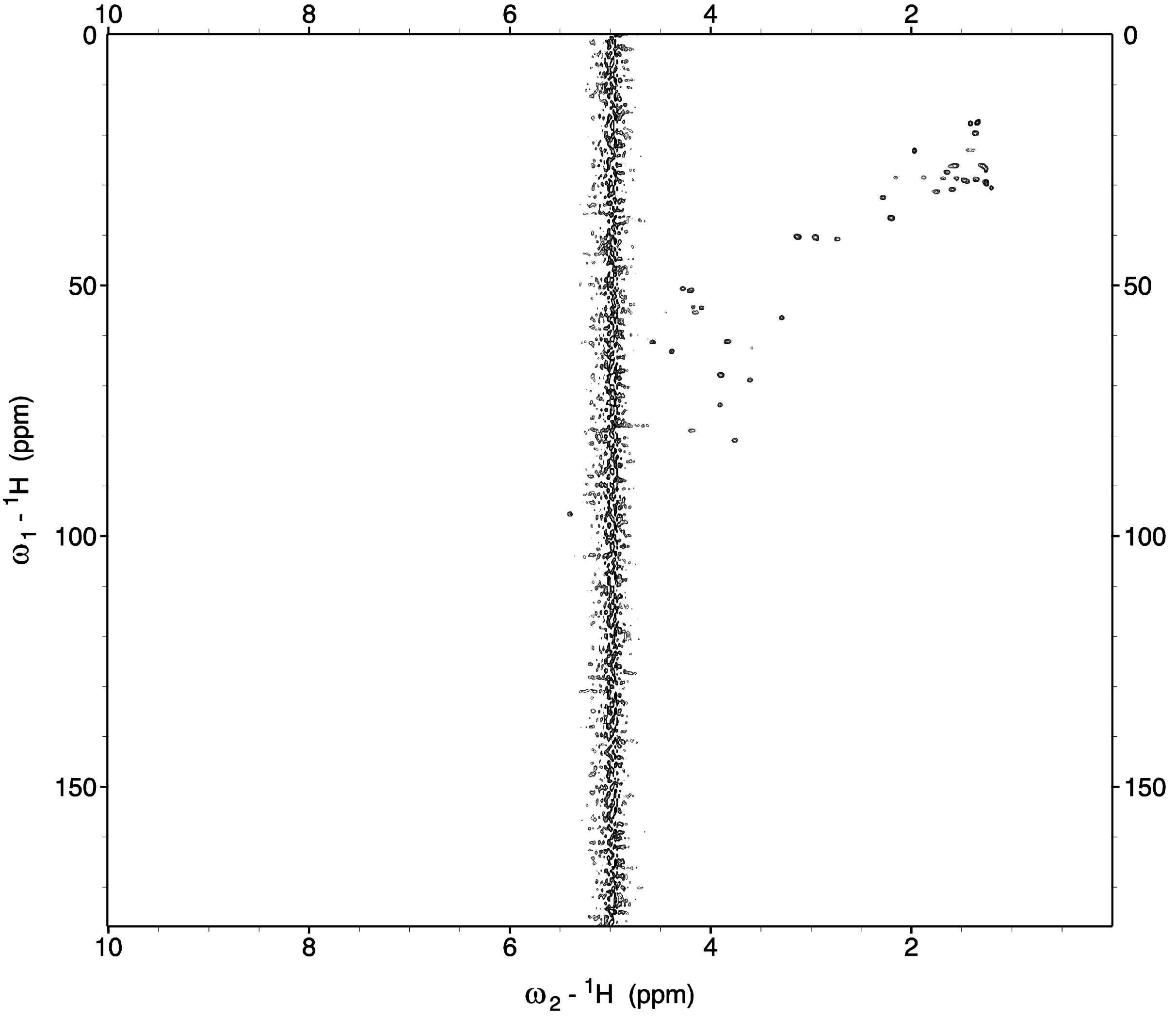

Compound **2** NOESY (D_2O/H_2O 9:1)

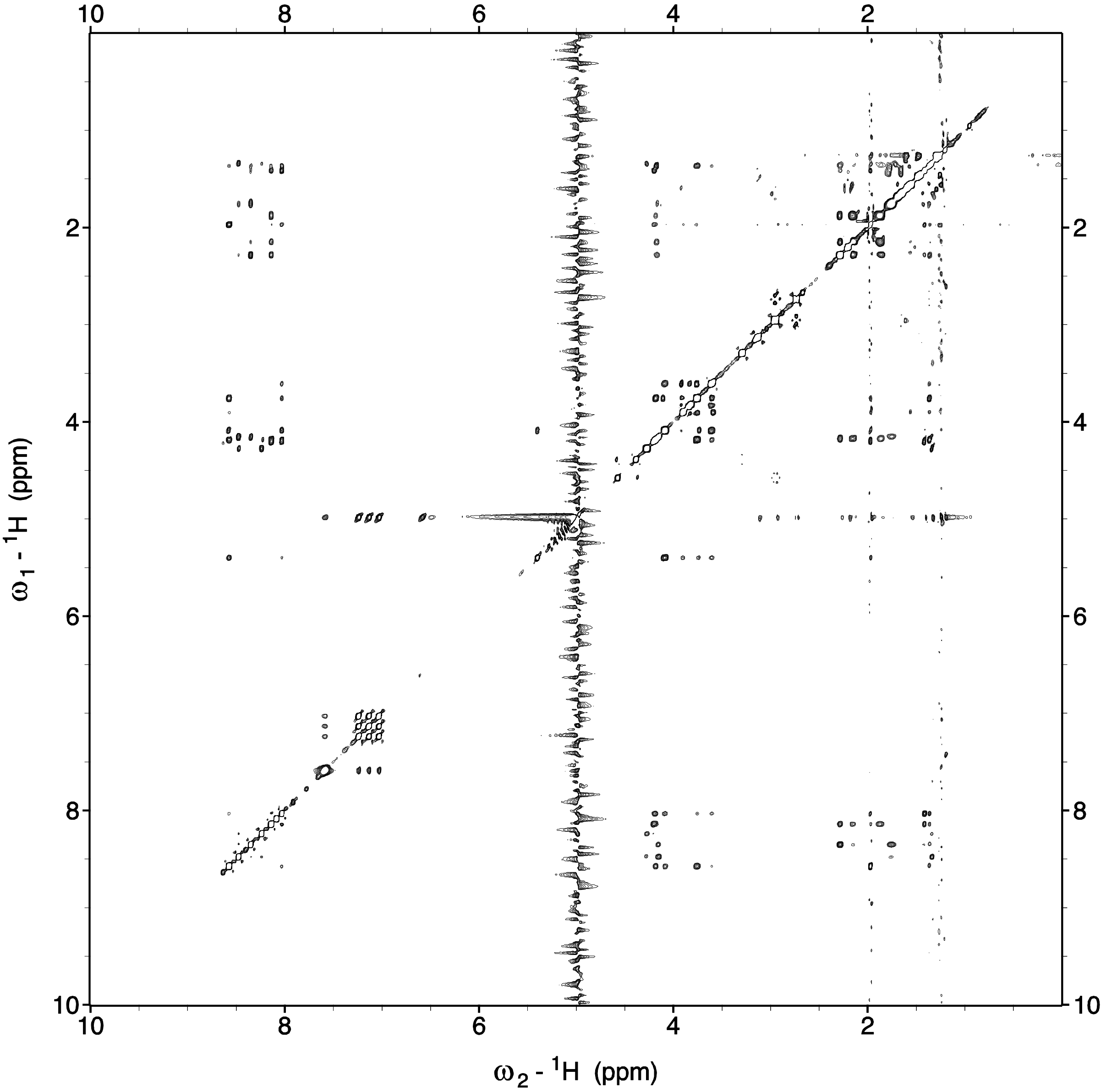

Compound **2** TOCSY (D_2O/H_2O 9:1)

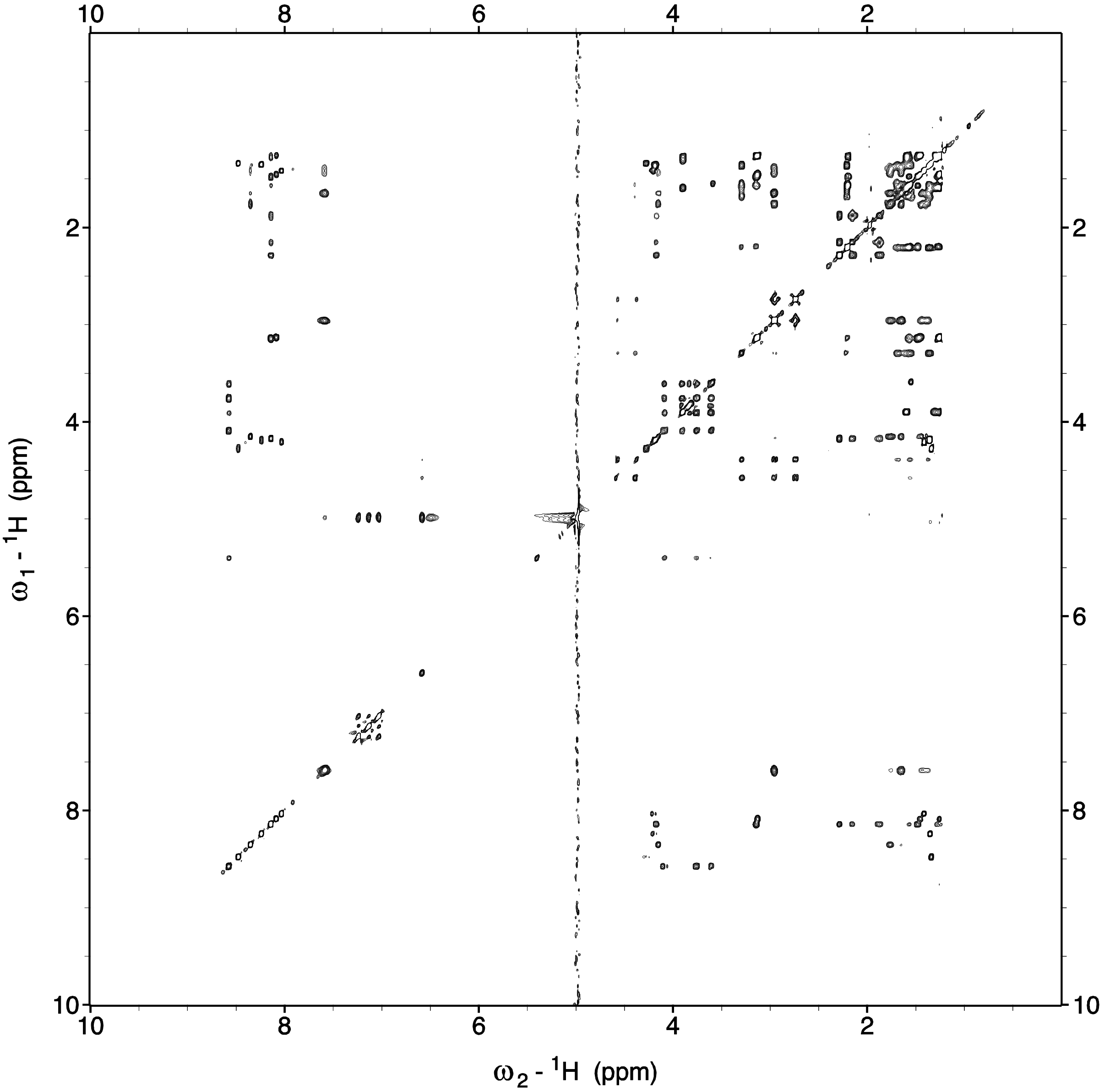

1.5 2D NMR analysis of intermediates **8** and **14** and final compounds **1** and **2**

1.5.1 NMR analysis of compound **14**

The results of the NMR analysis are shown in table 1. TOCSY, NOESY and HSQC (figure 9 and 10) were measured in H_2O/D_2O (9:1) and COSY in D_2O. Identical spectra were recorded for the enantiomeric compound **8**.

Supplemental figure 1: Structure of compound **14** and numbering used for the assignment of NMR peaks.

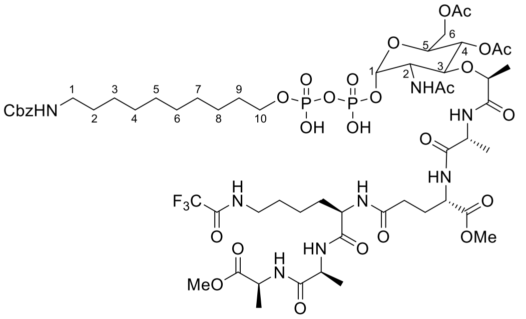

Supplemental figure 2: Cutout of HSQC spectrum of compound **14**. Outside of this view are the signal for the anomeric centre of MurNAc (C1) and the aromatic signals of the Cbz-group.

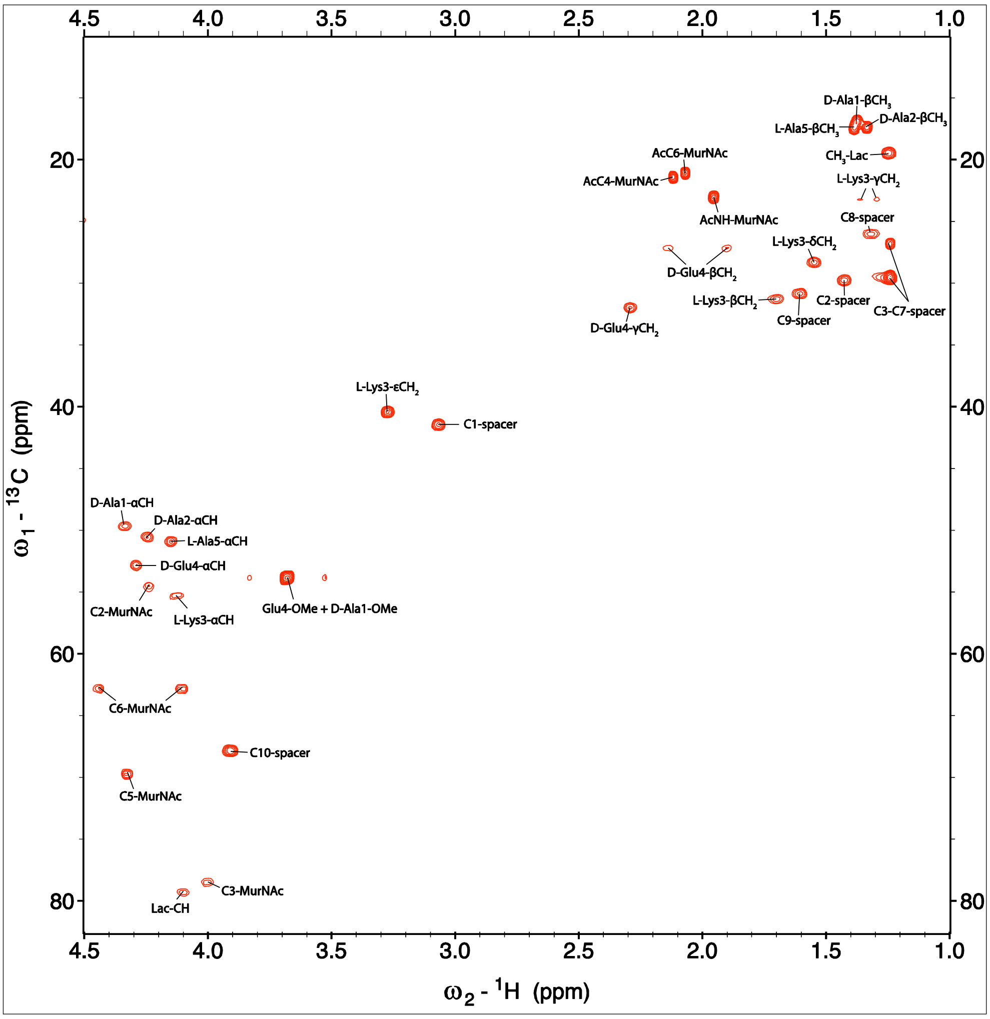

Supplemental figure 3: left: detail of the TOCSY spectrum of compound **14** showing the assigned spinsystems. Right: detail of the NOESY spectrum of compound **14** indicating the correct connectivity of all amide bonds.

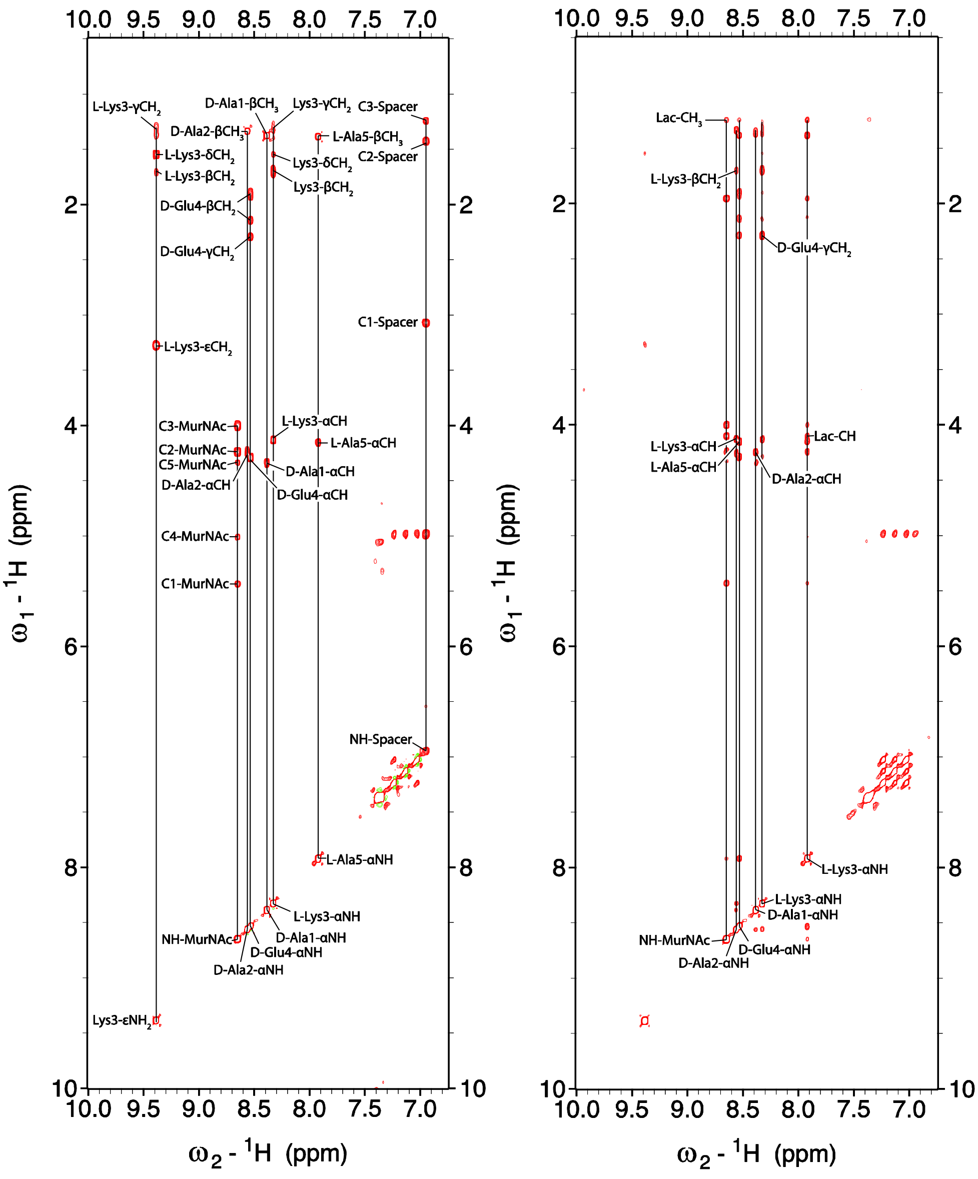

Supplemental table S1: Chemical shift assignments for compound **14**. See supplemental figure 1 for numbering scheme.

Carbohydrate	Peaks ^{1}H (δ in ppm)	Peaks ^{13}C (δ in ppm)
C1	5.44	95.3
C2	4.24	54.5
C3	4.01	78.5
C4	5.01	Overlaps with solvent
C5	4.33	69.8
C6	4.45, 4.13	62.8
Lactic acid	4.10 (CH), 1.25 (CH_3)	79.2 (CH), 19.5 (CH_3)
NH-C2	8.65	
Acetyl-NH	1.95	23.1
Acetyl-C4	2.12	21.4
Acetyl-C6	2.07	21.2

Pentapeptide		
D-Ala1	8.39 (NH), 4.34 (αCH), 3.68 (OCH_3), 1.38 (βCH_3)	49.7 (αCH), 53.9 (OCH_3), 16.9 (βCH_3)
D-Ala2	8.56 (NH), 4.24 (αCH), 1.34 (βCH_3)	50.6 (αCH), 17.4 (βCH_3)
L-Lys3	9.40 (εNH_2), 8.33 (αNH), 4.13 (αCH), 3.27 (εCH_2), 1.70 (βCH_2), 1.55 (δCH_2), 1.37 (γCH_2-1), 1.31 (γCH_2-2),	55.3 (αCH), 40.4 (εCH_2), 31.2 (βCH_2), 28.3 (δCH_2), 23.2 (γCH_2-1 + γCH_2-2)
D-Glu4	8.54 (NH), 4.29 (αCH), 3.68 (OCH_3), 2.29 (γCH_2), 2.15 (βCH_2-1), 1.91 (βCH_2-2)	52.9 (αCH), 53.9 (OCH_3), 32.0 (γCH_2), 27.1 (βCH_2-1 + βCH_2-2)
L-Ala5	7.92 (NH), 4.16 (αCH), 1.39 (βCH_3)	50.9 (αCH), 17.5 (βCH_3)
**Spacer**		
C10-chain	6.94 (NH), 3.91 (C10), 3.07 (C1), 1.61 (C9), 1.43 (C2), 1.33 (C8), 1.28 – 1.24 (C3-7),	67.8 (C10), 41.5 (C1), 30.8 (C9), 29.7 (C2), 26.0 (C8), 29.5 + 26.8 (C3-7)
Cbz-group	7.38, 7.36, 7.34 (aromatic), 5.05 (CH_2)	129.6, 128.5, 129.1 (aromatic), CH_2 overlaps with solvent

1.5.2 NMR assignments of compound **2**

The results of the NMR analysis are shown in supplemental table 2. TOCSY, NOESY and HSQC were measured in H_2O/D_2O (9:1) and COSY in D_2O. The same set of spectra were recorded for the enantiomeric compound **1**.

Supplemental figure 4: Structure of compound **2** and numbering used for the assignment of NMR peaks.

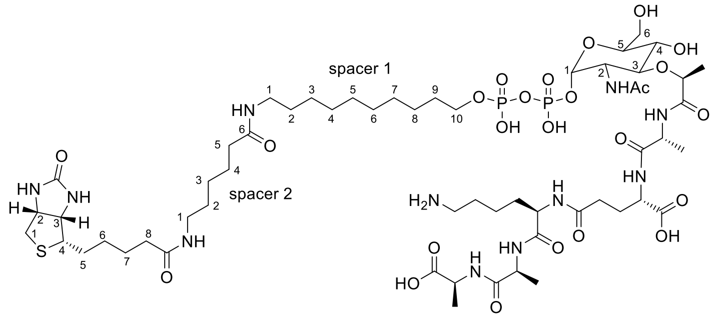

Supplemental figure 5: Cutout of HSQC spectrum of compound **2**. Outside of this view is the signal for the anomeric centre of MurNAc (C1).

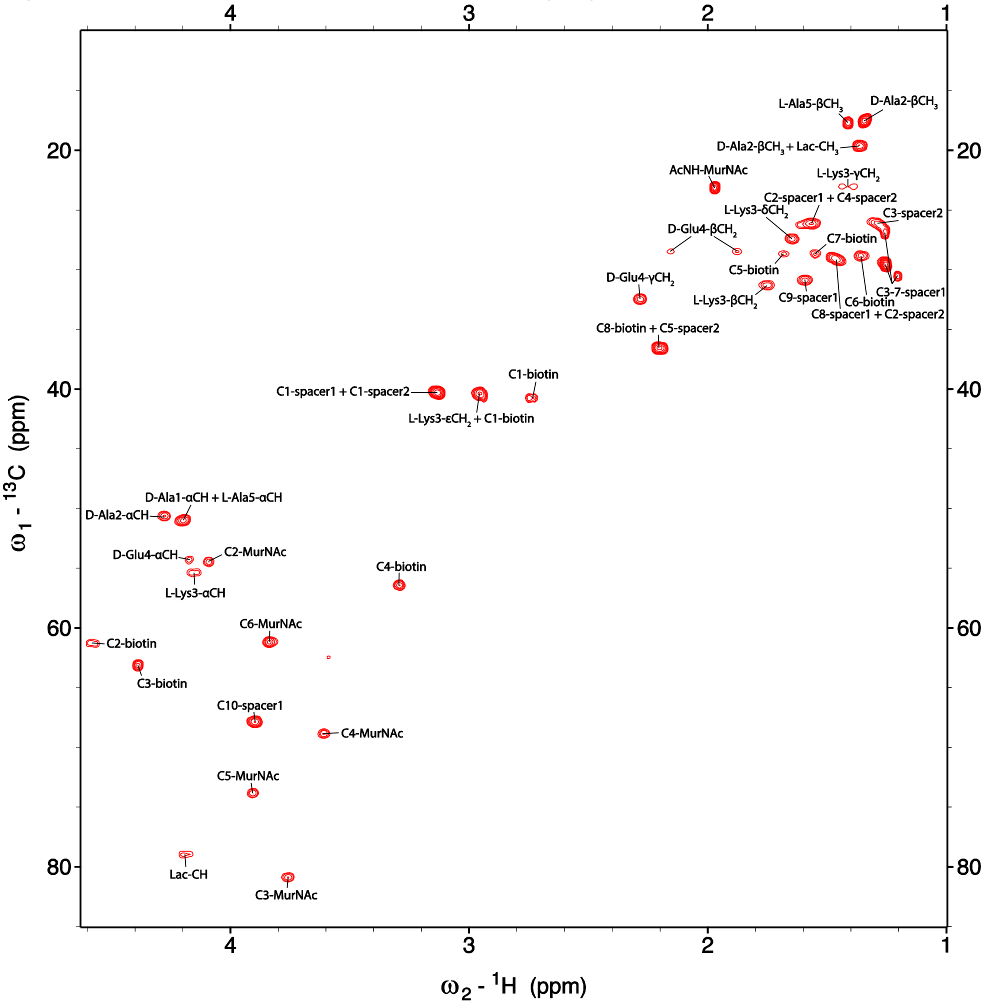

Supplemental figure 6: A) Detail of the TOCSY spectrum of compound **2** showing the assigned spinsystems. B) Detail of the NOESY spectrum of compound **2** indicating the correct connectivity of all amide bonds

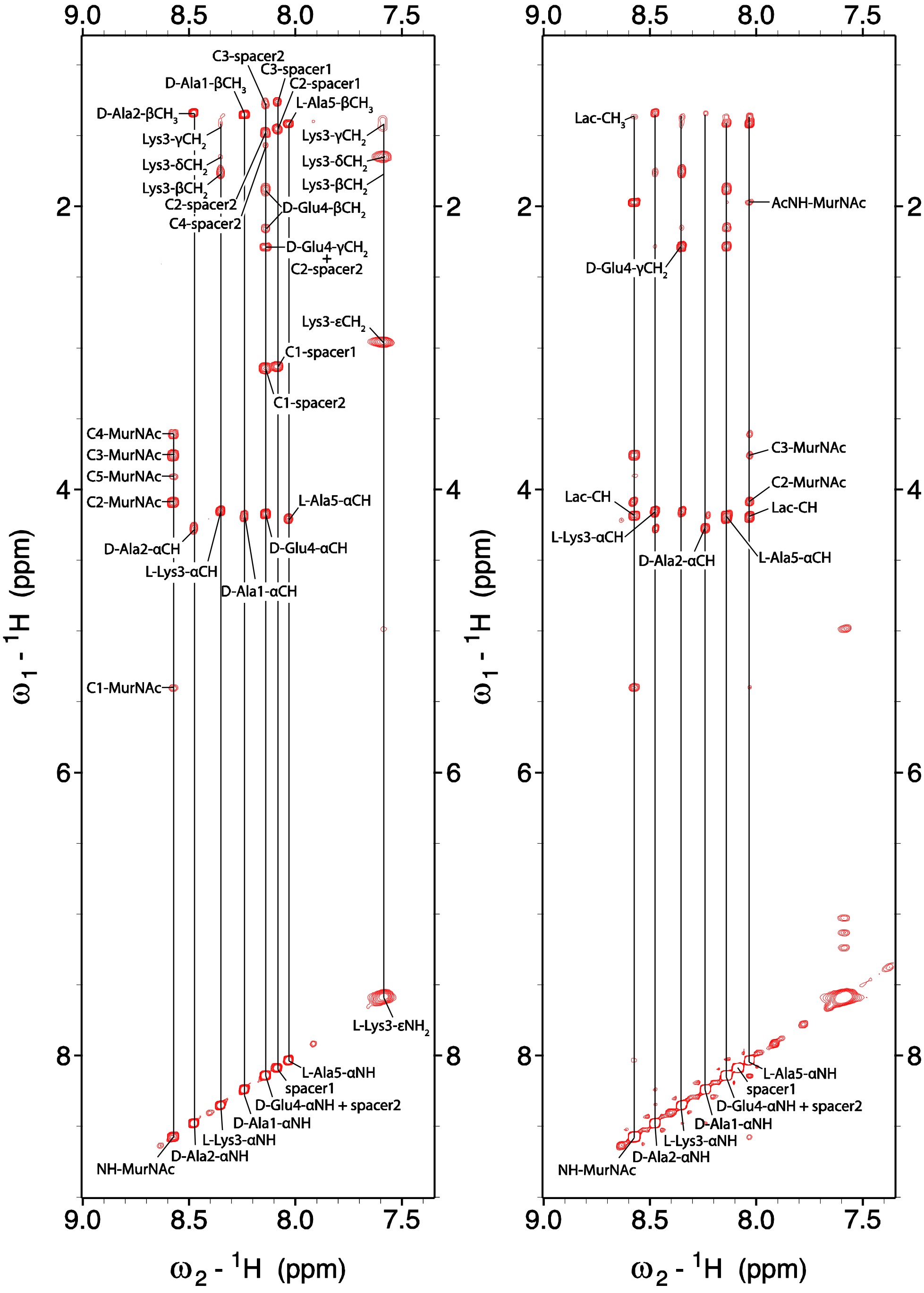

Supplemental table S2: Chemical shift assignments for compound **2**. See figure 4 for numbering scheme.

Carbohydrate	Peaks ^{1}H (δ in ppm)	Peaks ^{13}C (δ in ppm)
C1	5.40	95.6
C2	4.09	54.5
C3	3.76	80.8
C4	3.61	68.9
C5	3.91	73.9
C6	3.83	61.2

Lactic acid	4.18 (CH), 1.37 (CH_3)	80.0 (CH), 19.6 (CH_3)
Acetyl-NH	1.97	23.1
NH-C2	8.58	
Pentapeptide		
D-Ala1	8.24 (NH), 4.19 (αCH), 1.35 (βCH_3)	51.1 (αCH), 19.6 (βCH_3)
D-Ala2	8.48 (NH), 4.28 (αCH), 1.34 (βCH_3)	50.7 (αCH), 17.5 (βCH_3)
L-Lys3	7.59 (εNH_2), 8.36 (αNH), 4.15 (αCH), 2.96 (εCH_2), 1.76 (βCH_2), 1.65 (δCH_2), 1.41 (γCH_2)	55.4 (αCH), 40.3 (εCH_2), 31.3 (βCH_2), 27.4 (δCH_2), 23.1 (γCH_2)
D-Glu4	8.14 (NH), 4.17 (αCH), 2.29 (γCH_2), 2.16 (βCH_2-1), 1.88 (βCH_2-2)	54.4 (αCH), 32.5 (γCH_2), 28.4 (βCH_2-1 + βCH_2-2)
L-Ala5	8.03 (NH), 4.21 (αCH), 1.42 (βCH_3)	51.1 (αCH), 17.8 (βCH_3)
Spacers + biotin		
C10-spacer	8.08 (NH), 3.90 (C10), 3.13 (C1), 1.59 (C9), 1.57 (C2), 1.47 (C8), 1.28 - 1.20(C3-7),	67.9 (C10), 40.3 (C1), 30.9 (C9), 26.2 (C2), 29.1 (C8), 30.6 – 26.2 (C3-7)
C6-spacer	8.14 (NH), 3.13 (C1), 2.21 (C5), 1.57 (C4), 1.46 (C2), 1.28 (C3)	40.3 (C1), 36.6 (C5), 26.2 (C4), 29.1 (C2), 26.2 (C3)
Biotin	6.59 (NHx2) 4.57 (C2), 4.39 (C3), 3.29 (C4), 2.96 + 2.75 (C1), 2.21 (C8), 1.68 (C5), 1.55 (C7), 1.36 (C6)	61.3 (C2), 63.2 (C3), 56.5 (C4), 40.7 (C1), 36.6 (C8), 28.7 (C5), 28.7 (C7), 28.9 (C6)

1.6 HPLC analysis of intermediates **8** and **14** and final compounds **1** and **2**

Compound **8**

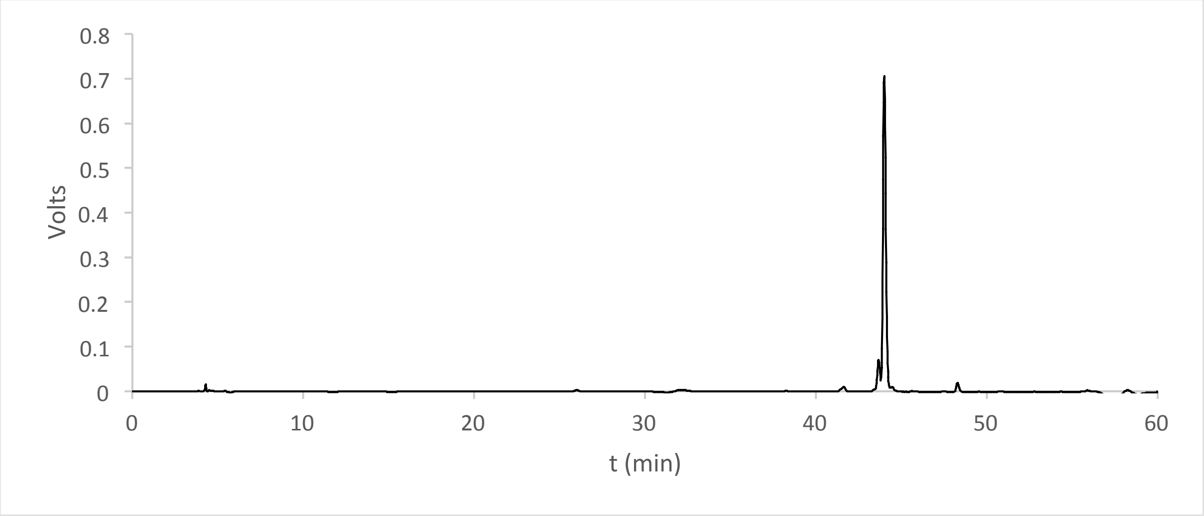

Compound **14**

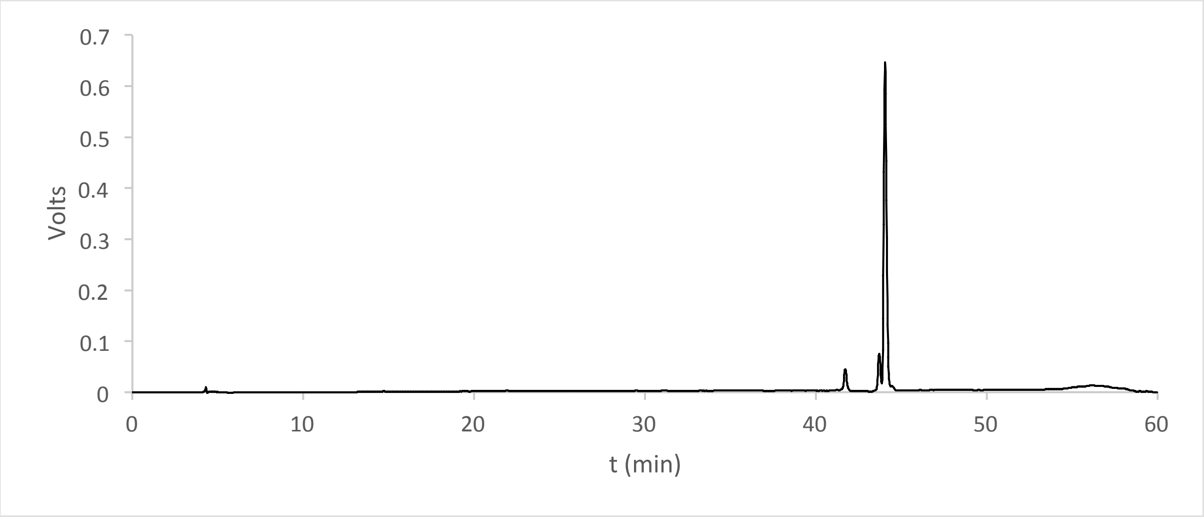

Overlay of compounds **8** and **14**

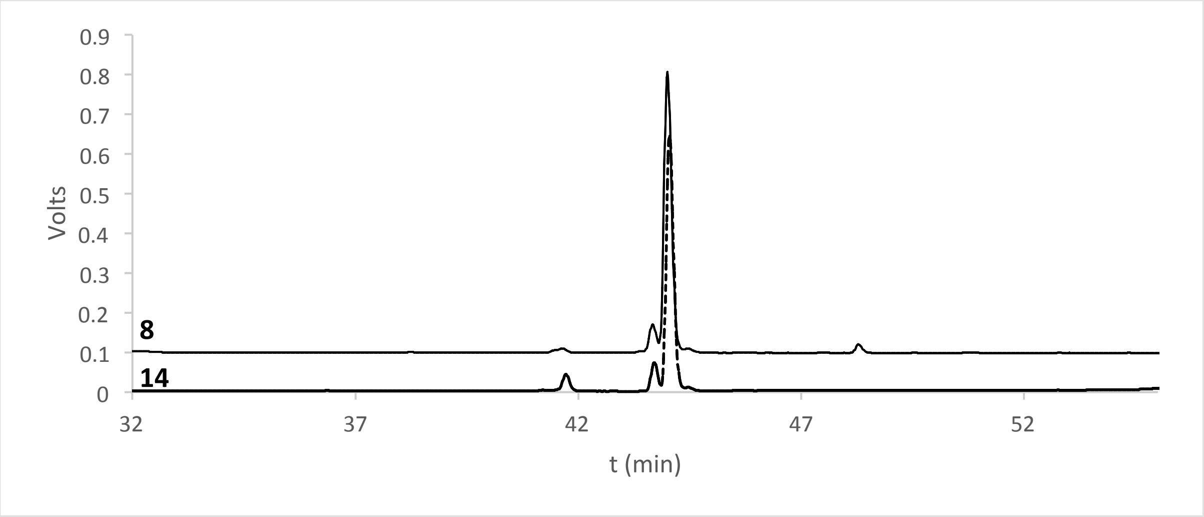

Compound **1**

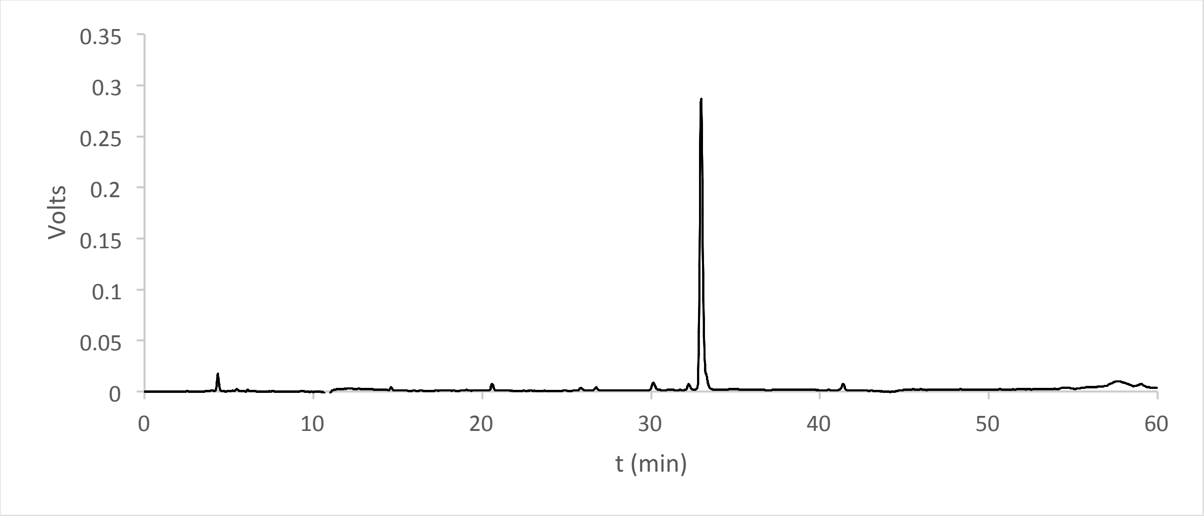

Compound **2**

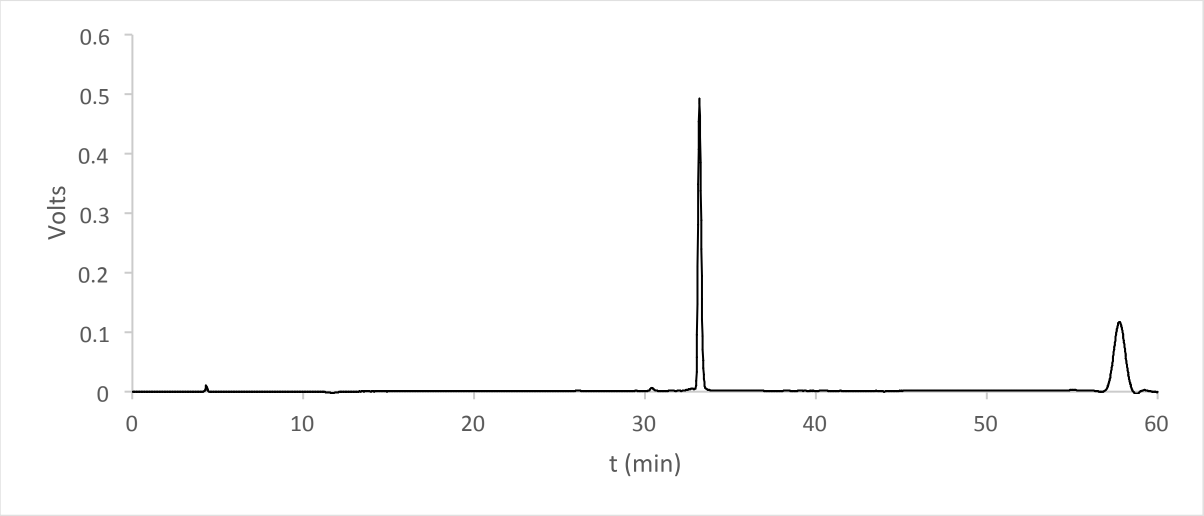

Overlay of compound **1** and **2**

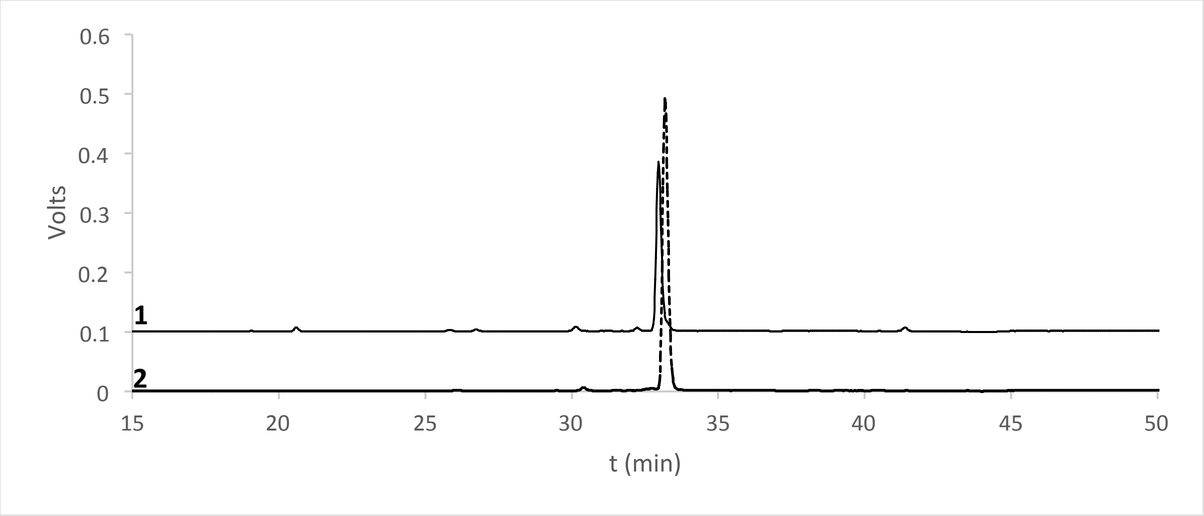

Section 2 Phage display

2.1 Phage selection

Bicyclic phage production was performed in a 400 mL culture following the previously described protocol.[7] After the phage production they were stored in 2.4 ml buffer W (10 mM Tris-Cl, 150 mM NaCl, 10 mM $MgCl_2$, 1 mM $CaCl_2$, pH 7.4). Phage were blocked by addition of 1.2 ml buffer W3 (buffer W + 3% BSA and 0.3% Tween-20) and rotated at 10 rpm for 30 min. Next they were split into 2 equal portions and cooled on ice. On portion received target 1 and the other target 2 both at a final concentration of 1 µM. Both tubes were incubated overnight by rotation at 10 rpm at 4°C. Blocked streptavidin coated magnetic beads (50 µl, precooled on ice) were added and the mixture was rotated at 10 rpm for 15 min at 4°C. The beads were washed with 4x with buffer W4 (buffer W + 0.1% Tween-20) and 2x with buffer W (all washing performed at 4°C). Captured phage were then eluted by treatment with 100 µl buffer G (50 mM glycine, pH 2.2) for 5 min at 4°C and then neutralized by addition of 50 µl buffer F (1M Tris, pH 8.0). From here the protocol was followed as previously described.[7]

2.2 Phage analysis

High-throughput DNA analysis of phage pools was performed as previously described.[8]

Supplemental table S3: top hits in HTS analysis of eluted phage for target 1 (total reads: 135272).

Peptide nr (or reason for omission[a])	Sequence	Abundance nr in HTS.	Abundance %[b]
[a]*Cys to Ser mutant*	A**C**GAADWG**C**TLLLTR**S**G	16225	12.0
P1	A**C**DWPDWQ**C**YGWSSH**C**G	5703	4.2
P2	A**C**KHQAQE**C**VAIREG**C**G	4252	3.1
P3	A**C**WAMPMW**C**DSWSQN**C**G	2446	1.8
P4	A**C**RPQKFN**C**ISANIR**C**G	2006	1.5
[a]*Cys deletion*	A**C**QLEWLG**-**ARYDLR**C**G	1757	1.3
[a]*Extra cysteine mutant*	A**C**WGWGVE**C**QSE**C**WF**C**G	1608	1.2
P5	A**C**KAMIGA**C**VAMQFA**C**G	1582	1.2
P6	A**C**YPVDWY**C**LFQTVD**C**G	1493	1.1
P7	A**C**RYVSGD**C**YYAQAH**C**G	1313	1.0
<1 %	A**C**QVAATG**C**GVLLQI**C**G	1271	0.9

[a]Peptides that did not contain the exact number of three expected cysteines were omitted as their exact structure upon cyclization prior to phage selection is unknown. [b]All phage clones that had an abundance > 1% were considered for synthesis and evaluation.

Supplemental table S4: top hits in HTS analysis of eluted phage for target 2 (total reads: 192947)

Peptide nr	Sequence	Abundance nr in HTS	Abundance %
P8	A**C**LLQSLL**C**PYSTHR**C**G	188413	97.7
<1 %	A**C**PQKVQA**C**ANDVLE**C**G	209	0.1
<1 %	A**C**REASVV**C**GSTQLS**C**G	147	<0.1
<1 %	A**C**SLSAKF**C**WHGENQ**C**G	147	<0.1
<1 %	A**C**KHGETY**C**SQVPSS**C**G	111	<0.1

[7] I. Rentero Rebollo, C. Heinis, *Methods* **2013**, *60*, 46–54.

[8] I. Rentero Rebollo, M. Sabisz, V. Baeriswyl, C. Heinis, *Nucleic Acids Res.* **2014**, *42*, e169–e169.

Section 3 Peptide synthesis

3.1 Non-lipidated peptides

Peptides were synthesized on either 0.05 mmol scale using a Protein Technologies Symphony peptide synthesizer or on 0.1 and 0.25 mmol scale using a CS Bio 336X peptide synthesizer. Non-lipidated peptides were prepared on Rink-amide resin (Rapp polymere GmbH) using standard Fmoc/tBu solid phase peptide synthesis conditions. Each peptide was cleaved from the resin and deprotected by treating it with TFA/H_2O/EDT/TIPS (90:5:2.5:2.5, 5 ml per 0.05 mmol) for 1 h, followed by filtration and precipitation in MTBE/Hex (1:1, 50ml per 0.05 mmol). The peptide suspension was centrifuged at 3500 rpm for 5 min and the obtained pellets were suspended in MTBE/Hex again followed by centrifugation and this was repeated once more. The obtained pellets were then dissolved in a mixture of 20 mM NH_4HCO_3 and MeCN (typically 3:1 but ratio can be varied depending on peptide solubility, total volume should be 90 ml per 0.05 mmol). TBMB (0.075 mmol; 26.8 mg) was added as a solution in MeCN (5 ml). The reaction mixture was stirred for 1 h at room temperature followed by evaporation of MeCN and subsequent lyophilisation. Peptides were purified by preparative HPLC using a Dr. Maisch Reprosil-Pur 120 C18-AQ column (250 x 25 mm, 10 µm) using a gradient of 25% buffer B to 75% buffer B over 45 min (buffer A: 95% H_2O, 5% MeCN, 0.1% TFA; buffer B: 5% H_2O, 95% MeCN, 0.1% TFA).

3.2 Lipidated peptides

Peptides were synthesized on either 0.05 mmol scale using a Protein Technologies Symphony peptide synthesizer or on 0.1 and 0.25 mmol scale using a CS Bio 336X peptide synthesizer. Lipidated peptides were prepared on Chlorotrityl chloride resin (Iris Biotech GmbH) using standard Fmoc/tBu solid phase peptide synthesis conditions. Peptides were cleaved from the resin by treating it with HFIP/DCM (1:3, 4ml per 0.05 mmol) for 1 h at room temperature. The resin was removed by filtration and the filtrate was evaporated to dryness. The protected peptide was dissolved in DCM (3 ml) and the lipid-amine of interest (0.5 mmol per 0.05 mmol peptide) was added, followed by BOP (44.2 mg, 0.1 mmol) and DIPEA (35 µl, 0.2 mmol). The mixture was stirred for 1 h before it is evaporated to dryness. The peptide was deprotected by treating it with TFA/H_2O/EDT/TIPS (90:5:2.5:2.5, 5 ml per 0.05 mmol) for 1 h and precipitated in MTBE/Hex (1:1, 50ml per 0.05 mmol). The peptide was centrifuged at 3500 rpm for 5 min and the obtained pellets were suspended in MTBE/Hex again followed by centrifugation and this was repeated once more. The obtained pellets were dissolved in 1.5 ml DMF and then sonicated to ensure good dissolution followed by further addition of a mixture of 20 mM NH_4HCO_3 and MeCN (typically 2:1 but ratio can be varied depending on peptide solubility, total volume should be 90 ml per 0.05 mmol). TBMB (0.075 mmol; 26.8 mg) was then added as a solution in MeCN (5 ml). The reaction mixture was stirred for 1 h at room temperature followed by evaporation of MeCN and subsequent lyophilisation. Peptides were purified by preparative HPLC using a Dr. Maisch Reprosil-Pur 120 C18-AQ column (250 x 25 mm, 10 µm) using a gradient of 40-100% buffer B over 72 minutes (buffer A: 95% H_2O, 5% MeCN, 0.1% TFA; buffer B: 5% H_2O, 95% MeCN, 0.1% TFA).

Supplemental scheme 8: Synthesis of C-terminally lipidated bicyclic peptides.

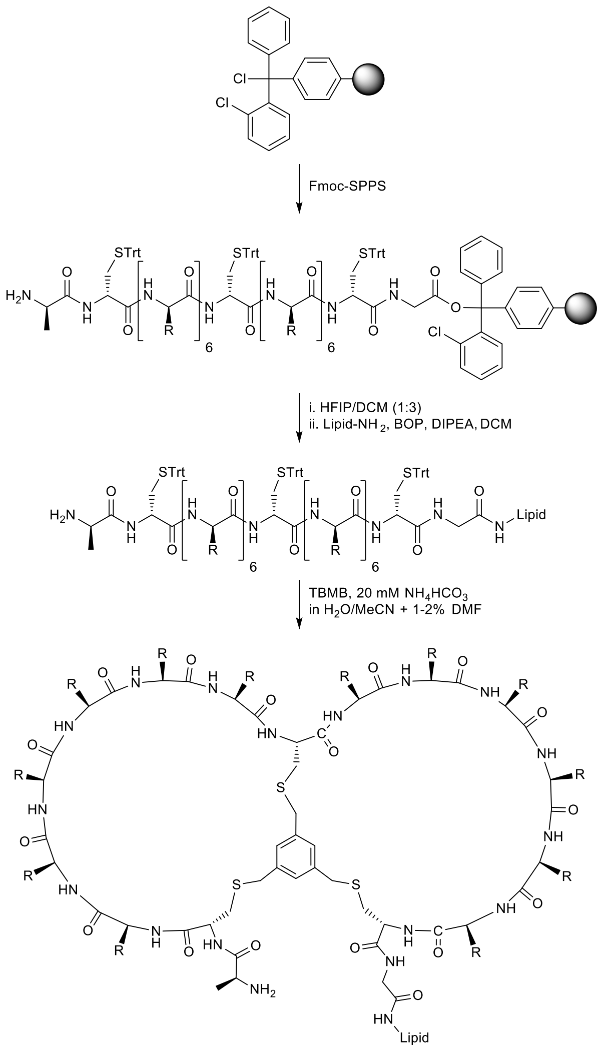

3.3 HPLC data for all peptides

P1

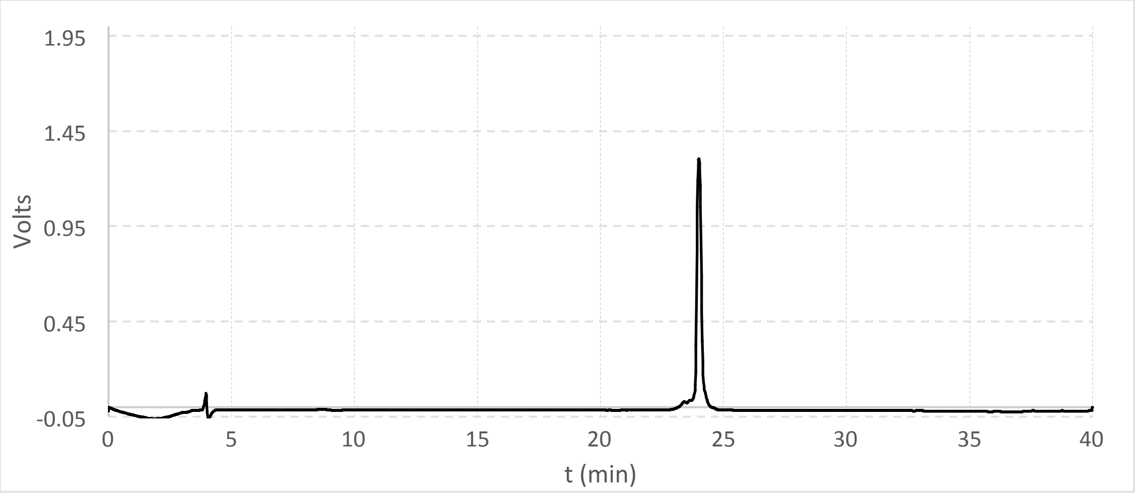

P2

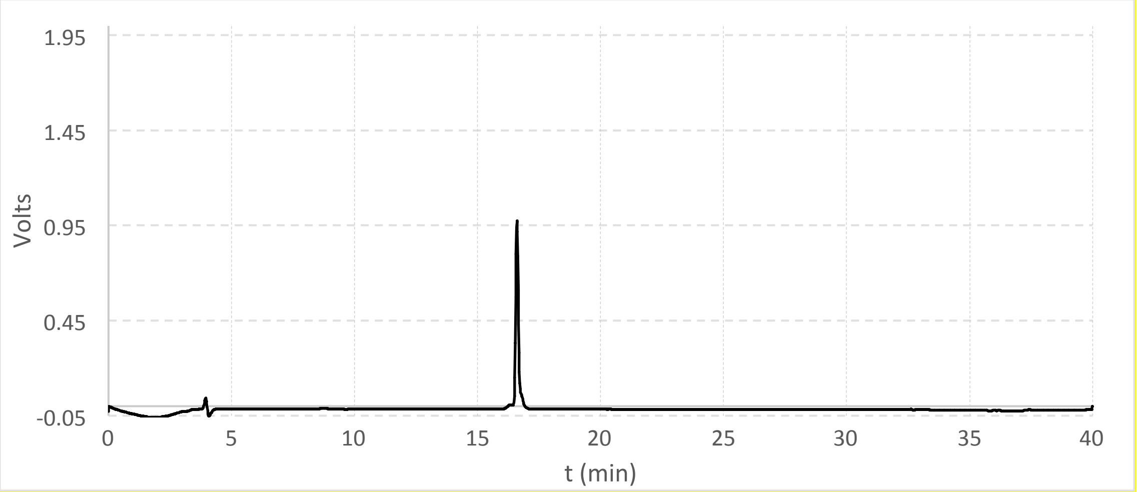

P3

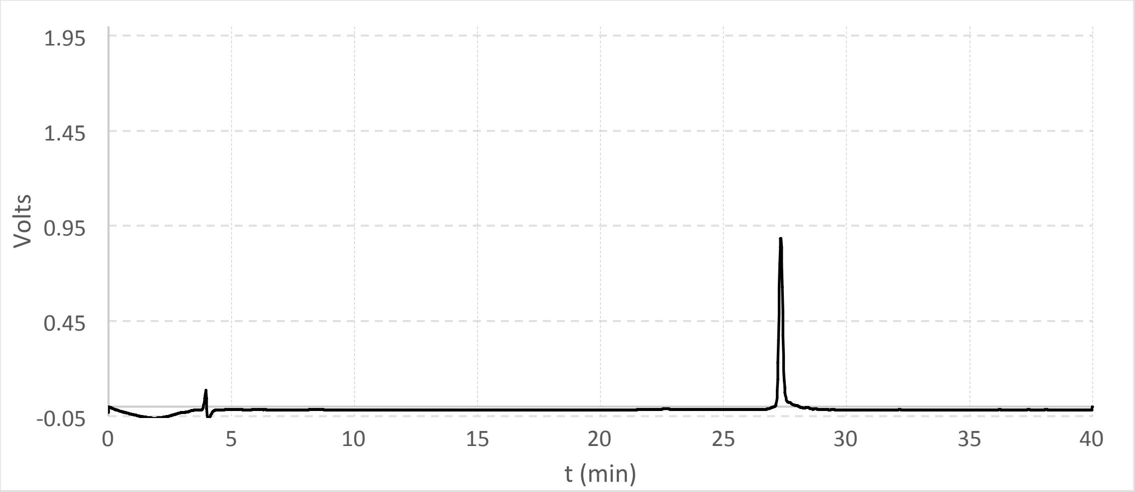

P4

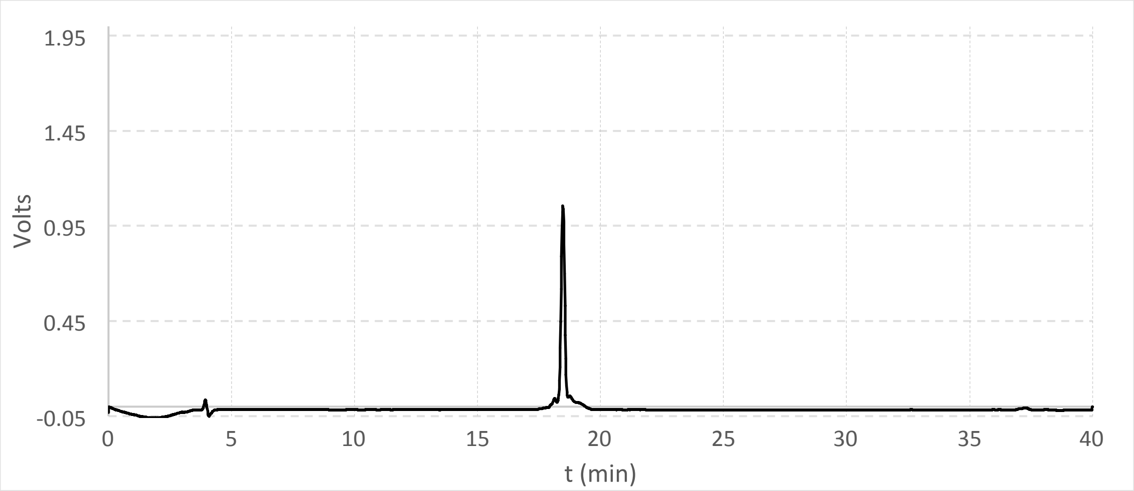
Volts
1.95
1.45
0.95
0.45
-0.05
0
5
10
15
20
25
30
35
40
t (min)

P6

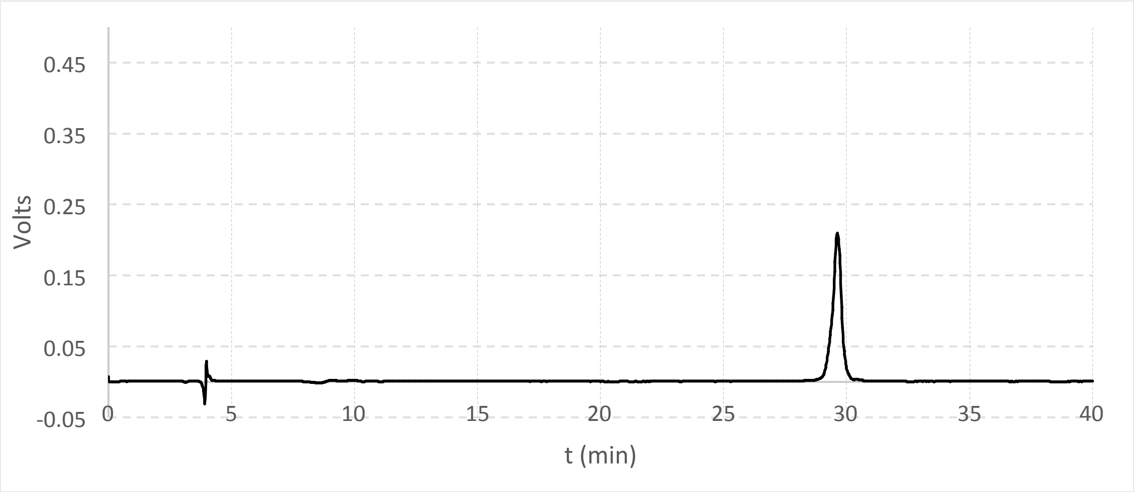
Volts
0.45
0.35
0.25
0.15
0.05
-0.05
0
5
10
15
20
25
30
35
40
t (min)

P7

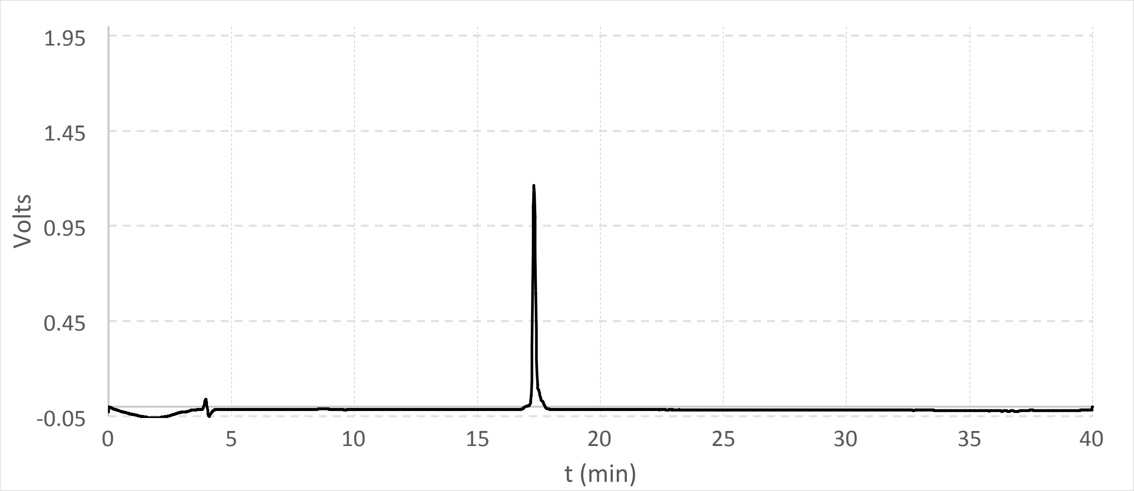
Volts
1.95
1.45
0.95
0.45
-0.05
0
5
10
15
20
25
30
35
40
t (min)

C_{10}-P4

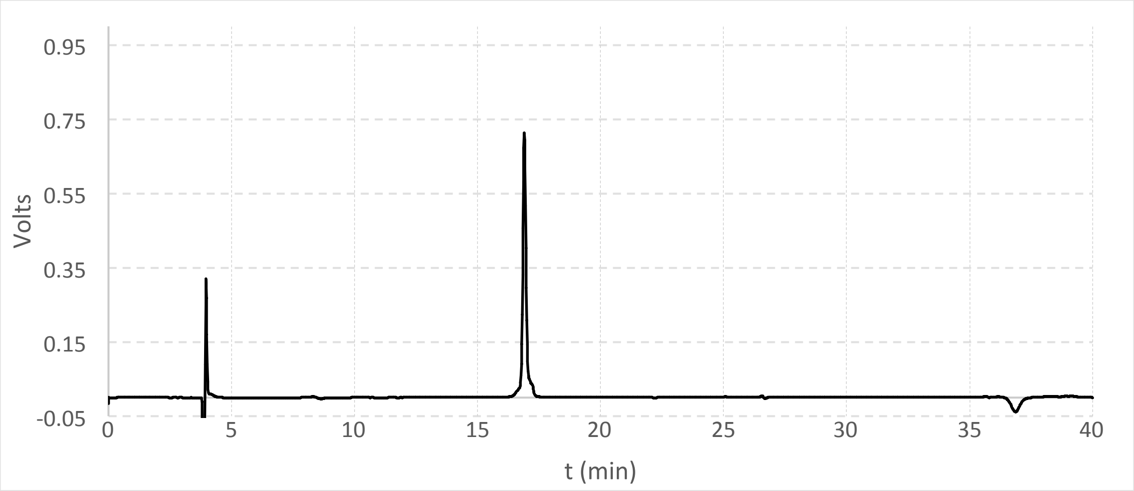

P4-C_{10}

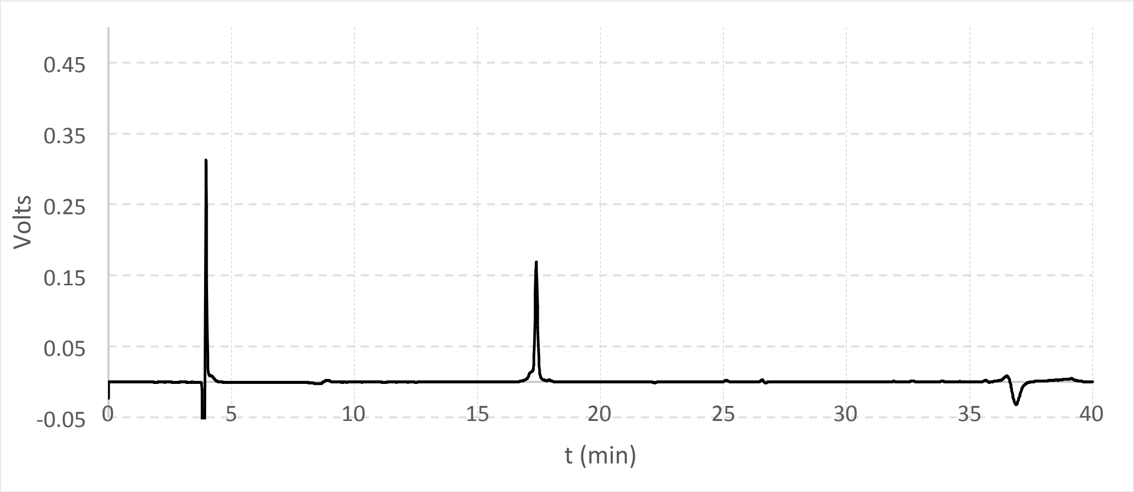

C_{10}-P6

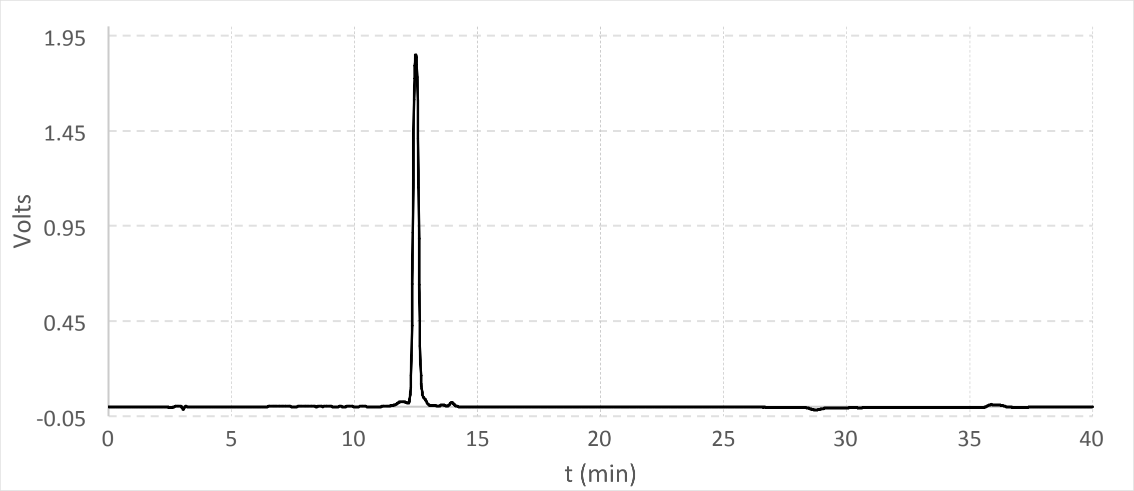

P6-C_{10}

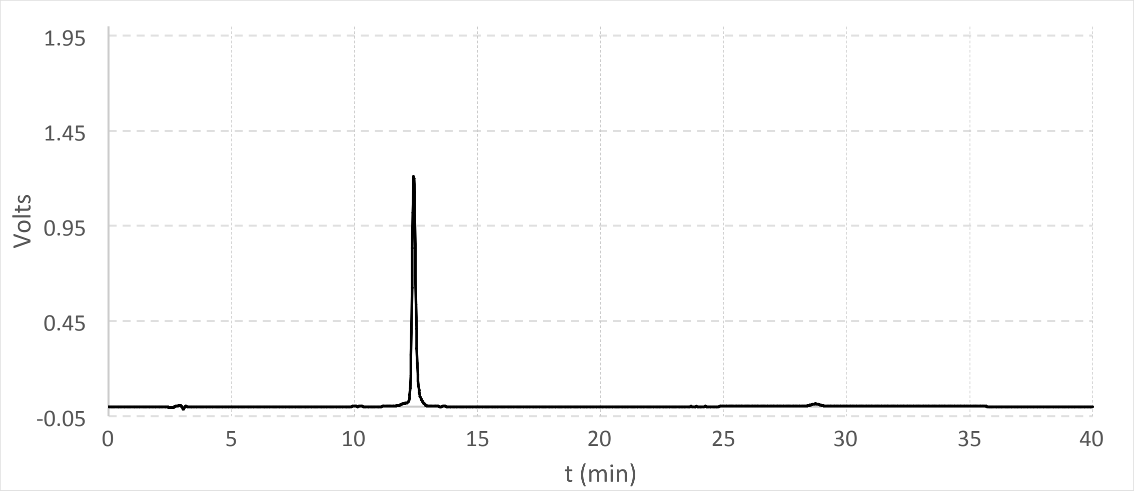

P8

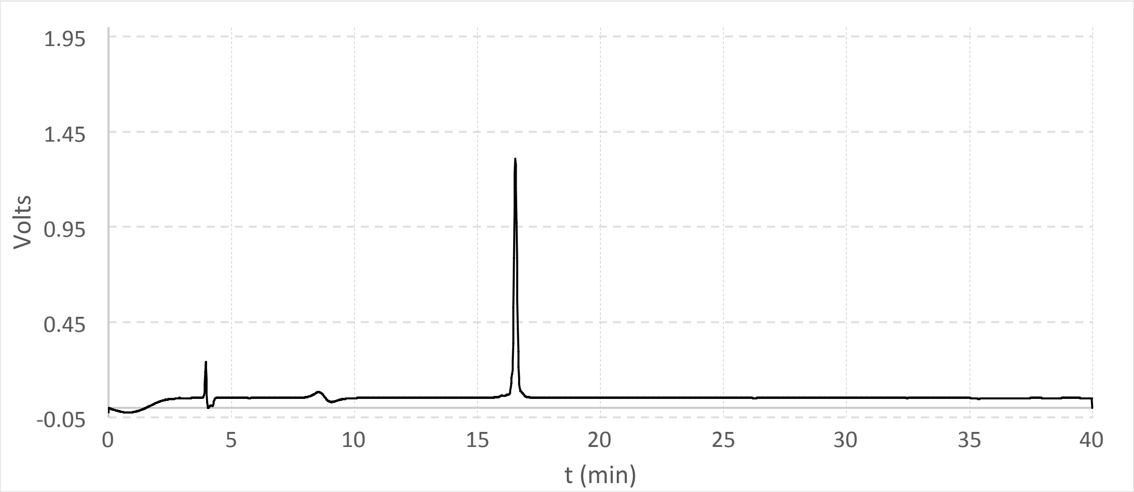

P8_L3A

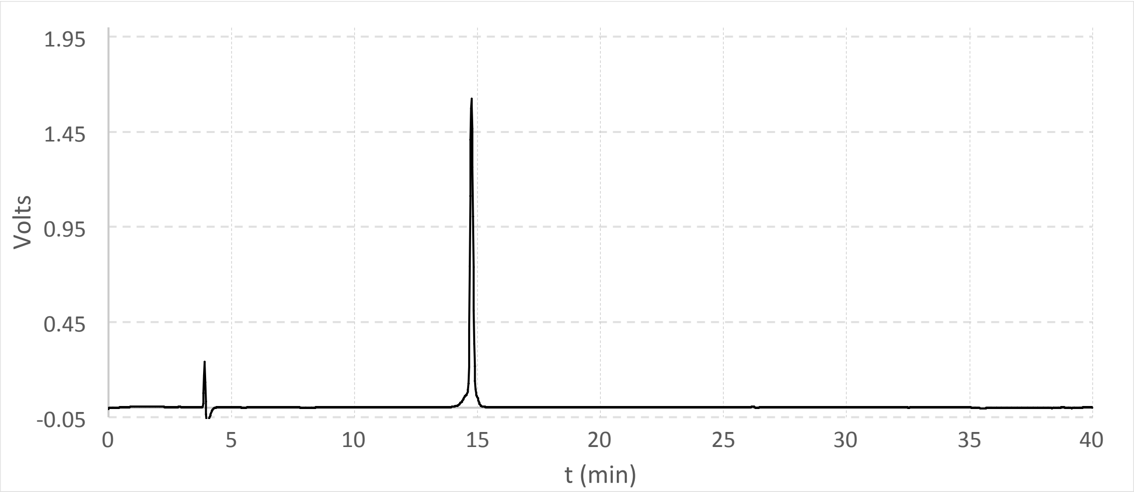

P8_L4A

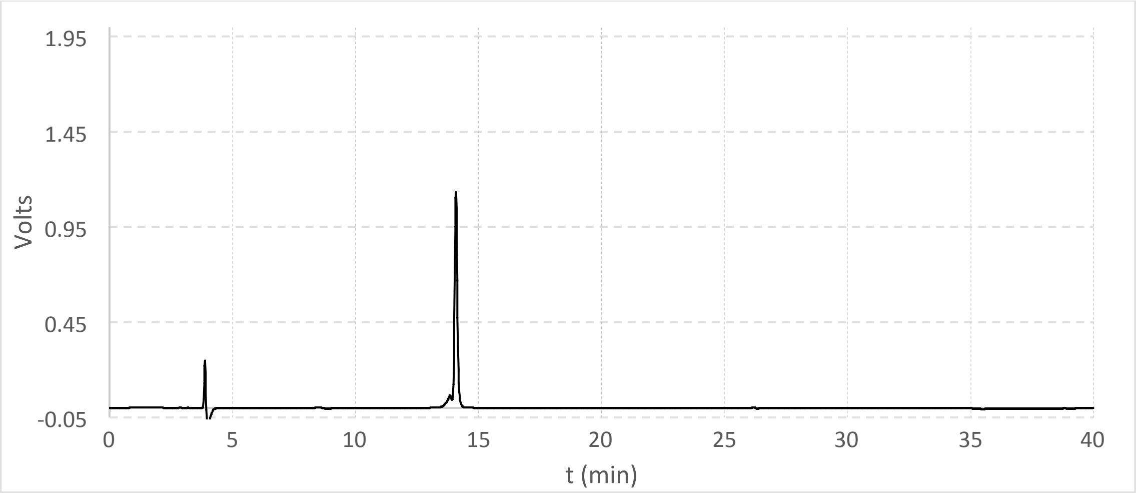

P8_Q5A

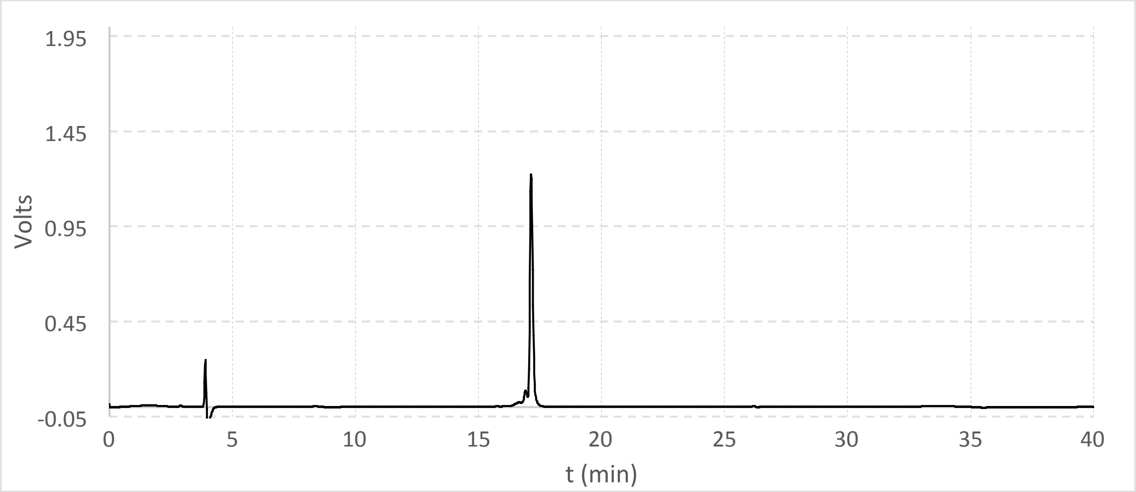

P8_S6A

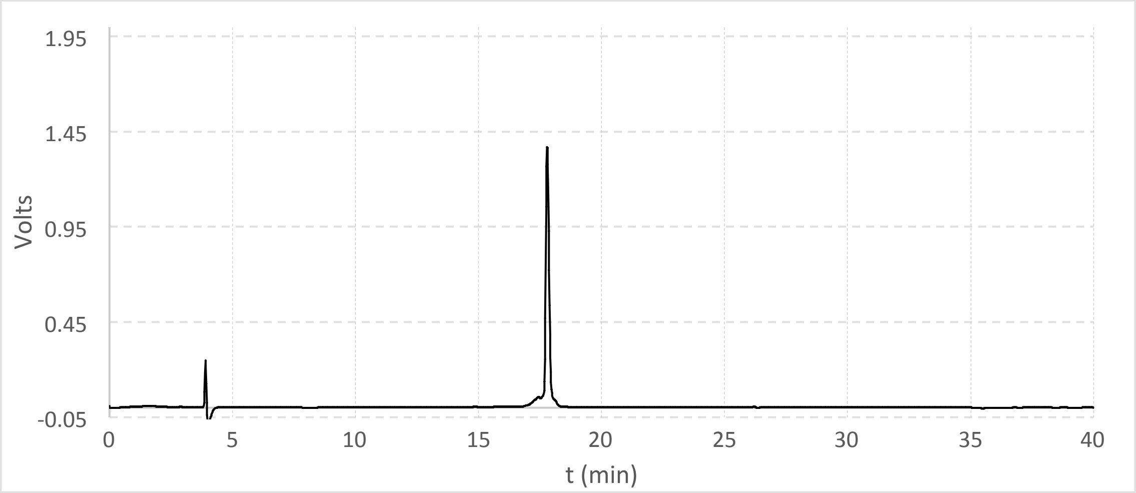

P8_L7A

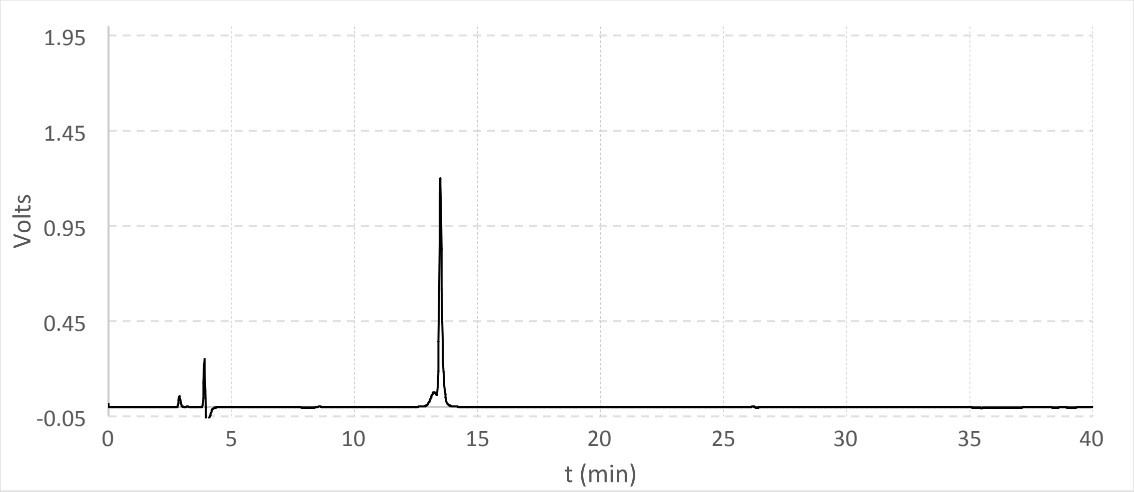

P8_L8A

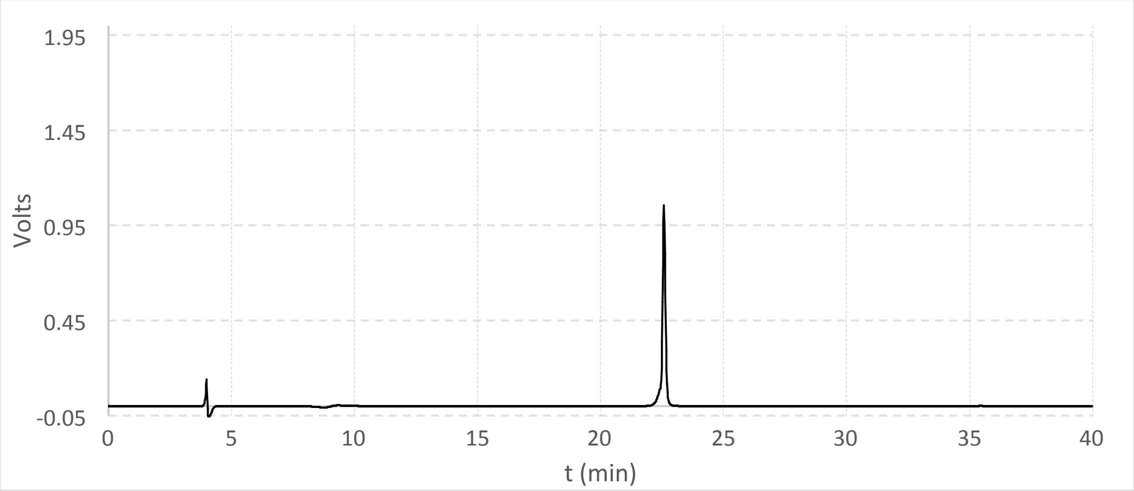

P8_P10A

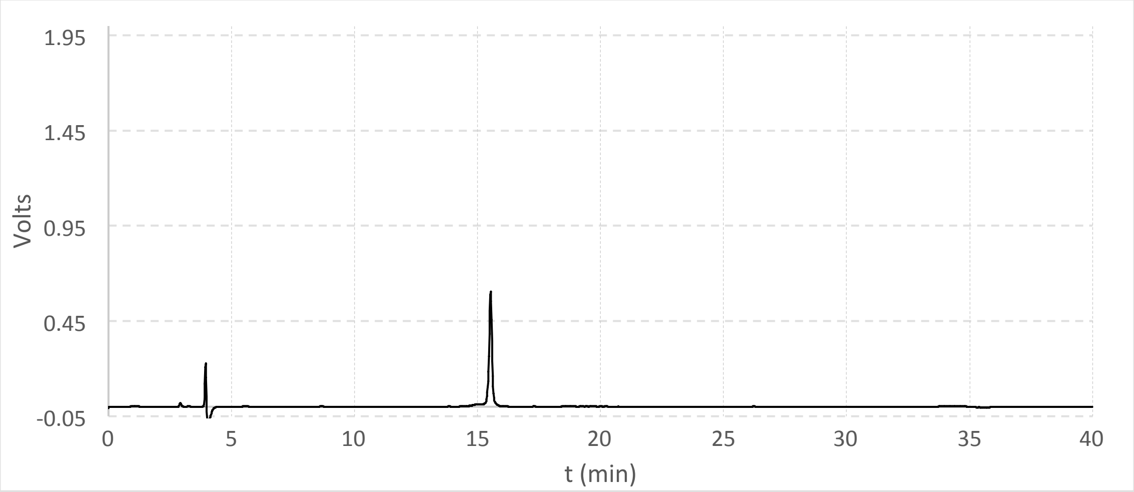

P8_Y11A

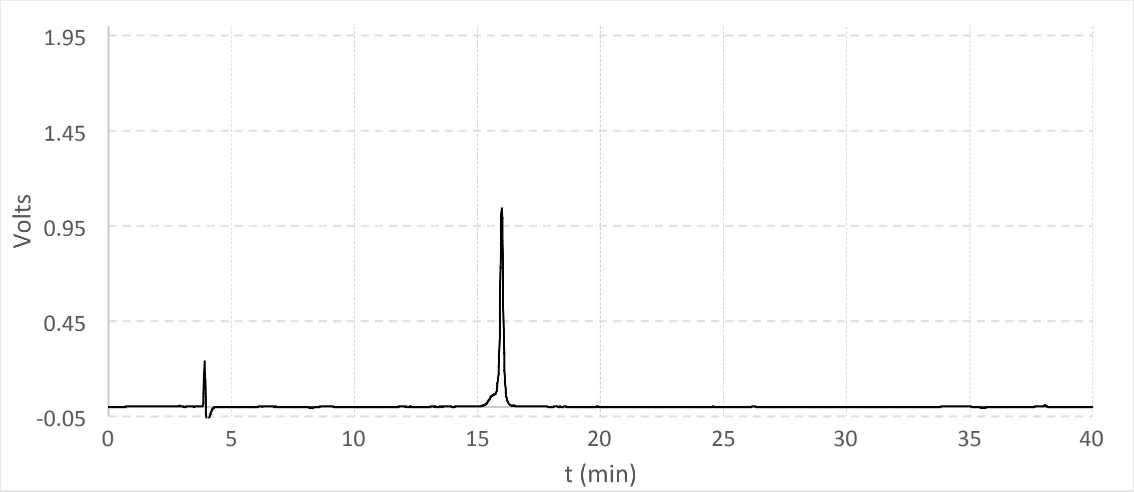

P8_S12A

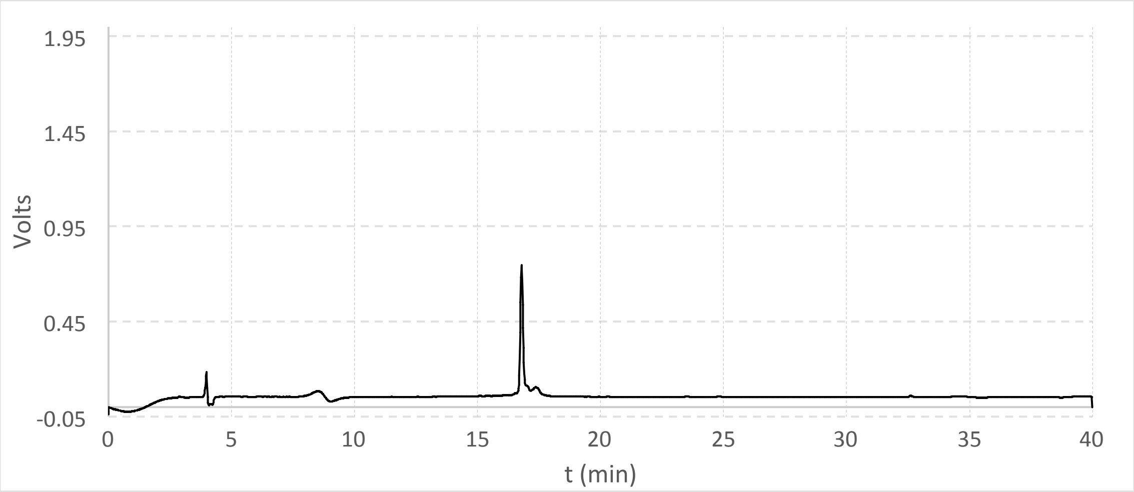

P8_T13A

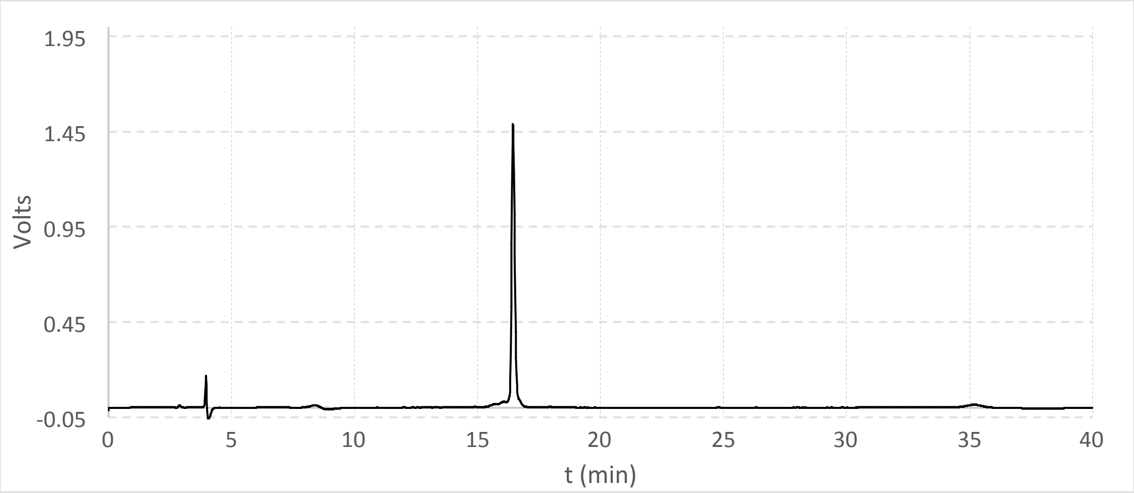

P8_H14A

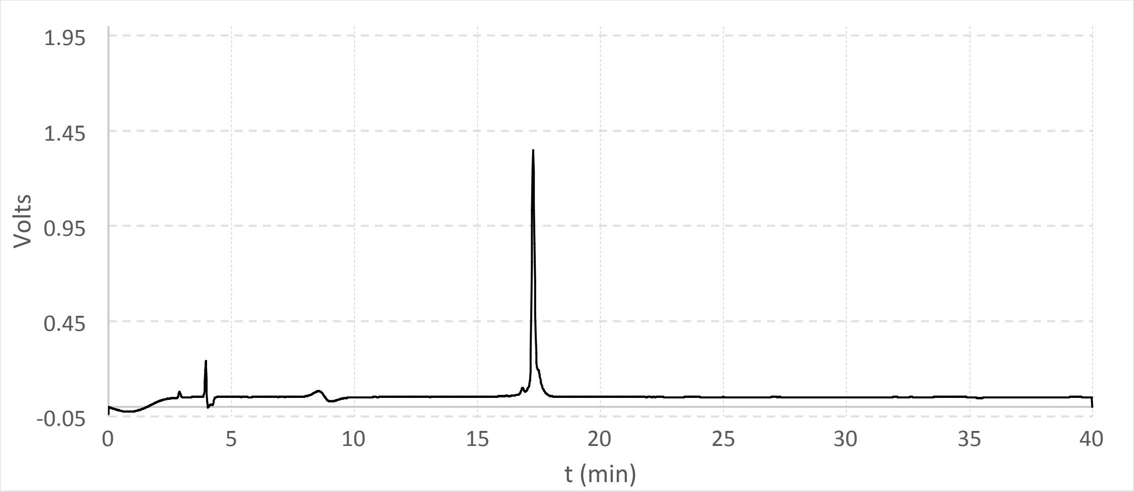

P8_R15A

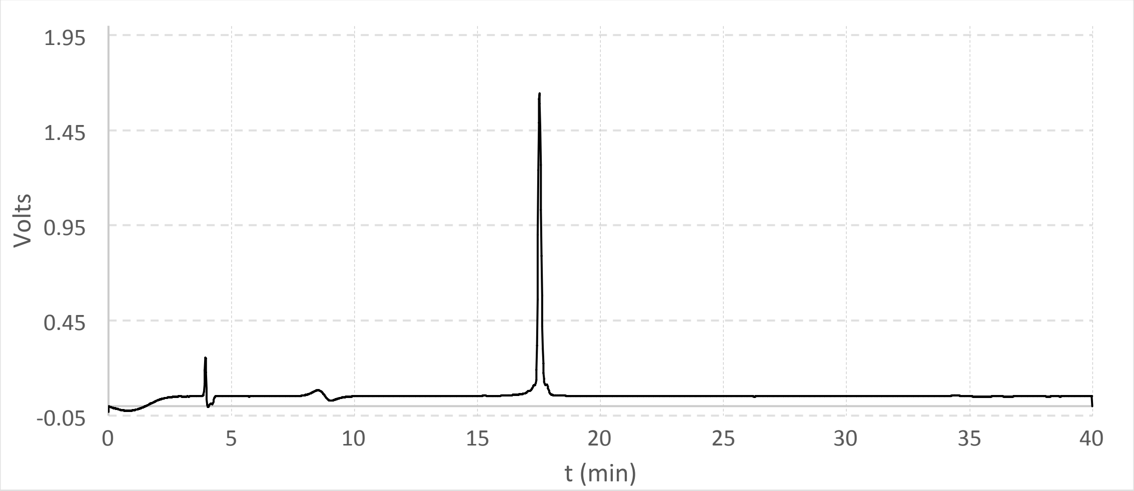

P8-C_6

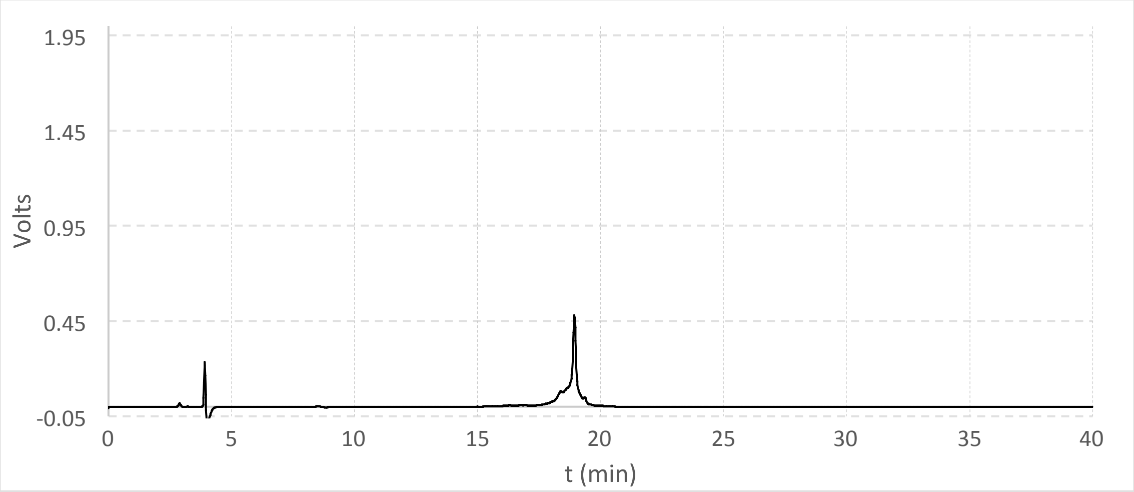

P8-C_8

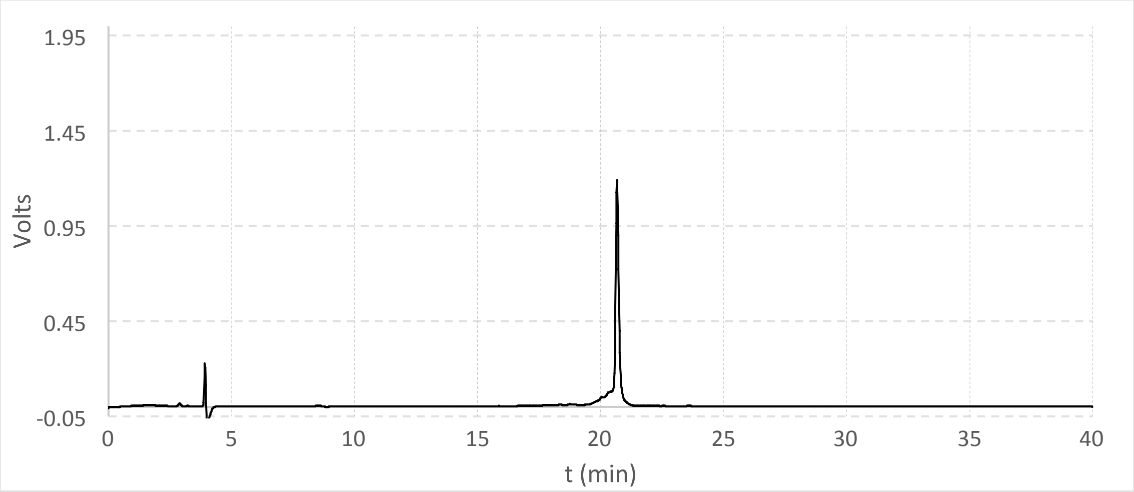

P8-C_{10}

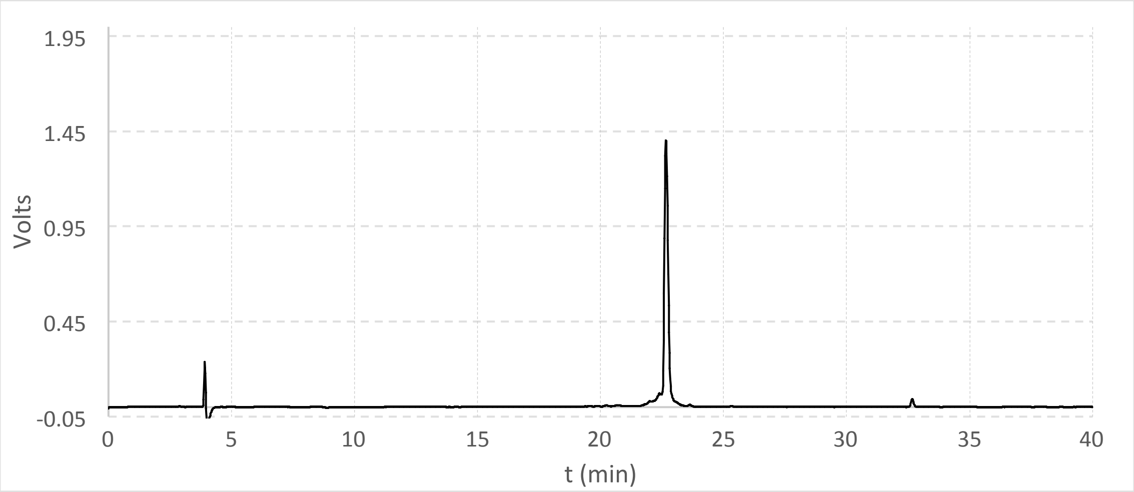

P8-C_{12}

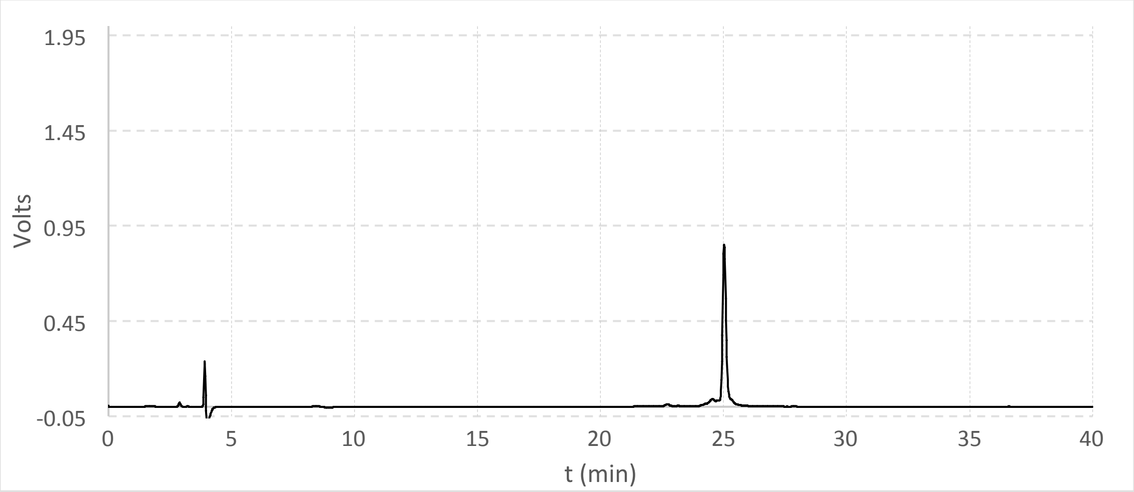

P8-C_{14}

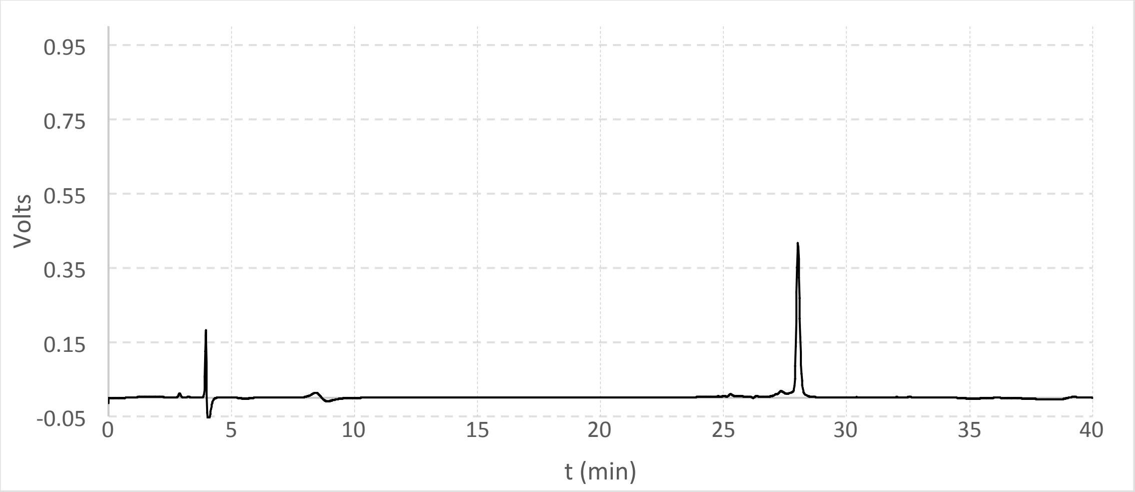

P8-L-C_{10}

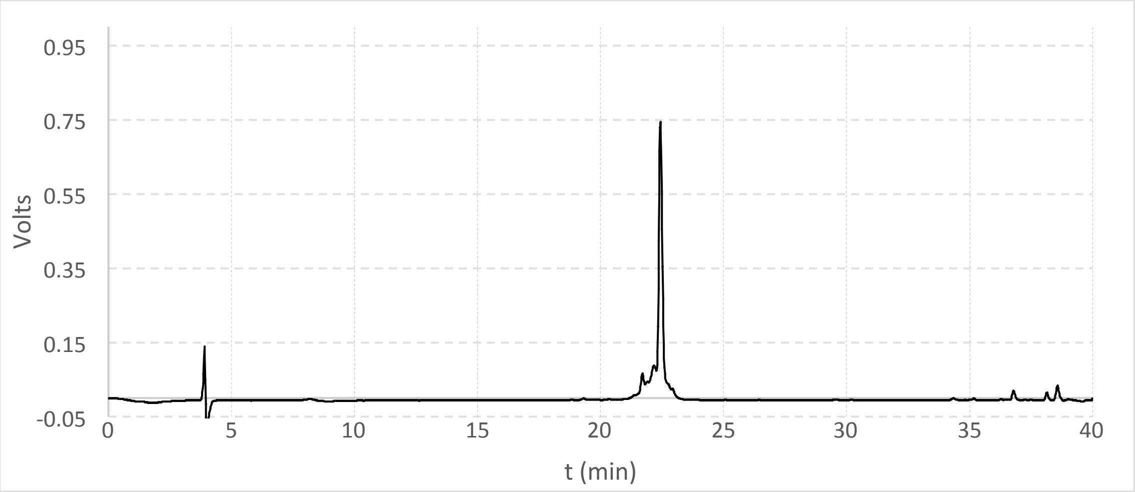

P8-S6R-R15A-C_{10}

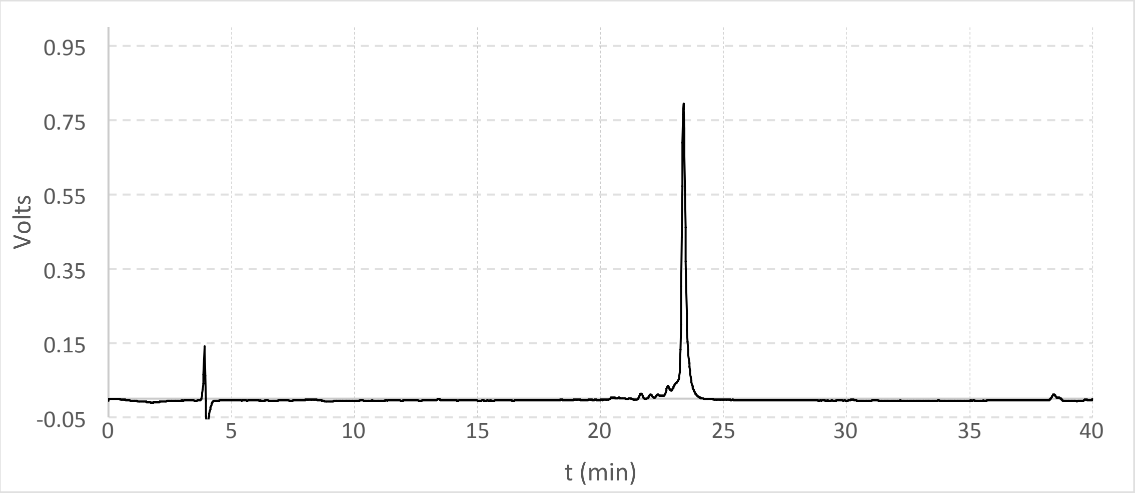

P8-P10R-R15A-C_{10}

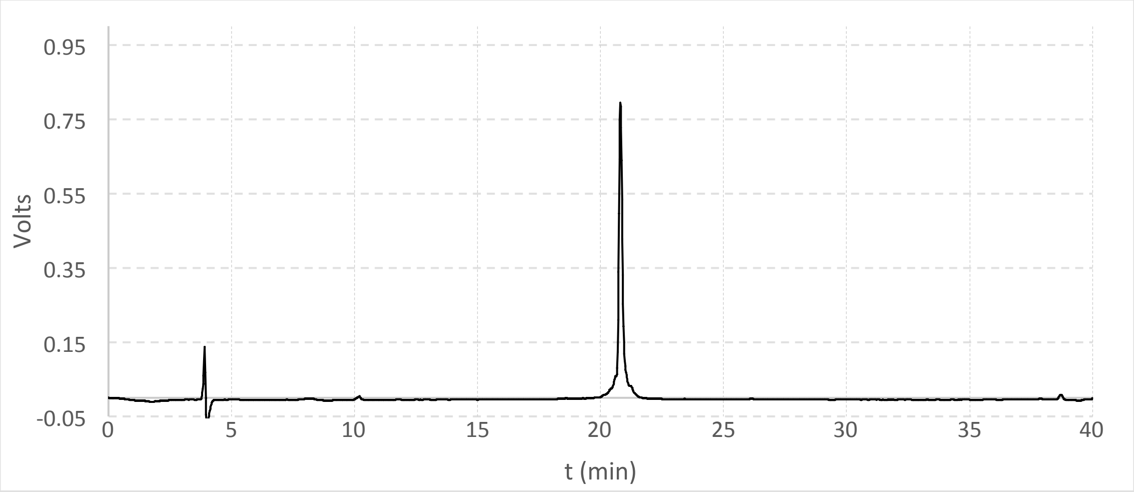

P8-H14R-R15A-C_{10}

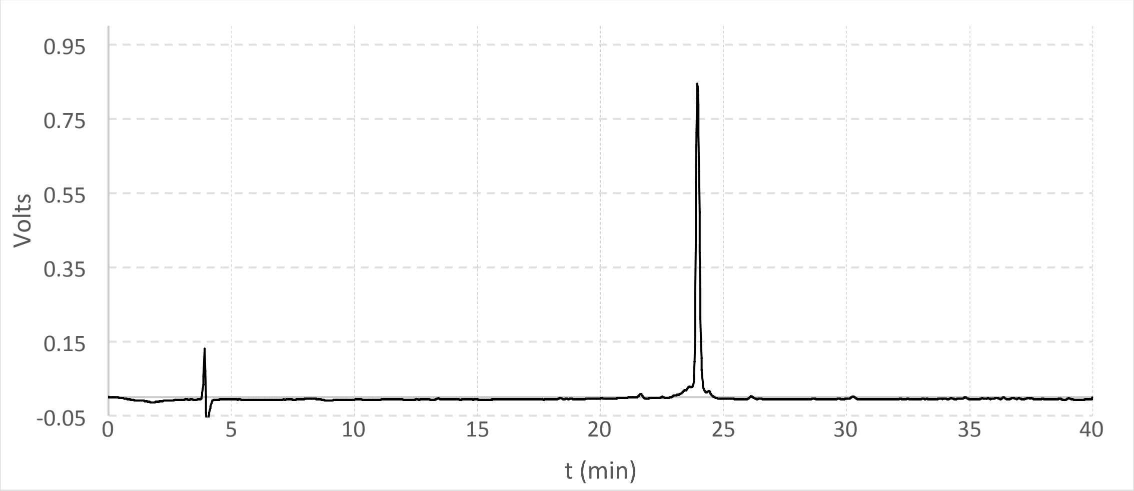

P8-R15A-C_{10}

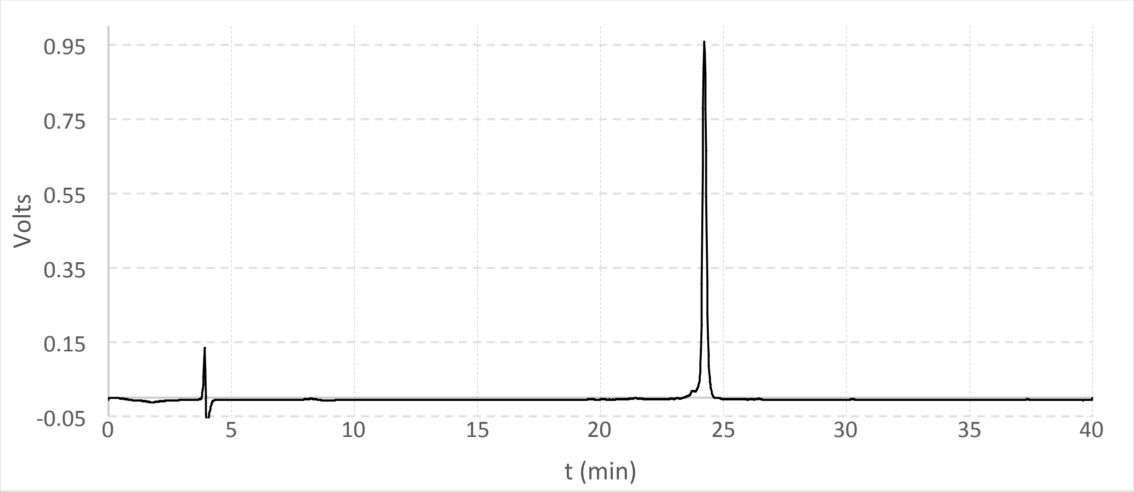

3.4 MS data for all peptides

Supplemental table S5: MS analysis of all synthesized peptides. All measurements were done using ESI HRMS unless otherwise indicated.

Peptide	Calculated $[M+2H]^{2+}$	Found $[M+2H]^{2+}$
P1	1057.39	1058.40 [a]
P2	958.44	959.50 [a]
P3	1044.89	1045.25 [a]
P4	997.49	996.94 [a]
P5	-	-
P6	1048.44	1048.45 [a]
P7	990.40	991.15 [a]
P8	989.97	990.00 [a]
C_{10}-P4	1074.56	1075.55 [a]
P4-C_{10}	1067.57	1067.80 [a]
C_{10}-P6	1125.51	1124.75 [a]
P6-C_{10}	1118.52	1119.40 [a]
C_{10}-P8	1066.5469	1066.5494
P8-C_{10}	1059.5573	1059.5643
P8-C_6	1031.526	1031.5253
P8-C_8	1045.5416	1045.5472
P8-C_{12}	1073.5651	1073.5777
P8-C_{14}	1087.5886	1087.5922
P8-D-(L3A)	968.4550	968.4558
P8-D-(L4A)	968.4550	968.4538
P8-D-(Q5A)	960.9677	960.9668
P8-D-(S6A)	981.4810	981.4824
P8-D-(L7A)	968.4550	968.4536
P8-D-(L8A)	1935.9030[a]	1935.9052[b]
P8-D-(P10A)	976.4706	976.4709
P8-D-(Y11A)	943.4653	943.4690
P8-D-(S12A)	981.4810	981.4824
P8-D-(T13A)	974.4732	974.4778
P8-D-(H14A)	956.4675	956.4691
P8-D-(R15A)	946.9464	946.9485
P8-D-(R15A)-C_{10}	1017.0252	1017.0260
P8-D-(S6R, R15A)-C_{10}	1051.5592	1051.5618
P8-L-(S6R, R15A)-C_{10}	1051.5597	1051.5555
P8-D-(P10R, R15A)-C_{10}	1046.5489	1046.5435
P8-D-(H14R, R15A)-C_{10}	1026.5458	1026.5432

[a]Measured by ESI LRMS. [b]$[M+H]^+$

Section 4 Biological and biochemical experiments

4.1 Minimum inhibitory concentration assay

MIC values were determined by using the broth microdilution method. Peptides were dissolved first in DMSO and diluted with broth so that the final peptide concentration was 1024 µg/ml and the DMSO concentration was 4%. Peptide serial dilutions were made in TSB in a polypropylene 96-wells plate using 50 µl volumes. An overnight culture of the organism of interest was diluted to and OD_{600} of 0.01 and grown until they reach 0.5. The culture was then diluted to 1 x 10^6 CFU/ml and 50 µl of this suspension was added to each well. The highest concentrations were then 512 µg/ml peptide and 2% DMSO. The plates were incubated at the preferred growth temperature of the organism that was used and 250 rpm. After 16 h the MIC was determined visually. When determining the MICs of the lipidated peptides, the lipopeptides were first dissolved in DMSO and diluted with TSB so that the peptide concentration was 128 µg/ml and the DMSO concentration was 4%. After inoculation the highest peptide concentration evaluated was 64 µg/ml and the DMSO concentration was 2%. A DMSO control was tested for each strain to ensure the DMSO did not affect the growth of the particular strain.

4.2 Hemolysis assay

Sheep blood in Alsever's solution (2 mL) was diluted with PBS (13 mL) and centrifuged (2,000 RPM, 5 min). The supernatant was discarded and the cells were washed with another 13 mL of PBS and centrifuged. Washing was continued until the supernatant was clear. After the final wash the supernatant was discarded and the packed cells were kept on ice. Serial dilutions of each peptide were made in 50 µl PBS with the highest tested concentration 64 µg/mL in PBS, 2% DMSO. The packed cells (150 µL) were suspended in PBS (10 mL) and 50 µL of this suspension was added to each well. A well plate column containing 0.1% triton x-100 in DI water was used as the 100% lysis control and a second column with PBS (with 2% DMSO) served as the 0% lysis control. After incubation at 37°C for 1h the plates were centrifuged (2,000 RPM, 5 min) and 25 µL of the supernatant was added to 100 µL DI water in a flat-bottom 96-wells plate (polystyrene). Hemolysis was determined by measuring the absorption at 414 nm to measure the amount of free hemoglobin.

4.3 UDP-MurNAc-pentapeptide accumulation assay

An overnight culture of *E. faecium* E980 was diluted 1000x in TSB and grown until an OD_{600} of 0.5. Chloramphenicol (130 µg/ml) was added and the culture was further incubated for 15 min. The culture was split into 5 ml portions and peptides were added at a final concentration of 10x MIC while the negative control received no peptide antibiotic. After further incubation for 30 min the cells were harvested by centrifugation at 3900 rpm for 5 min. The cells were transferred to glass tubes using 1 ml MilliQ water and kept in a boiling water bath for 15 min followed by rapid cooling on ice. The liquid was transferred to eppendorfs and cell debris was removed by centrifugation at 12000 rpm for 30 min. The supernatant was removed and lyophilized. The powder thus obtained was reconstituted in buffer A (50 mM NH_4HCO_3, 5 mM NEt_3, pH 8.3) and analysed by LCMS using a linear gradient of 0-25 % buffer B over 15 minutes (buffer B: MeOH). Pure UDP-MurNAc-pentapeptide was used as a reference.

4.4 Antagonization assays

Stock solutions of each peptide antibiotic were prepared so that their final concentration in the assay would be 4x MIC. Peptides were transferred to a 96 wells plate and lipid II or C_{15}-P (farnesyl-phosphate) was added at 4 molar equivalents in a final volume of 50 µl. Each well received 50 µl of bacterial suspension (*M. luteus*) at 1 x 10^6 CFU/ml and the plates were incubated for 16 h at 250 rpm and 30°C. Growth was observed visually (see supplemental table S10 for results).

4.5 Results of biological assays

Supplemental table S6. MIC values of lipidated phage display hits

	MIC (µg/ml)		
Peptide	*M. luteus*	*B. subtilis*	*E. faecium* (VRE 155)
C_{10}-P4	n.a.	n.a.	n.a.
P4-C_{10}	8	8-16	n.a.
C_{10}-P6	4	n.a.	n.a.
P6-C_{10}	2	n.a.	n.a.
C_{10}-P8-D	n.a.	n.a.	n.a.
**P8-D-C_{10}**	**4**	**8-16**	**4**
nisin	<1	4	<1

n.a. = not active (highest tested conc 64 µg/ml) .

Supplemental table S7: Results of MIC and hemolysis experiments using P8 with varying lipids

	MIC (µg/ml)		Hemolysis
Peptide	*M. luteus*	VRE E155	32 µg/ml (after 1 h)
P8-D-C_6	32	16-32	n.t.
P8-D-C_8	16	8-16	n.t.
P8-D-C_{10}	4	4	6 %
P8-D-C_{12}	4-8	8	9 %
P8-D-C_{14}	4-64[a]	4-64[a]	12 %

n.a. = not tested, [a]due to the poor solubility of the peptide irreproducible results were obtained.

Supplemental table S8: MIC results of Ala scan of P8-D. Activity determined against *M. luteus*

Peptide	Sequence	MIC (µg/ml)
P8-D	ACLLQSLLCPYSTHRCG	128
P8-D-L3A	ACALQSLLCPYSTHRCG	>512
P8-D-L4A	ACLAQSLLCPYSTHRCG	>512
P8-D-Q5A	ACLLASLLCPYSTHRCG	>512
P8-D-S6A	ACLLQALLCPYSTHRCG	64-128
P8-D-L7A	ACLLQSALCPYSTHRCG	512
P8-D-L8A	ACLLQSLACPYSTHRCG	128-256
P8-D-P10A	ACLLQSLLCAYSTHRCG	128
P8-D-Y11A	ACLLQSLLCPASTHRCG	512
P8-D-S12A	ACLLQSLLCPYATHRCG	512
P8-D-T13A	ACLLQSLLCPYSAHRCG	512
P8-D-H14A	ACLLQSLLCPYSTARCG	64-128
P8-D-R15A	ACLLQSLLCPYSTHACG	128-256

Supplemental table S9: Antibacterial activity of lipopeptides identified against various Gram-positive bacteria including a panel of vancomycin-resistant strains[a]

Peptide	*M. luteus* (NCTC2665)	*B. subtilis* (168)	*S. pyogenes* (5448)	*S. epidermidis* (1587)	*E. faecium* (E980)	*E. faecium* (VRE155)[b]	*E. faecium* (VRE0321)[b]	*E. faecium* (VRE2297)[b]	*E. faecium* (VRE7391)[b]	Hemolysis (%)[c]
P8-D-C_{10}	3.8	7.6-15.1	7.6	60.5	30.3	3.8	3.8	7.6-15.1	7.6	6
P8-D-(R15A)-C_{10}	3.9	>63.1	15.8	>63.1	63.1	15.8	7.9-15.8	>63.1	>63.1	0
P8-D-(H14R, R15A)-C_{10}	3.9	15.6	15.6	>62.4	31.2	7.8-15.6	7.8-15.6	15.6	7.8-15.6	2
P8-D-(P10R, R15A)-C_{10}	3.8	7.7	15.3	>61.2	30.6	7.7	15.3	30.6	15.3	0
P8-D-(S6R, R15A)-C_{10}	3.8	7.6	7.6-15.2	60.9	30.5	7.6	7.6	15.2	15.2	0
P8-L-(S6R, R15A)-C_{10}	3.8	7.6	7.6	30.5	30.5	7.6	7.6	15.2	15.2	0
Vancomycin	<0.7	<0.7	<0.7	1.4	<0.7	44.2	>44.2	>44.2	>44.2	ND
Nisin	<0.3	1.2	<0.3	1.2	9.5	<0.3	0.3	2.4	0.6	ND

[a]MIC values reported in µM. [b]Vancomycin-resistant *Enterococcus faecium* isolates from hospitalized patients. [c]Hemolysis measured after 1 hour incubation at 37°C with lipopeptides applied at 32 µg/mL.

Supplemental table S10. Lipid II antagonization assay results

Antibiotic compound	Result with indicated antagonist[a]		
	Lipid II	C_{55}-PP	C_{15}-P[b]
P8-C_{10}	+	+	-
P8-D-(S6R, R15A)-C_{10}	+	+	-
P8-L-(S6R, R15A)-C_{10}	+	+	-
nisin	+	+	-

[a] + = antagonization of antibiotic activity; - = no antagonization of antibiotic activity; [b] C_{15}-P was used as a negative control (C_{15}-P is a watersoluble analogue of the lipid II precursor undecaprenol phosphate).

4.6 Scanning Electron Microscopy

E. faecium E155 was inoculated at an OD_{600nm} of 0.05 into 300 µL of cation adjusted TSB containing P8-D-(S6R, R15A)-C_{10} or nisin at (both a 2x MIC) along with 1% glucose and 1% DMSO. An untreated culture was used as control. Following overnight incubation (ca. 16 hr) bacterial cells were fixed for 15 minutes with 1% (v/v) glutaraldehyde in phosphate buffered saline (PBS) at room temperature on poly-L-lysine covered glass slides (12 mm diameter). Samples were washed twice with PBS to remove excess fixative and were subsequently serially dehydrated by consecutive incubations in 1 ml of 25% (v/v) and 50% (v/v) ethanol-PBS, 75% (v/v) and 90% (v/v) ethanol-H_2O, and 100% ethanol (2x), followed by 50% ethanol-hexamethyldisilazane (HMDS) and 100% HMDS. The glass slides were removed from the 100% HMDS and air-dried overnight at room temperature. After overnight evaporation of HMDS, samples were mounted on 12 mm specimen stubs (12 mm, Agar Scientific) and bacteria coated with gold to 1 nm using a Quorum Q150R sputter coater at 20 mA prior to examination with a Phenom PRO Table-top scanning electron microscope (PhenomWorld).
